# Green-Light Photocatalysis: Borylated Benzo[*c*][1,2,5]thiadiazole (BTZ) Enables Phosphorylation of Quinoline Derivatives

**DOI:** 10.1021/acs.joc.5c01367

**Published:** 2025-09-29

**Authors:** Leonardo Amicosante, Dominic Taylor, Luca Craciunescu, Andrew W. Prentice, Adilet Zhakeyev, Bence Szabó, Georgina M. Rosair, Martin J. Paterson, Scott J. Dalgarno, Filipe Vilela

**Affiliations:** Institute of Chemical Sciences, School of Engineering and Physical Sciences, 3120 Heriot-Watt University, Edinburgh EH14 4AS, U.K.

## Abstract

A library of 10 novel organophotocatalysts (**ORG-PRCs**) has been prepared by the one-pot, two-step *ortho*-borylation of 4,7-diarylbenzo­[*c*]­[1,2,5]­thiadiazoles (**BTZs**). The borylation reaction was accompanied by a substantial bathochromic shift in both the absorption and emission spectra (up to 142 nm), allowing these photocatalysts to operate using low-energy green light instead of the high-energy near-UV light that **BTZ** photocatalysts typically require. The library of photocatalysts was tested using the phosphorylation of quinoline compounds under both batch and recycle flow conditions, achieving up to 40 and 73% conversion, respectively, in 4 h. The versatility of the recycle flow system was further tested by developing a sequential two-step phosphorylation followed by a Minisci coupling procedure using a dual photocatalyst system. This allowed automated production of the target antibacterial phosphorylated quinoline derivative with a total conversion of 64% from abundant, inexpensive, and late-stage modifiable starting materials in a streamlined process.

## Graphical abstract

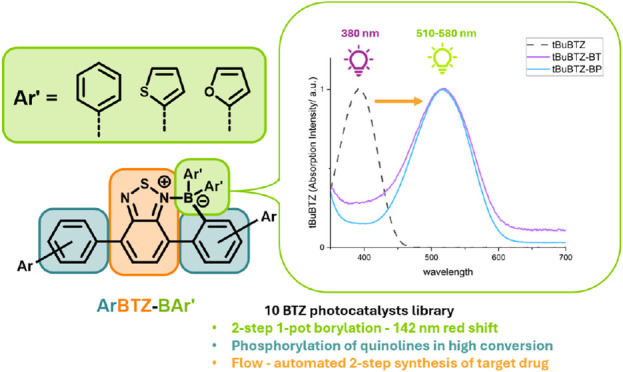

## Introduction

In recent years, there has been increasing interest in phosphorylated quinoline-based moieties within pharmaceutical chemistry and drug discovery, particularly for their potential applications in antiviral and antibacterial therapies, as well as cancer treatment.^1–7^ However, the synthesis of these compounds using traditional methods often requires protecting groups, elevated temperatures, extended reaction times, and multistep processes with expensive purification at each stage, such as the reported procedure by Isshiki et al. (using a nickel catalyst at 170 °C for 18 h) and in a recent patent by Qiu et al. (employing a multistep approach to individually form a phosphorylated quinoline derivative).^5,8,9^ Hence, greener, milder, and more atom-economic routes have been investigated by Cui et al., with a growing interest in photocatalysis for such applications, more specifically employing iridium-based photoredox catalysts (**PRCs**).^10^ Following these results, herein, the authors present the use of alternative organic PRCs based on benzo­[*c*]­[1,2,5]­thiadiazole (**BTZ**) to achieve these important target molecules in a greener fashion (Figure 1).

**Figure 1:**
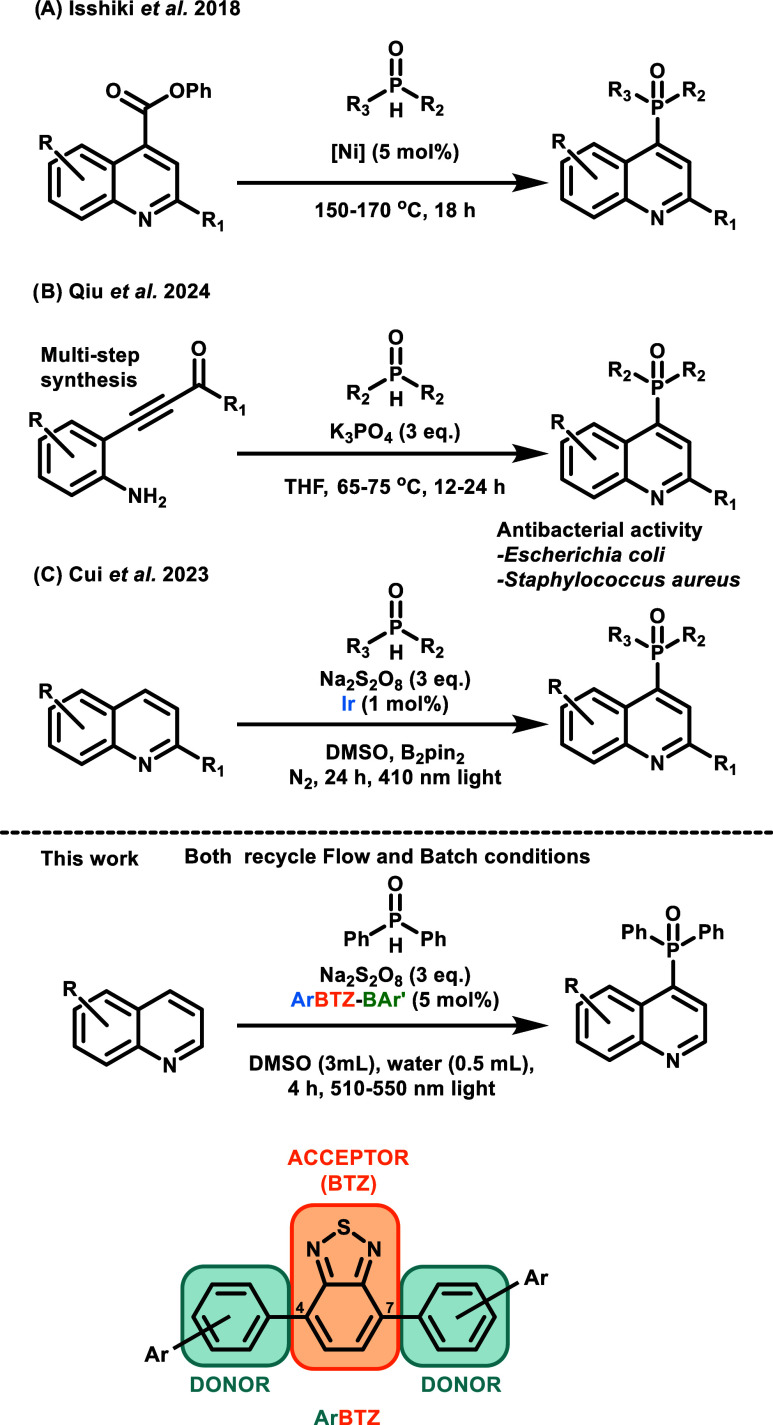
Selection of synthetic paths toward C-4-phosphorylated quinolines. (A) Isshiki et al.,^8^ (B) Qiu et al.,^5^ and (C) Cui et al.^10^ General structure of 4,7-diarylbenzo­[*c*]­[1,2,5]­thiadiazole (**BTZ**). The central benzothiadiazole block acts as the electron acceptor, and the conjugated aromatic units at positions 4 and 7 act as the electron donor blocks.

Photocatalysis has certainly proven to offer a plethora of green advantages to the above-mentioned reactions, including the great availability of solar light and its traceless nature.^11–13^ However, while state-of-the-art iridium-based **PRCs** present great efficiencies, they also require near-UV, high-energy light (which is less abundant from the solar spectrum),^14^ and increasingly high costs due to the great rarity of such metals. Their scarcity, high toxicity, and costs lead to various sustainability and safety concerns regarding the scalability of photoreactions using iridium **PRCs** for an ever-growing pharmaceutical industry. In fact, it is important to mention that to date, only 6–8 tonnes of crude Ir metal are extracted every year, with prices per gram of the said metal on the rise.^12,15–18^

In search of more environmentally sustainable alternatives to the aforementioned species, great effort has been invested in the use of more abundant metals (such as titanium, iron, copper, and zinc),^18,19^ naturally occurring pigments such as chlorophyll,^20^ synthetic dyes such as methylene blue,^21^ and benign-by-design organophotoredox catalysts (**ORG-PRCs**) which are based on conjugated electron donor–acceptor (EDA) systems.^12,22–25^ The latter family of photoredox catalysts has drawn interest in the community primarily due to the higher biocompatibility of **ORG-PRCs**, which, unlike metal-based ones, can be safely employed in the late-stage synthesis of pharmaceuticals without the risk of metal contamination.^26^ In addition, they possess greater versatility and high tunability of their key photophysical properties upon structural synthetic modifications (i.e., the wavelength of maximum absorption (λ_abs_) and maximum emission (λ_em_), the rate of intersystem crossing (ν_ISC_), the photoluminescence quantum yield (PLQY, φ_f_), and the lifetime of an excited state (τ), as well as the redox potentials (*E*_red_ and *E*_ox_)).^27^ **BTZ**-based **ORG-PRCs** and derived materials have been proven extensively to deploy both biocompatibility and greater versatility in the above-mentioned properties upon changes in the electron donor–acceptor–donor (EDAD)-type structure (here, a selection of examples are reported) (Figure 1).^28–34^ In these examples, the electron–acceptor block is constituted by the central **BTZ** unit, while the two π-conjugated electron-rich aryl groups are referred as electron donor blocks. Modification of the latter leads to a direct modification of the highest occupied molecular orbital (HOMO) and, to a lesser extent, the lowest unoccupied molecular orbital (LUMO) energy levels, which overall determines the HOMO–LUMO energy gap, corresponding then to the energy required by the **BTZ ORG-PRC** to reach the first excited state required for a photochemical process.^27^

However, **BTZ**-based **ORG-PRCs** often show limits in the high-energy near-UV absorption (380–450 nm).^28^ Moreover, the trend of excited-state redox potentials shows that the more electron-withdrawing the electron donor blocks are (compared to a benchmark phenyl group), the higher the E*_red_ (excited-state reduction potential) and hence the wider the range of photo-oxidations that can be achieved by the **BTZ ORG-PRC**. On the other hand, the downside of such electron-deficient donor blocks is that they further increase the HOMO–LUMO energy gap. Hence, they present a hypsochromic shift in the absorption and emission energies, leading to them absorbing mostly in the UV region rather than in the visible region of the electromagnetic spectrum. Alternatively, when electron-rich donor blocks were employed (e.g., electron-donating group-substituted phenyls or thiophenes), their E*_red_ also decreased, reducing the range of efficacy as an oxidant while simultaneously reducing its HOMO–LUMO gap (Figure 1).

Improvement of a photoredox process does not only rely on the **PRC** but can also be achieved by changing the reaction conditions or methodology or even by adopting more efficient ways in which light is delivered. For instance, flow chemistry could be contemplated as a valid strategy to mitigate the above-mentioned gap in reactivity between the iridium state-of-the-art photocatalysts and their **BTZ** counterparts, as it has gained growing attention in the photochemistry community in the past decade due to its particular enhancement of efficiency of photoreactions.^14,33,35,36^ This effect is mostly due to the Beer–Lambert law shown in eq 1 (where *A* is the absorbance, *ε* is the molar absorption coefficient, *c* is the molar concentration of the reaction solution, and *l* is the optical path length in cm).

1 A=εcl

This causes the attenuation of light through a photocatalyst solution to be directly proportional to the depth of the glassware adopted, which determines the path length. Thus, in batch photochemical reactions, irradiation through traditional glassware (such as round-bottomed flasks and vials) is inefficient and can become a limiting factor in the rate of photoreactions. In contrast, if the same volume of the reaction mixture is instead pumped through thin tubing, the surface area of the solution is heavily increased (as the path length is reduced) and consequentially, the irradiation is homogeneously increased throughout. Furthermore, extensive literature has been published on monitoring chemical processes via in-line, nondestructive analysis of the reaction mixture using selected analytical techniques, most commonly benchtop NMR or UV–visible spectroscopy.^37–39^ Alternatively, at-line methods involve sampling the reaction mixture, typically through a valve, for analysis with a chosen instrument without returning the sample to the main flow stream.^40,41^ This grants a more precise assessment of a reaction profile, as it facilitates a vast increase in analytical data collection obtained during a single experiment. Moreover, such real-time monitoring methods achieve this in an automated way, thus reducing human error during tests.

However, employment of flow systems alone cannot solve the *a priori* limitations in the high energy absorption restrictions affecting both **BTZ**- and Ir-based **ORG-PRCs**, which for this type of an aromatic compound could lead to undesired reactivity. Thus, this should be addressed at an earlier stage. Substantial bathochromic shifts (130–170 nm) of **BTZ** chromophores have already been demonstrated by Ingleson and co-workers by utilizing a simple electrophilic aromatic borylation strategy involving BCl_3_, followed by transmetalation with aryl-tin and aryl-zinc reagents.^42–44^ This led to a ring formation between the nitrogen atom of the **BTZ** unit and the *ortho*-position in the electron donor block, while the transmetalation step ensures water and moisture stability by substituting the chlorine atoms at the boron bridge with aryl groups from an organostannane reagent (Scheme 1).

**Scheme 1:**
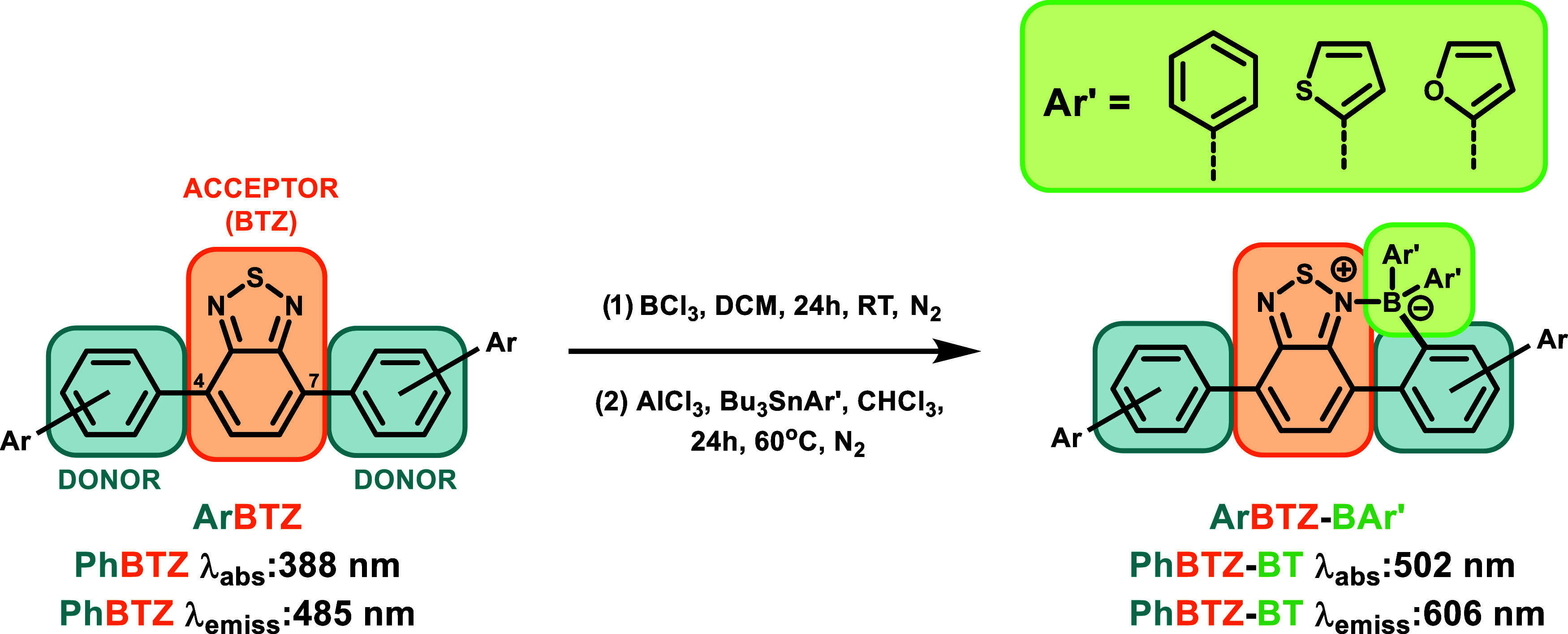
Synthesis of Borylated **ORG-PRCs** via *the ortho*-Borylation Reaction Followed by Transmetalation^42–44^

Recognizing that the nonborylated **BTZ ORG-PRCs** (henceforth referred to as “parent” **BTZ**s) are ineffective in the valuable phosphorylation of quinolines, we decided to explore the efficacy of a new class of **BTZ ORG-PRCs** derived from this borylation. Herein, we report the development of 10 novel **BTZ**-based **ORG-PRCs** employed in the selective phosphorylation reaction in recycle flow for a greener synthesis of antibacterial drugs and antiviral and tumoricidal drug analogues from abundant and affordable starting materials.^1–7^

## Methods, Results, and Discussion

### Synthesis of the **BTZ** Library

The synthetic path followed for this library involved a two-step, one-pot synthesis from the respective 4,7-diaryl**BTZ** used for each member. The first step involved the regioselective electrophilic aromatic borylation at the *ortho*-position on the electron donor block using BCl_3_ in anhydrous dichloromethane (DCM) at room temperature under a stream of nitrogen over 24 h with quantitative yields obtained. The following step involved an AlCl_3_-catalyzed transmetalation of the B–Cl bonds using various organostannane reagents (phenyl (**P**), thiophenyl (**T**), or furanyl (**F**)) in anhydrous chloroform or DCM under reflux for 24 h to obtain the library of bench-stable catalysts. As mentioned above, the transmetalation step was found to be necessary for water stability, as the −BCl_2_ intermediate systematically forms a cyclic triboronic ester between three **BTZ** molecules when in protic solvents or in the presence of water (Figure S63). Moreover, the transmetalation was observed to occur in both symmetric and asymmetric **BTZs** (i.e., with two aryl electron donor blocks coupled to the central **BTZ** or only one at position 4), hence showing potential versatility for further coupling and a more complex asymmetric EDAD′-type structure (Figure S65). The parent **BTZ** **ORG-PRCs** were also obtained in two steps starting from cheap and commercially available **BTZ**, which was subsequently brominated at the 4- and 7-positions (see Scheme 1) according to literature procedures.^45–47^ 4,7-Dibromo**BTZ** was then used in Suzuki–Miyaura cross-coupling reactions with desired aryl-boronic acids depending on the donor block being targeted, e.g., phenyl (**Ph**), 4-*tert*-butyl phenyl (**tBu**), 2-naphthalene (**2Nap**), 10-dioctylfluorenyl (**Flu**), and thiophenyl (**Tp**).^28^ The library of photocatalysts was then obtained by cross-coupling of the **BTZ** core with the above-mentioned 5 different electron donor blocks (hence starting from 5 parent **BTZs**) followed by borylation and transmetalation with the three Ar′ groups substituted onto the boron bridging atom after the borylation step. This resulted in a combination of 10 **ORG-PRCs** labeled **PhBTZ-BT**, **PhBTZ-BP**, **PhBTZ-BF**, **tBuBTZ-BT**, **tBuBTZ-BP**, **2NapBTZ-BT**, **2NapBTZ-BP**, **FluBTZ-BT**, **TpBTZ-BT**, and **TpBTZ-BP**, where the short-hand for the electron donor block is indicated as the prefix to **BTZ**, and the transmetalated aryl ring is indicated as the suffix (see Figure 2). No new safety risks were detected from the synthesis of the BTZ library; for more information regarding the full safety statement, please refer to the ESI document (page S-10, synthetic procedures).

**Figure 2:**
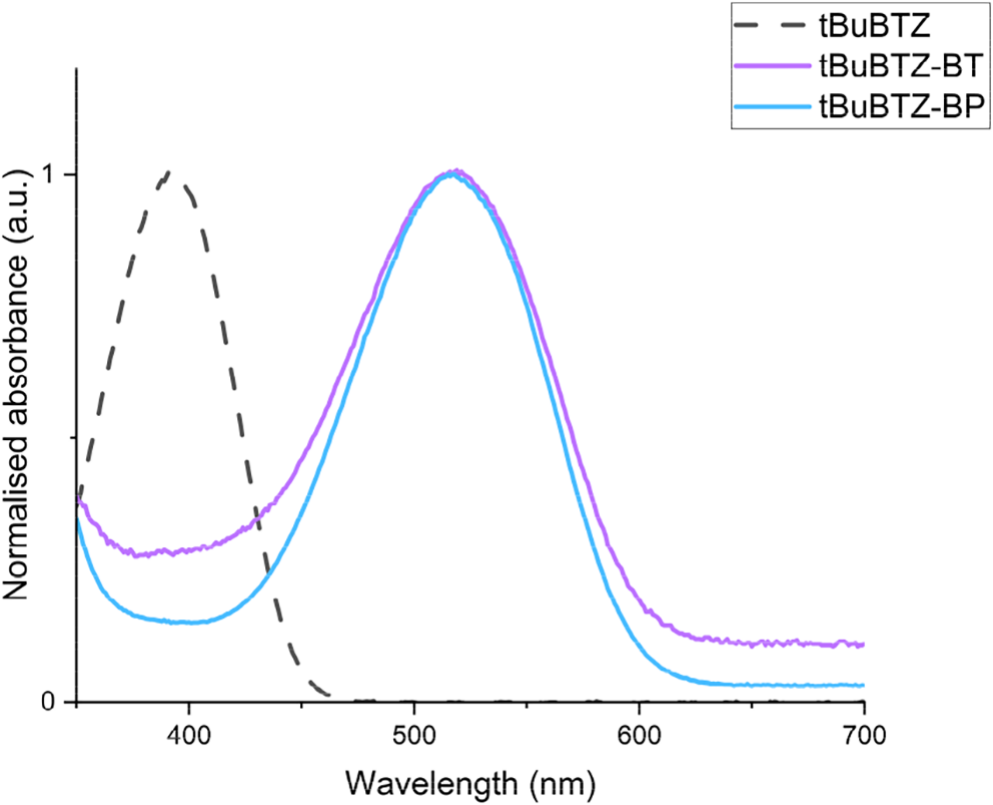
Stacked and normalized UV–vis absorption spectra of **tBuBTZ** (black, dotted), **tBuBTZ-BT** (purple), and **tBuBTZ-BP** (blue) from 350 to 700 nm. UV–vis absorption measurements were performed in CHCl_3_ at an arbitrary concentration.

### Photophysical and Optoelectronic Properties of Borylated **BTZ** Photocatalysts

The photophysical and optoelectronic properties of the **BTZ** library were strictly dependent on the structure of each **ORG-PRC**. The first and most impactful change that was observed upon borylation was a bathochromic shift of absorption (Δ*λ*_abs_) from a minimum of 110 nm for **PhBTZ-BP** to a maximum of 142 nm for **FluBTZ-BT** (Table S1). Interestingly, this trend corresponded to the increment of the electron-donating capability of the electron donor block (**Ph < tBu < 2Nap < Flu**), while the difference between the various −**BAr′** groups within the same electron donor starting material **BTZ** did not seem to significantly impact the absorption or emission (Figures 2 and 3).

**Figure 3:**
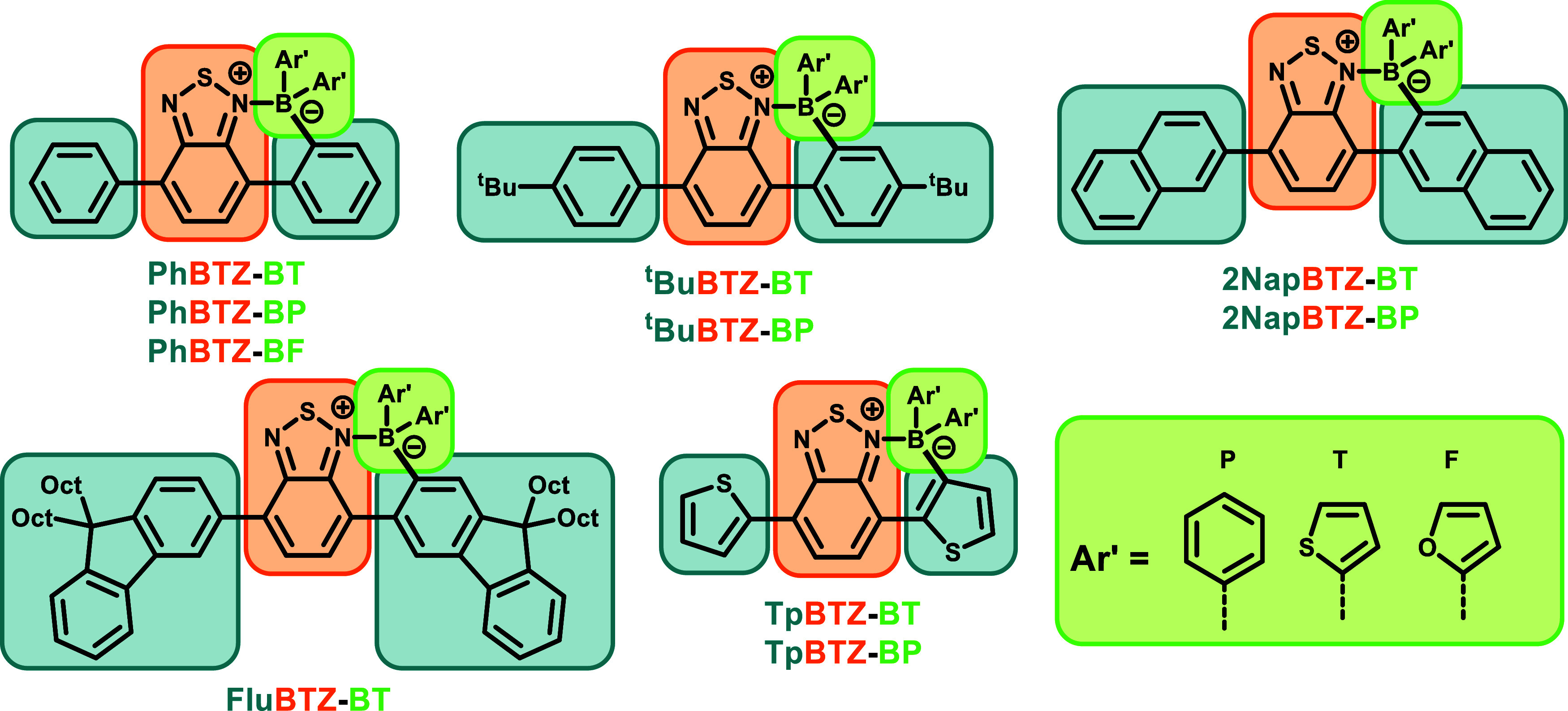
Library of borylated photocatalysts synthesized in this study with the following nomenclature: electron donor block-**BTZ** (electron acceptor block)-B (boron bridge)-aryl-substituted during transmetalation (*T* = thiophenyl, *P* = phenyl, *F* = furanyl).

The bathochromic shift, Δ*λ*_abs_, was thought to be due mostly to the effect of both the boron atom itself, as borylation is typically used to generate so-called deep LUMO materials, which could also affect the HOMO–LUMO gap in virtue of its electron-sink role,^48^ and the forced coplanarity induced by the boron bridge between the electron donor blocks and the acceptor **BTZ** moiety, as can be seen upon inspection of the single-crystal X-ray structure shown in Figure 4. In fact, as π-conjugation was extended by virtue of this structural modification, the HOMO–LUMO energy gap was reduced, leading to an overall lower energy required for excitation.

**Figure 4:**
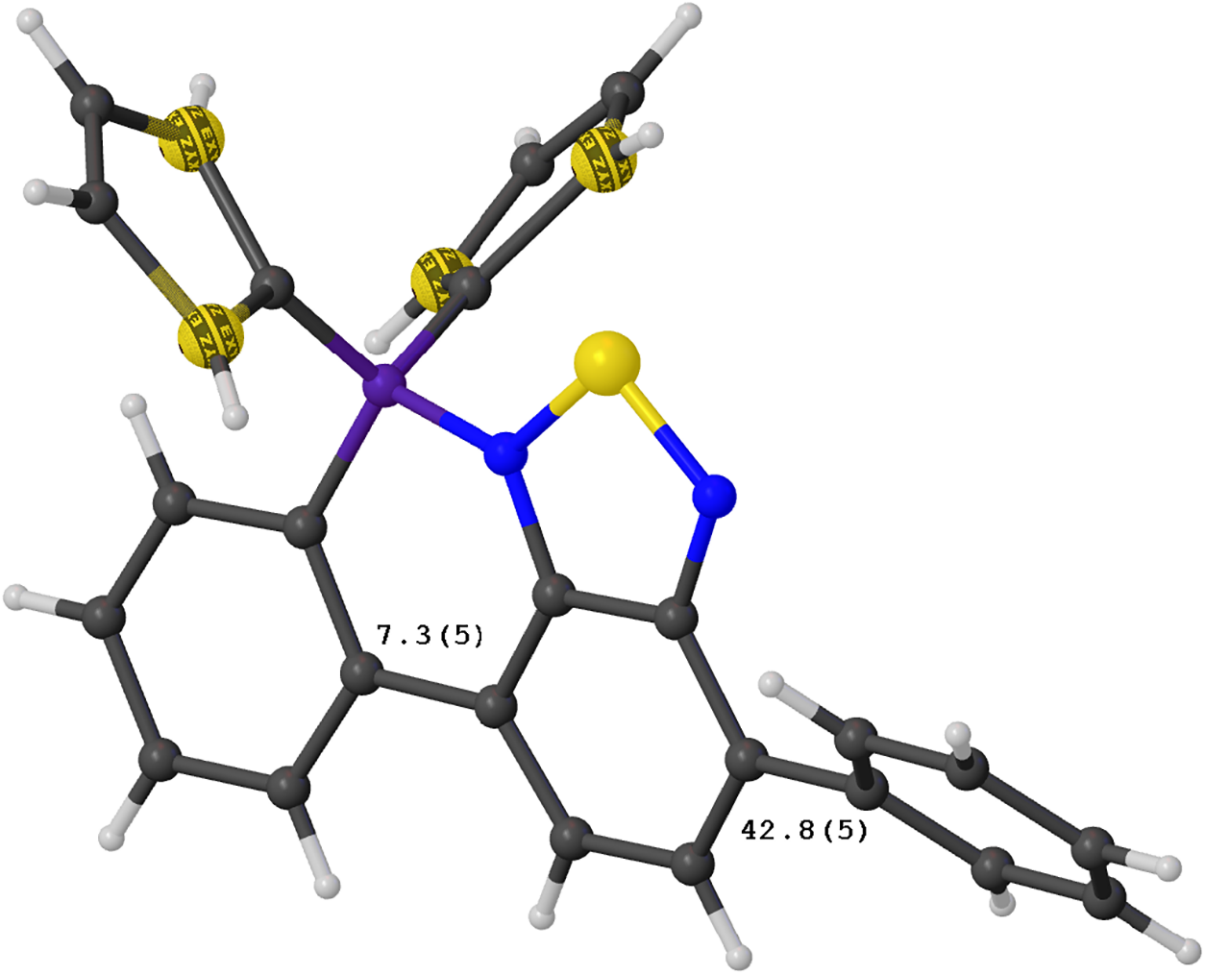
Single-crystal X-ray structure of **PhBTZ-BT**. Particular attention is focused on the difference in torsion angles of the **BTZ** block with respect to the two donor blocks: one fused (7.3°) and one not fused (42.8°).

This structural dependency of the bathochromic shift was further corroborated by the abrupt change in color and absorption back to the original parent **BTZ** of a nontransmetalated **ORG-PRC** upon contact with water or nonanhydrous solvents prior to transmetalation. In fact, in the wet solvent, the two chlorine atoms on the boron bridge are readily substituted into hydroxyl groups (hence forming boronic acid), and the dative bond from the Lewis basic nitrogen lone pair of the **BTZ** ring to the boron empty orbital is broken, changing the geometry of boron from four-coordinate to three-coordinate (Figure S63).^49,50^ The bathochromic shift of the absorption wavelength is comparable in magnitude to the emission shift, reaching a maximum λ_em_ with **TpBTZ-BP** at 700 nm (Table S1), while trends in both Δλ_abs_ and Δλ_em_ follow the same pattern. Interestingly, throughout the whole series of **ArBTZ-BAr′**, the emission intensity was heavily reduced compared to their relative parent **BTZ**s (leading to the decrease in the PLQY, φ_f_, *vide infra*). Thus, the emission spectrum was obtained with a concentrated solution (up to 1–5 mg in 3 mL), suggesting that none of the borylated **ORG-PRCs** fluoresce with great intensity with varying magnitudes and prefer nonradiative decay pathways, as corroborated by the low *T*_1_ value calculated computationally.

### Redox Potentials

To better assess the ability of the **BTZ**-derived **ORG-PRCs** in the target photoredox-catalyzed reaction, it was necessary to measure the ground-state reduction and oxidation potentials (*E*_red_ and *E*_ox_, respectively) by using cyclic voltammetry (*C–V*). Tests were run in DCM (with a redox potential window of ±2 V) rather than acetonitrile (with a redox potential window of ±3 V) due to the low solubility of the catalysts (analytes) in the latter. The electrolyte solution was made using tetrabutylammonium hexafluorophosphate. The most visible trend observed in the *C–V* of the **BTZ** library was the variation is *E*_red_ in virtue of the modification of the donor blocks. While it was already shown that electron-rich donor blocks (relative to **PhBTZ**) shift the *E*_red_ toward 0 V by Taylor et al.,^28^ the results of *C–V* were interesting nonetheless; *E*_red_ for all **ArBTZ-BAr′** was closer to 0 than **ArBTZ** with the exception of **PhBTZ-BP** (−1.51 V compared to −1.46 V of the parent **BTZ**) (Table S1). Although the variation among different **–BAr’** units with identical electron donor blocks was not entirely straightforward, a clear trend emerged: all **ArBTZ-BT** compounds exhibited lower magnitude of *E*_red_ values compared to the corresponding **ArBTZ-BP** compounds with equivalent donor blocks. The *S*_0_ (*v* = 0) → *S*_1_ (*v* = 0) energy difference (*E*_0,0_) could then be estimated by the intercept of the absorption and emission curves of each photocatalyst or by calculating half of the Stokes’ shift which is the difference between the peaks of the absorption and emission curves.^27^ Hence, by using the ground-state redox potentials in combination with *E*_0,0_, it was possible to predict the excited-state redox potentials (E*_red_ and E*_ox_), as described by Romero and Nicewicz (Table S1).^27^

From the calculation of E*_red_ and E*_ox_ of the **ORG-PRCs**, the first evident observation is that changing −**BAr′** ultimately does not substantially change the redox potentials in most cases. As a consequence of the *E*_red_ trend discussed above, E*_red_ was found to be always slightly greater for **ArBTZ-BT** with respect to their −**BP** counterparts. On the other hand, no obvious correlation was found regarding E*_ox_. The effect of the borylation itself on the **BTZ ORG-PRC** instead led to a lowering in E*_red_ in all cases but **tBuBTZ** examples, where E*_red_ was slightly increased upon ring fusion. Moreover, the magnitude of the shift in E*_red_ between the equivalent −**BAr′** group and their parent **BTZ** (e.g., comparing all different −**BT-**fused **BTZs** with their nonfused parent **BTZs**) varies through the different electron donors, starting from a maximum gain of +0.30 V for **tBuBTZ-BT** to a maximum decrease of −0.63 V for **PhBTZ-BP**. Regarding E*_ox_ instead, all ring fusions consisted in a shift toward 0 V, with the most prominent case being **TpBTZ-BP**, which shifted by 0.95 V, while the lowest was for **PhBTZ-BP** with 0.45 V.

Overall, the susceptibility of E*_red_ and E*_ox_ resulted in being pronounced for all **ArBTZ-BAr′**, both when compared to their respective parent **ArBTZs** and when compared between similar transmetalated −**BAr′** products. However, it was interesting to observe that changing −**BAr′** from **T** to **P** affected the *E*_red_ and *E*_ox_ only slightly, leading to the above-mentioned trend of all **ArBTZ-BT** > **ArBTZ-BP** for the *E*_red_. Thus, such synthetic modifications were not as impactful as ring closure itself and the modification of donor blocks. For this reason, only one example of furanyl **BTZ** (**PhBTZ-BF**) was made to scan for any peculiarity in optoelectronic properties.

### Photoluminescence Quantum Yield (PLQY, φ_f_)

The photoluminescence quantum yield (PLQY, φ_f_) can be defined by the fraction of photons emitted by the **ORG-PRC** over the amount absorbed, reported in %, and was measured using the procedural conventions by Jones et al.^50^ While the above-mentioned redox potentials are useful to understand the range of reactions achievable by the photocatalysts, the φ_f_ is a fundamental measurement to better understand their efficiency.

The most prominent observation in the series was the difference in the φ_f_ between each of the **ORG-PRCs** and their parents’ nonborylated versions. In fact, the φ_f_ was heavily reduced in all **ORG-PRCs** (up to a reduction by 89.9% upon borylation for **FluBTZ-BT**), with the **ORG-PRCs** of the lowest φ_f_ being **2NapBTZ-BP**, with only 0.3% of the light absorbed being measured in emission. The series spans from the lowest value of **2NapBTZ-BP** to the highest value of **PhBTZ-BT** with 56.3%, which still shows a significant decrease from the parent value of **PhBTZ** with 86.4% (Table S1). This impactful change in the φ_f_ can be explained by the energy band gap law, which states that the rate of radiationless transitions (such as internal conversion and intersystem crossing) increases with the decrease of the energy gap between the states.^51^ Computational calculations discussed below corroborate this, as the calculated *T*_1_ values were found to be too low to have a radiative decay pathway. This impactful change makes this modification potentially valuable also for applications beyond photocatalysis where nonradiative relaxation pathways are preferred, such as for photothermal therapy (PTT) for cancer treatment, already studied with other **BTZ**-based materials. Although, this is beyond the scope of this study, we kindly redirect the reader toward the literature on **BTZ** employed in these fields.^31,52–54^

### Computational Calculations

Electronic structure calculations were employed to investigate the absorption and emission of the borylated **BTZs**. The Gaussian 16 Revision C program package was used for all calculations.^55^ Ground-state geometries were obtained with B3LYP/cc-pVDZ, while energies involving excited states were computed at the CAM-B3LYP/cc-pVTZ level of theory, i.e., vertical absorptions, adiabatic excitation energies, and fluorescence energies. All calculations presented in the main text were carried out in the SMD solvent model parametrized for dichloromethane to mimic the solvent environment of the experimental measurements (Table S3). The calculations overall found good agreement with the experimental data. Moreover, for all **BTZs**, the T_1_ state was investigated as well, but was found to be too low in energy for phosphorescence to be the radiative decay pathway, hence corroborating the contribution to the lowering of the φ_f_ mentioned above upon borylation. The *S*_0_–*S*_1_ transition in all cases is described purely by a HOMO–LUMO, π–π* transition that transfers charge from the phenyl groups to the electron-accepting **BTZ** group, as shown in Figure 5 for **PhBTZ-BP**. Moreover, it is interesting to notice that neither the HOMO nor the LUMO present much character on the −**BAr′** bridge, corroborating the above-mentioned low participation of such a bridge modification to the photoactivity (Figure 5). Finally, the structures calculated align positively with the structures of crystals grown from dichloromethane, providing further validation of the computational method adapted.

**Figure 5:**
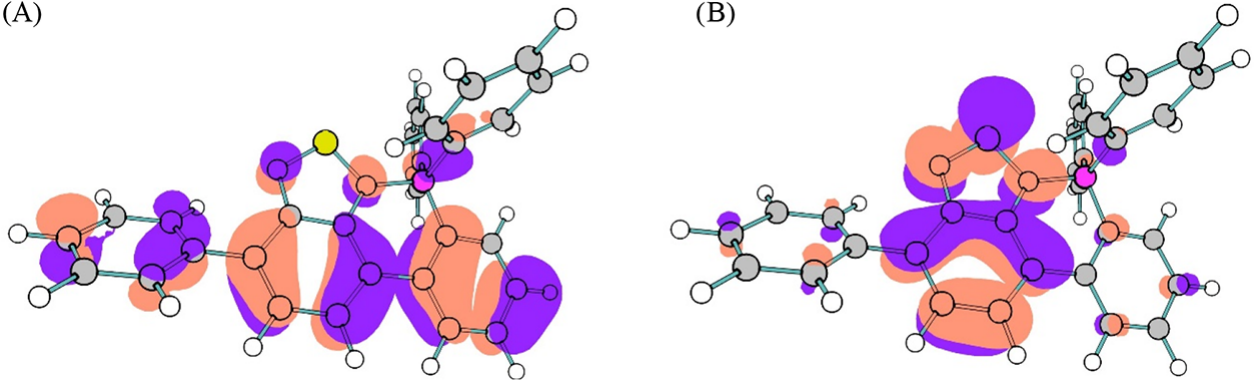
(A) HOMO and (B) LUMO orbitals of **PhBTZ-BP** that make up the *S*_1_ excitation.

### Batch C-4 Phosphorylation

The synthesis for the phosphorylation of quinoline derivatives was followed using the procedure from Cui et al.^10^ This was achieved using different quinoline derivatives, diaryl phosphine oxides, with sodium persulfate as a sacrificial oxidant and an **ORG-PRC** in DMSO (Table 1 and Figure 6). Given that the reaction has been extensively tested with an Ir-based **PRC**, exploring 9 different phosphines and 27 different quinoline derivatives, this work now focuses on using 8-fluoroquinoline (**Q**) and diphenylphosphine oxide (**DPPO**) rather than examining the full range of compounds. Compound **Q** was chosen among other quinoline derivatives for its availability and affordability and for the ^19^F NMR handle presented by the fluorine atom, which made the analysis more direct and reliable and not dependent on the weight yield but on direct NMR measurements.^56,57^ Furthermore, the choice focused on this quinoline derivative and **DPPO** due to the similarity of the obtained product with the reported array of antibacterial products in the cited patent by Qiu et al.^5^ Other derivatives have been used for completion of narration (e.g., lepidine and quinaldine), as summarized in the Supporting Information document (Tables S2 and 1).

**Figure 6:**
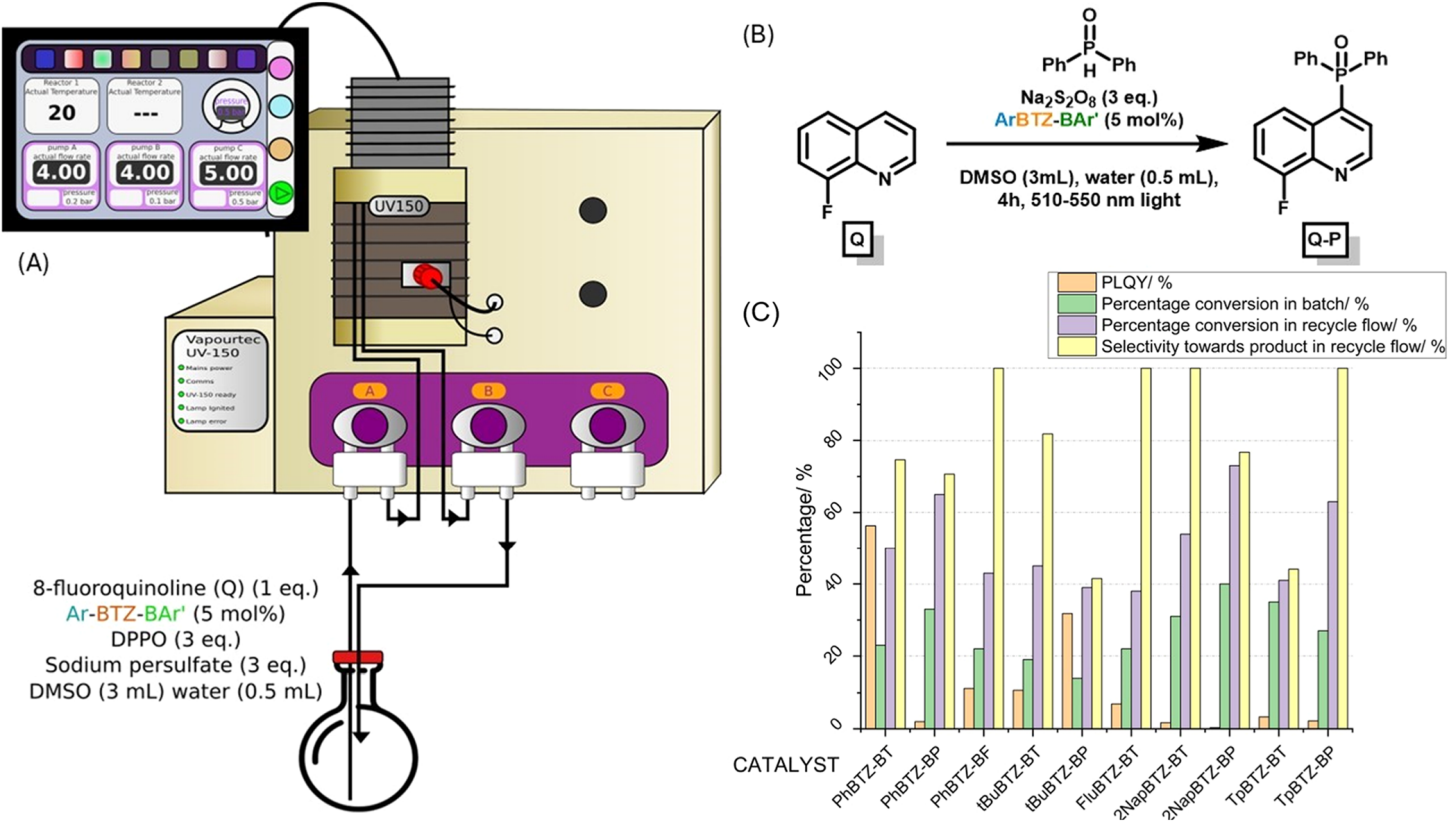
(A) Recycle flow machine schematics, with two pumps (4 mL/min) and one photoreactor. (B) Phosphorylation reaction scheme. (C) The respective photoluminescence quantum yield (PLQY, φ_f_, orange), conversion to the desired product **Q-P** in batch (green) and in recycle flow (purple) under optimal conditions, and selectivity toward **Q-P** in recycle flow.

**Table 1:**
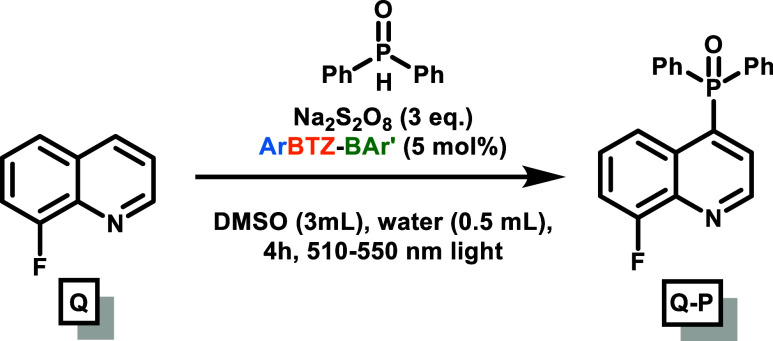
Summary of Results and Controls for the Phosphorylation of Quinoline Derivatives under Optimal Reaction Conditions^a^

entry	**ORG-PRC**	conversion to product **Q-P** (%)^b^	starting material left (%)^b^	time (h)	variation from standard conditions
1		9	89	4	no **ORG-PRC**
2	**PhBTZ-BT**	23	62	4	none
3	**PhBTZ-BP**	33	67	4	none
4	**PhBTZ-BF**	22	78	4	none
5	**tBuBTZ-BT**	19	81	4	none
6	**tBuBTZ-BP**	14	74	4	none
7	**2NapBTZ-BT**	31	69	4	none
8	**2NapBTZ-BP**	40	60	4	none
9	**FluBTZ-BT**	22	78	4	none
10	**TpBTZ-BT**	35	65	4	none
11	**TpBTZ-BP**	27	73	4	none
12		49	12	24	24 h, no **ORG-PRC**
13	**PhBTZ-BP**	70	0	24	24 h, **PhBTZ-BP**
14	**2NapBTZ-BP**	82	0	24	24 h, **2NapBTZ-BP**
15^c^	**2NapBTZ** ^c^	15	85	4	control **2NapBTZ**
16^c^	**TpBTZ** ^c^	17	83	4	control **TpBTZ**

a Standard conditions: 9-fluoroquinoline (Q) (0.2 mmol), diphenylphosphine oxide (0.6 mmol), oxidant (0.6 mmol), **ORG-PRC** (5% mol), DMSO (3 mL), deionized water (0.5 mL); reaction time: 4 h; T: room temperature (average measured 24 °C); 12 W, 510–520 nm LED.

b Conversion determined by ^19^F NMR for **Q** and ^1^H NMR for other quinoline derivatives.

c 420 nm, 12 W LED light used instead of 520 nm ones due to the higher absorption energy maximum of nonborylated **ORG-PRC**. The reader is kindly redirected to Table S2 for complete data in relation to the tests reported.

The reaction was first tested using quinaldine with **PhBTZ-BP** as the catalyst and a loading of 5 mol %, producing an 86% yield in 21 h. After observation of no conversion with lepidine (justified by position 4 being blocked by a methyl group), the tests with **Q** showed good yields compared to the control throughout the whole library. In fact, in 4 h of reaction time in batch under 530 nm light, the borylated **BTZs** proved to outperform the control test (no **ORG-PRC** 9%) with conversions spanning from a minimum of 14% for **tBuBTZ-BP** to a maximum of 40% for **2NapBTZ-BP**. Hence, ^19^F NMR conversions of batch reactions are presented in a decreasing order of magnitude: the obtained conversions in the 4 h span from 14 to 40%, with **2NapBTZ-BP** performing the best. (Table 1, Figure 6). Thus, a test in deuterated solvents (DMSO-d6, 3 mL; and D_2_O, 0.5 mL) was carried out prior to the optimization runs to verify the correct loading of **ORG-PRC** relative to **Q**. The results of these initial tests confirmed that the ratio between **Q** and **ORG-PRC** was indeed 20:1 by integration on ^1^H NMR before and after reaction and, hence, any residual solvent and vacuum grease residues (found in NMR spectra) did not affect the loading beyond ±0.3%. However, to verify that no decomposition of **ORG-PRC** or **Q** was occurring and that loading remained steady at 5% throughout the reaction time, a test run with an internal standard was performed (see entry 27, Table S2). The standard chosen was fluorobenzene (0.2 mmol, 18.6 μL). The test showed no loss in material during the reaction and correct loading (absolute) of 5% of **PhBTZ-BP** with respect to **Q**. The outcome was observed to be 35% conversion in 4 h, which is in line with the previous runs of the same **ORG-PRC** (33% in optimal conditions, entry 3, Table S2).

These conversions were overall a significant development not only compared to the control without **ORG-PRC** but also compared to the tests with parent **BTZ**s, which could never reach above 17% in 4 h (**TpBTZ**) in an air atmosphere, despite using a higher-energy 410 nm light (which produces greater conversion and side-product formation in blank runs without **ORG-PRC** present compared to green light). Hence, these batch results showed that the borylation modification of these **ORG-PRCs** is not only viable but also almost necessary for **BTZ**-based **ORG-PRCs** to even be comparable to the Ir-based counterpart used in the original study. After the optimization results were completed for each **ORG-PRC**, it was possible to observe an empirically determined trend between the **ORG-PRC** batch conversions and their respective φ_f_ (Figures 6 and S66). In fact, the lowest φ_f_ corresponded to the top-performing **ORG-PRCs** (e.g., **2NapBTZ-BP** with 40% conversion and 0.3% PLQY). Furthermore, to compare these **ORG-PRCs** more adequately with the literature examples (4CzIPN; Ir­(p-F-ppy)_3_; Ir­(ppy)_3_; [Ir­(df­(CF_3_)­ppy)_2_(4,4′-(OMe)_2_bpy]­BF_4_)), a basic cost analysis of the starting materials and solvents used for the production of **2NapBTZ-BP** (the best-performing example) was prepared (for full raw material analysis and reference, see the Supporting Information). It was calculated that the net cost of raw materials for laboratory synthesis in US dollars ($) to date was $61.69 g^–1^ (in GBP £46.05 g^–1^) ($0.31, £0.23 per 0.01 mmol use). While the cost of materials is not the only factor that is included in the calculation of direct manufacturing costs, this value is presented here in comparison to the much higher raw material costs of Ir,^12,15–18^ which lead to retail prices (to date) from Sigma-Aldrich (as the reference) of the above-mentioned photocatalysts spanning from $1908 g^–1^ (£1424 g^–1^) (Ir­(ppy)_3_) to $5010 g^–1^ (£3740 g^–1^) (4CzIPN). For comparison, Ir­(ppy)_3_ from other suppliers such as Ossila and TCI shows similar prices: $2679 and $2492 g^–1^ (£2000 and £1860 g^–1^), respectively. The authors wish to underline that the direct manufacturing cost expressed here is not a final retail price but rather an initial reflection of the costs of production to which the costs of waste treatment of the plant (WT), utilities (UT), and operating labor (OL) have to be applied before obtaining a full production cost. However, these other variables are considered to charge significantly lesser than the raw material cost when comparing the syntheses of Ir and **BTZ ORG-PRCs**, which differ by 1 order of magnitude.^12,15–18^

The reusability of the borylated **ORG-PRCs** was tested by recovering the catalyst after the reaction with sufficient purity via flash chromatography (ethyl acetate 100%, fraction 1). After recovery, the test resulted in a loss of 9% of its initial efficacy (30% conversion versus 33% in optimal conditions). This discrepancy is consistent with the error in the production of **Q-P**; however, it is also possible that partial recovery might have had an impact, as traces of **PhBTZ-BPh** could be found in fraction 2, mostly containing **DPPO**.

The time frame of 4 h was chosen also in light of the interesting difference in selectivity between some tested **ORG-PRCs**, which converged for all photocatalysts in 24 h. Thus, the selectivity of this reaction was observed to be good for almost all photocatalysts under 4 h. In fact, while there is no mention of side products forming with the iridium catalyst or any control test in the original study by Cui et al., it was observed that a C-2-phosphorylated side product (**2-Q-P**) formed prominently in control tests without **ORG-PRC** and with less efficient **ORG-PRCs** in the library (namely, **tBuBTZ-BP** and **PhBTZ-BT**) or with 410 nm LED blocks, sinking the overall **Q-P** conversion. Control tests in batch showed a ratio of 49:39 between the desired product **Q-P** and the **2-Q-P** side product in 24 h (55% selectivity of the product Q-P over total conversion). On the other hand, all **ORG-PRCs** showed a better selectivity, with the two producing the side products **tBuBTZ-BP** (56% selectivity) and **PhBTZ-BT** (60% selectivity). All other **ORG-PRCs** tested in the library did not show significant side-product formation within the 4 h time frame. However, after 24 h, all tests contained the side product (typically with 70% selectivity) when the reaction was run to completion.

Overall, it is important to note that a direct comparison with the literature study by Cui et al. is neither possible nor desired by the authors, as there are a few differences between their Ir-based study and the batch tests of this work; hence, the intention of this work is to be read as an effort to improve the sustainability and scalability of this reaction by designing new organic **BTZ ORG-PRCs**. The first experimental condition difference (in batch) that should be acknowledged is the **ORG-PRC** loading, which for Irppy_3_ was 1 mol % versus 5 mol % of our **BTZs**. It is noteworthy that the loading difference can be acknowledged in large-scale production by the huge difference in raw material prices and availability discussed above. Furthermore, the optimized condition included LED lamps of 12 W, 520 nm over a time frame of 4 h in an air atmosphere, compared to the higher-intensity 18 W 400 nm LED used in literature over a period of 24 h under argon. An inert argon atmosphere was also deemed useful for conversion, but not necessary, and was hence removed in batch experiments in an effort toward making this reaction greener and more scalable outside laboratory conditions. Finally, no bis­(pinacolato)­diboron (B_2_pin_2_) was used in our reaction conditions, as even if it was proven to increase the reaction rate, its mechanistic role was still unclear, and it was deemed more atom-economic to remove this aspect from this work. Therefore, to mitigate these differences, a further test was performed in batch (see entry 29, Table S2) under conditions as similar as possible to the above-mentioned literature examples. The conditions that were changed compared to the optimal ones (entry 13, Table S2) included, first, a lower loading (1%), matching the original study. The reaction was subsequently carried out in an inert atmosphere by cycling the vial with nitrogen gas and degassing the solvents for 15 min prior to the start of the reaction. The reaction was kept under a positive pressure of nitrogen throughout. Lastly, to match the photon flux in the original study (18 W) to the best of our capabilities, two 12 W lamps were used to irradiate the reaction, where one had half of the LEDs entirely covered to impede light from escaping. This emulated the conditions used in literature and led to a conversion over 24 h of 81% (with a trace amount of **Q** left and 84% selectivity) with **PhBTZ-BP**, thus obtaining better results than the optimized conditions mentioned above. This result can be attributed to the effect that a 50% increment in the power of the light source, a longer reaction time, and the inert atmosphere gives to the reaction (increasing the photon flux and lowering side-product formation drastically), despite the decrease of the loading.

In their study, Cui et al. extensively explored a series of 27 quinoline derivatives and 10 phosphines as the scope of their reaction. Considering this, the authors focused mostly on **Q** and **DPPO** as substrates as proof of concept for the use of the **BTZ ORG-PRCs**. Nonetheless, it was still deemed useful to scan through two different moieties to test the efficiency of a benchmark photocatalyst (**PhBTZ-BP**) under other conditions. Aside from **Q**, the selected quinoline derivatives were quinaldine (position C-2 blocked) and lepidine (position C-4 blocked). The tests were performed in batch with optimized conditions: quinoline derivative (0.2 mmol, 1 equiv), sodium persulfate (0.6 mmol, 3 equiv), **DPPO** (0.6 mmol, 3 equiv), **PhBTZ-BP** (5 mol %), and DMSO (3 mL) in air with the appropriate 12 W, 520 nm LED blocks for 24 h. The lepidine example showed no conversion at all. This can be explained by the fact that the C-4 position of the heteroaromatic ring is already methylated; hence, it was interesting to observe no side-product formation in other positions of the ring. In contrast, tests involving quinaldine presented the formation of the product **Quin-P**, albeit with slower reaction rates, obtaining 13 and 50% conversion over 4 and 24 h, respectively. When compared to the control with no **ORG-PRC**, we also observe a slower reaction rate for the noncatalyzed pathway for **Quin-P** (5% at 4 h and 18% at 24 h), thus promoting a 2.7-fold increment in the reaction rate when the borylated **ORG-PRC** is employed.

### Recycle Flow Phosphorylation

The next step in this study was to explore this phosphorylation reaction in recycle flow for the above-mentioned benefit this system inherently offers to any photocatalytic system. Initially, the same conditions employed in batch were maintained to directly compare the efficiency of this new system. The recycle flow reactor used was the Vavaportec Easy-Photochem system, equipped with a UV-150 photoreactor with 525 nm LEDs and a 10 mL reactor coil. The temperature was regulated at 25 °C and the system was purged with N_2_ gas throughout all recycle flow experiments (Figure 6). The recycle flow setup was constructed using a 1 mm diameter fluorinated ethylene propylene (FEP) transparent tubing to connect all of the components, starting from the reaction flask. Initially, a peristaltic pump (pump A, 4 mL/min) pushed the reaction mixture into a reactor coil (10 mL) irradiated by a 525 nm, 12 W lamp. After the reactor coil, another pump (pump B, 4 mL/min) was used to pull reagents and prevent clogging of any undissolved solid oxidant, flowing the reaction mixture again into the initial flask and hence closing the reaction cycle. Initial tests performed using **PhBTZ-BP** showed an impressive improvement in conversion during the 4 h reaction time frame, with a total conversion of 92%, where 65% was the desired product **Q-P** and 27% was the side product **2-Q-P** (71% selectivity). To obtain similar results in batch, it was necessary to perform the reaction over 18 h, accelerating the reaction rate by a factor of 4.5. The higher rate of conversion was attributed to the greater efficiency of irradiation of the thin tubing compared to the glass vial normally used for batching (Table S4).

A control experiment in the absence of **ORG-PRC** did not show the same 4.5-fold improvement in efficiency (13% in recycle flow vs 9% in batch). This suggests that there are both radiative and nonradiative routes to the product, corroborating the mechanism proposed by Cui et al. which included two routes, respectively, with and without **ORG-PRC** or light, to obtain the sulfate dianion (SO_4_^2–^), which then proceeds to deprotonate **DPPO** (−H^+^) and initiate the radical reaction. This comes to an advantage as it renders the catalyst more impactful, as the gap between the control and the **ORG-PRC** runs rises in recycle flow. To further prove the mechanism, a radical trapping experiment using TEMPO was performed with the TEMPO-**DPPO** adduct observed by mass spectrometry and no **Q-P** yield observed. Thus, the tests in optimal conditions were run for each **ORG-PRC**, obtaining the best result in 4 h with **2NapBTZ-BP** (91% total conversion, 73% **Q-P** conversion, 80% selectivity), which was also the best-performing **ORG-PRC** in batch (40% **Q-P** conversion in 4 h, Table S4). The selectivity observed in recycle flow in 4 h was also comparable to that found in 24 h batch experiments. In recycle flow, it was possible to observe a moderate increase in selectivity for the desired product to 3:1 for the same **ORG-PRC**, increasing from 66 to 75% selectivity under the same conditions. Hence, this suggests the possibility that the side-product formation is more temperature-dependent than the desired product, as heat dispersion is more efficient in recycle flow compared to batch, and that the temperature never reached above 25 °C. In contrast, the temperature in batch was measured at the surface of the glassware using an IR thermometer, reaching up to 28 °C during overnight tests.

### Reaction Monitoring and Productivity

The use of recycle flow chemistry for this reaction was not solely chosen for the reaction yield enhancement due to the better irradiation but also related directly to the reaction monitoring options discussed above for other flow systems.^37–39^

Considering the recycle flow results of the previous section, we then focused on the in-line analysis of the reaction mixture during the phosphorylation of **Q** using UV–vis absorption spectroscopy with the PASCO spectrometer and a large-scale reaction test (10 times the scale; 2 mmol of **Q**, 1 equiv). The larger scale was tested to both ensure that the efficiency would not drop significantly (as it would in batch at the same scale) and to fill all reactor tubing volumes, preventing false data caused by N_2_ gas bubbles in the measurements (Figure 7).

**Figure 7:**
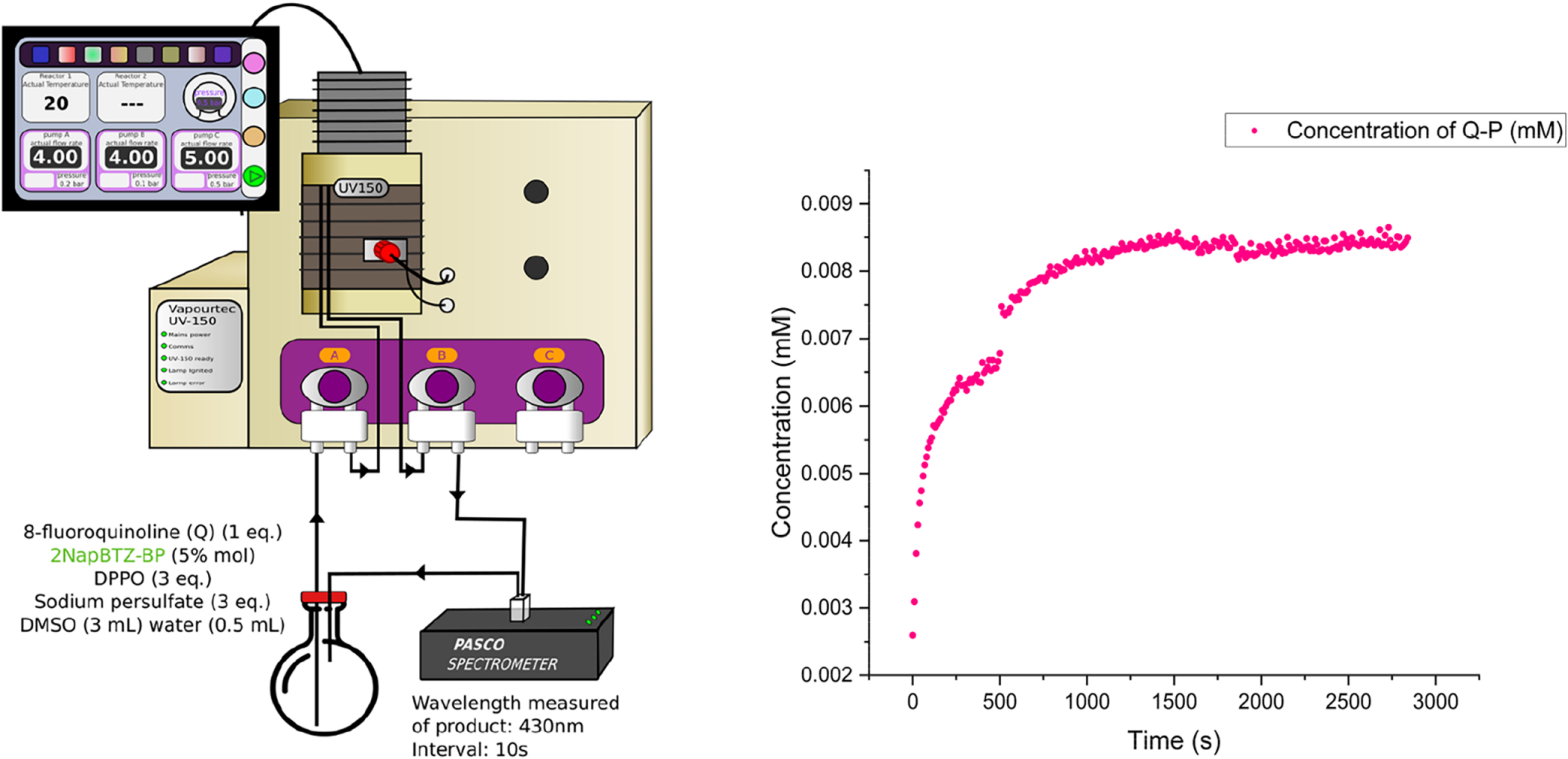
Reaction profile for the phosphorylation of 8-fluoroquinoline in optimal conditions in recycle flow using the **2NapBTZ-BP** photocatalyst. The absorption wavelength selected was 430 nm, monitored at 10 s intervals over a period of 50 min using an inflow/outflow quartz cuvette with a PASCO scientific fluorescence instrument. The concentration of Q-P was measured by UV–vis absorption spectroscopy against NMR data at known concentrations.

The measurement was set to observe the profile of the absorbance in time at 430 nm (maximum absorption measured for **Q-P**) with an interval of 10 s over a period of 40 min. This allowed the observation of a nonlinear reaction profile to produce **Q-P**. The absorbance value (in arbitrary units) was then converted to the concentration (mM) by knowing the initial concentration of starting materials and by measuring the integration of the **Q-P** peak (singlet, δF −123.3 ppm) against the starting material **Q** (singlet, δF −126.7 ppm) by ^19^F NMR spectroscopy. Additionally, the crude starting solution underwent initial blanking to prevent any false measurements from the **ORG-PRC** peak. Although it does not absorb at 430 nm, this step was considered beneficial to ensure the accuracy of the measurement at that wavelength. This measurement showed 21% conversion in 30 min of reaction and 63% over 4 h, thus losing 10% conversion over 4 h of reaction time (with respect to standard-scale optimized conditions), albeit keeping the same reactor volume and increasing the reaction mixture volume 10-fold. The plateau of the reaction profile could also be due to the nature of the scale-up, as with a 10-fold reaction volume increment (35 mL), this is now greater than the reactor coil volume (10 mL), leading to the fact that over time, each cycle will contain on average less and less **Q** starting material molecules and more **Q-P** or **2-Q-P** that are flown in the reactor coil, thus delaying the tail of the reaction.

In order to better determine the scalability of this system, a series of common calculations were carried out for the residence time (section 9, ESI), productivity (in mass and mol), and space-time yields (STYs) for a 10-fold scaled-up recycle flow run (entry 13, Table S4).^58^ The residence time is determined as the time of each full cycle through the recycle flow system of the reaction solution (35 mL) that is spent inside the reactor coil (10 mL) over the duration of the reaction (thus multiplying this effective time by the number of cycles over the 4 h of reaction time). The residence time of the reactor coil length and the time to complete a full cycle of the recycle flow system (at the flow rate of 4 mL min^–1^) had to be obtained using eqs S1 and S2, leading to 2.5 and 8.75 min, respectively. The fraction of time spent in the reactor coil during each cycle is then the fraction of the two or, by simplification of the flow rate from eqs S1 and S2, the ratio between the reactor coil volume and the reaction volume (eq S3). This results in 0.29, which suggests that only 29% of the whole reaction time in this setup is spent in the reactor, where irradiation occurs. This value decreases as we increase the volume (and scale) of the reaction without increasing the reactor coil length. The number of cycles can also be obtained easily by computing the fraction of the full reaction time (4 h) and the time of a single cycle from eq S2 (eq S4), resulting in 27.4 cycles. Finally, we can obtain the residence time as the product of the time of a single cycle, the active path during the cycle, and the number of cycles, leading to 69.6 min or 1.16 h (eq S5). Notably, and as this is obtained from eq S3, the above-mentioned decrease to this value will occur to the residence time if the reaction volume scales up with respect to that of the reactor coil.

The productivity, instead, can be defined as the ratio between the measured conversions of **Q-P** over the full reaction time. This can be calculated directly from measured values of conversion by ^19^F NMR (knowing no loss in mass or degradation of **Q** or **Q-P** was observed with an internal standard test) over the total reaction time of 4 h, while keeping in mind that the conversion was obtained within a system that leads to only 1.16 h of the effective residence time. Thus, the productivity was found to be 0.3 mmol h^–1^ or 104 mg h^–1^ (eq S6).

Similarly, the STY can be used as a clearer instrument to compare different reactors as it also includes the volume of reactor. The STY was obtained by the fraction of the amount of the product over the reactor coil volume and full reaction time (eq S7). Hence, the obtained STY was 0.024 mmol h^–1^ mL^–1^ or 9 mg h^–1^ mL^–1^, as the isolated yield of the reaction was 336 mg (0.97 mmol). Finally, the STY can be also defined using strictly the residence time of the reaction rather than the “gross“ full time it was carried out for. Following the same calculations as eq S7, we expectedly obtained the value for the “net” STY­(res) of 0.084 mmol h^–1^ mL^–1^ or 29 mg h^–1^ mL^–1^.

The scale of the reaction was further increased to 40-fold (8 mmol, 1.18 g of **Q**, entry 14, Table S4) to test any size-dependent dropout in the output of the system. The reaction was carried out for 4 h at 4 mL min^–1^ with **PhBTZ-BP** as the **ORG-PRC**, where all quantities but the size of reactor coil were increased by a factor of 40. The test produced two interesting observations. First, with the higher size, all of the reagents dissolved in the solvents homogeneously, while in the small scale, it was observed that the residual sacrificial oxidant remained undissolved despite the use of 0.5 mL of water and thus could be pumped through the system as a slurry. Second, the conversion in 4 h remained almost unchanged upon scale-up, with 68% **Q-P** produced in a 40-fold scale against 65% in the small scale (entry 14 versus entry 3, Table S4). The former result still lies within the 5% error observed in repeated experiments; thus, it comes with no surprise that the conversion is marginally higher. Instead, what was interesting to observe is that upon scaling up to the gram scale, the conversion was not hampered as expected, leading to a productivity of 1.36 mmol h^–1^ and a STY­(res) of 1.6 mmol h^–1^mL^–1^ (eqs S9 and S10). The authors attribute this to two factors (ultimately differentiating the two scales of conditions, favoring the scale-up): the higher solubility of the sacrificial oxidant, now homogeneously available, and the absence of air bubbles in the system (*vide infra*). Thus, the results of the 40-fold scale-up suggest that the scalability to gram-scale production is viable.

Finally, a factor to be taken into account for the comparison between the standard scale (0.2 mmol of **Q**) and scaled-up (2 mmol of **Q**) reactions is that in the latter, the reactor volume is smaller than that of the reaction, leading to the absence of gas bubbles in the line (as the system is now constantly filled by the reaction mixture). This suggests that side effects on light absorption observed for reactor coils with gas–liquid interfaces are absent upon scale-up.^59^ Such effects recently studied by Nol et al. showcase the impact of the reaction solution on light absorption (in coil-type photoreactors) when gas and liquid phases are present in flow photoreactions. The effect was reported to be dependent on the surface area of contact between the solution media and the gas bubbles, ultimately dampening the reaction effectiveness. In the present setup, under optimized conditions, the 3.5 mL solution was observed to travel through the coil in one homogeneous block, rather than a series of liquid bubbles separated by gas, limiting the gas–liquid interface area and thus minimizing yet not denying the absorption dampening effect. This effect must be taken into account when thinking about the overall increment in efficiency in recycle flow compared to batch, as a fair comparison between the two compelled the authors to use the same reaction mixture and conditions between the two systems (3.5 mL volume, using a 10 mL reactor coil as the only volume of coil available). Hence, while this effect is present equally among all recycle flow tests (thus equally affecting all recycle flow tests), this becomes relevant when scaling up. Overall, while the large-scale tests present smaller residence times compared to 0.2 mmol tests, they are not affected by the slight loss in overall reactivity discussed above, and so the difference between standard-scale and scaled-up reactions in recycle flow may be slightly smaller than the direct comparison of the two tests while still showing a drop in conversion upon scaling up 10-fold.

### Sequential Phosphorylation and Minisci Coupling

Taking the synthesis forward, we decided to use the advantages offered by the recycle flow setup to produce a more complex quinoline derivative by performing a Minisci-type radical reaction in sequence to the phosphorylation to form a C–C bond in position 2 using cyclohexane carboxylic acid as the source of an alkyl radical.^28,60^ This tandem approach would present an alternative room-temperature route to the antibacterial drugs developed by Qiu et al. at 75 °C in a similar reaction time (Figure 1B) as well as drug analogues of the other cited studies on phosphorylated quinoline derivatives used in antiviral and cancer treatment applications discussed above.^1,4^ Initially, the test was performed in batch, where the parent benchmark **BTZ** (**PhBTZ**) managed to produce the desired (2-cyclohexyl-8-fluoroquinolin-4-yl)­diphenylphosphine oxide (**Q-P-Cy**) with a yield of 16% in 4 h and 45% in 24 h with a 410 nm light source. The same conditions were tested for the best-performing **ORG-PRC** of the library (**2NapBTZ-BP**). However, to obtain a similar yield (47%) at 25 °C, 72 h of reaction time in recycle flow was required when using 525 nm LEDs. After scanning through different conditions for optimization, this gap in efficiency for the Minisci reaction was attributed to the differences in excited-state redox potentials between parent **BTZs** and borylated **BTZs**. In fact, the oxidation potential of parent **BTZs** was consistently higher in magnitude than that of the borylated analogues (as discussed in the redox potential section, Table S1). This suggests that although the borylated **BTZs** are more efficient in the initial SET photoredox process (remaining well within the required potential of −0.72 V), the second Minisci-like reaction likely falls outside this range. As a result, the **ORG-PRC** may be unable to transfer electrons to the substrate with optimal efficiency.^10^

Thus, although possible with **2NapBTZ-BP**, to obtain the desired target drug molecule **Q-P-Cy** in a more efficient way, the sequential automated reaction was performed in recycle flow with two **ORG-PRCs**, starting with the same initial setup for the phosphorylation as the first step (2 pumps, 5 mol % loading of **2NapBTZ-BP**, 550 nm UV light for 4 h (see Figure 8)).

**Figure 8:**
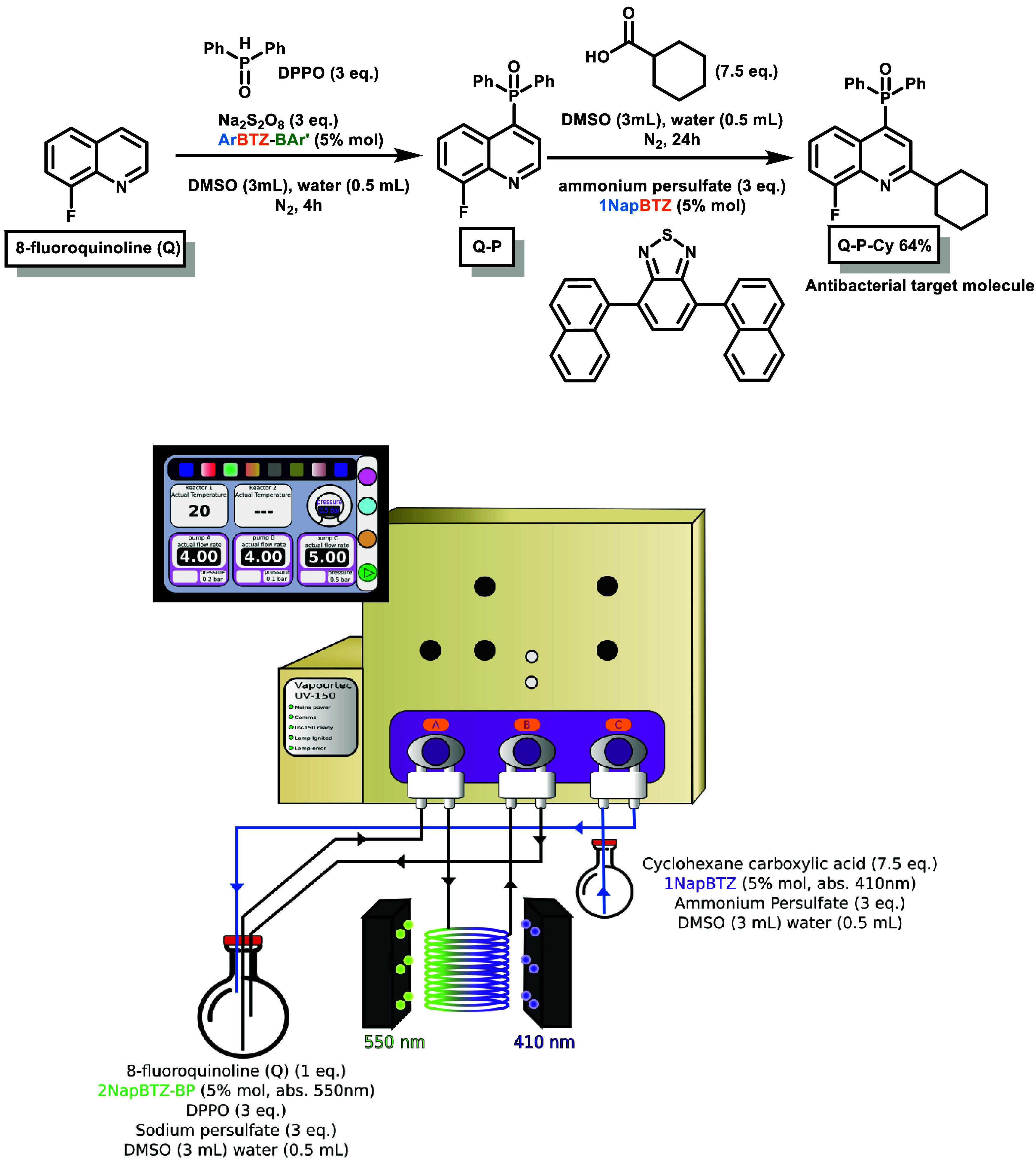
Tandem automated C-4 phosphorylation and Minisci reaction scheme (top) with the recycle flow schematic diagram (bottom). Note that pump C (blue line) and the second LED block (410 nm) were set up to automatically initiate only after 4 h to ensure that all Q starting material was consumed prior to the Minisci step.

Subsequently, a third pump was programmed to automatically inject a solution containing cyclohexane carboxylic acid, ammonium persulfate, and 5 mol % **1NapBTZ** (the best-performing photocatalyst in the library developed by Taylor et al.),^28^ and a second external LED module (410 nm) was lit automatically at the same time to accommodate the optimal absorption wavelength of **1NapBTZ**. In doing so, the second Minisci-type reaction was initialized without intermediate purification through autonomous and subsequent addition of the required starting materials, leading to complete consumption of the starting material after 24 h and resulting in a total conversion to the desired product of 64%. This outcome shows the capabilities of both borylated and nonborylated **BTZ** photocatalysts when employed in a more complex reaction setup. Flow programmed pumps and LED timers granted automation and streamlining of this process, leading to the desired **Q-P-Cy** antibacterial drug in the same reported reaction time of 24 h,^5,10^ but without heat and by initializing from simpler and cheaper starting materials rather than quinolines with presubstituted position 2 or with extra synthetic steps.

## Conclusions

In conclusion, we have reported the synthesis and optoelectronic characterization of 10 **BTZ**-based **ORG-PRCs**, obtained with an inexpensive two-step, one-pot borylation and transmetalation process. These modified **BTZ ORG-PRCs** revealed, among other features, an important bathochromic shift up to 142 nm while also heavily reducing the φ_f_ across the whole series when compared to their nonmodified counterparts (with an absolute minimum φ_f_ of 0.3% for **2NapBTZ-BP**). The design of these **ORG-PRCs** was based around an increasingly relevant photoreaction such as the C-4 phosphorylation of quinoline derivatives, as the number of phosphine- and phosphate-containing drugs is rapidly increasing alongside the already vast number of quinoline derivative moieties in biological applications.^1–7,10^ This transformation was tested in batch, obtaining a valuable increase in efficiency up to a 40% conversion in 4 h while performing significantly better in recycle flow, as demonstrated by up to 73% conversion under analogous conditions. The recycle flow reaction system was then pushed further to obtain a proven antibacterial (*Escherichia coli* and *Staphylococcus aureus*)^5^ target moiety by performing a tandem Minisci reaction at the C-2 position following the first step in an automated 4 + 24 h process (Figure 8),^5^ obtaining 64% of total conversion to **Q-P-Cy** without requiring costly purification of the intermediate **Q-P**.

## Supplementary Material



## Data Availability

The data underlying this study are available in the published article and its Supporting Information.

## Notes

Experimental procedures, UV–visible absorption and emission data, NMR spectra (^1^H NMR, ^11^B NMR, ^13^C NMR, ^19^F NMR), cyclic voltammetry plots, X-ray crystal structures deposited as 2447623–2447628, full tables and procedures of photocatalysis tests in batch and recycle flow, computational studies of the photocatalysts, productivity calculations, and cost analysis. All crystallographic data have been deposited at the CCDC under 2447623–2447628, as specified in the Electronic Supporting Information document (PDF)
