# Supplementary material for: Green-Light Photocatalysis: Borylated Benzo[c][1,2,5]thiadiazole (BTZ) Enables Phosphorylation of Quinoline Derivatives
Source: J Org Chem. 2025 Sep 29;90(40):14081–93. doi: 10.1021/acs.joc.5c01367 (PMC12519470; doi:10.1021/acs.joc.5c01367)
Supplement: Supplementary file 1 [file jo5c01367_si_001.pdf]

Green-Light Photocatalysis: Borylated Benzo[c][1,2,5]thiadiazole (BTZ) Enables Phosphorylation of Quino-line Derivatives

Authors: Leonardo Amicosante,[a] Dominic Taylor,[a] Luca Craciunescu,[a] Andrew W. Prentice,[a] Adilet Zhakeyev,[a] Bence Szabó,[a] Georgina M. Rosair,[a] Martin J. Paterson,[a] Scott J. Dalgarno,*[a] Filipe Vilela*[a]

[a]Institute of Chemical Sciences, School of Engineering and Physical Sciences, Heriot-Watt University, Edinburgh, EH14 4AS, UK.

All commercially available compounds were used as received and purchased from Sigma-Aldrich, Fluorochem, Apollo Scientific or Fischer Scientific. Where not stated otherwise, the reactions were executed at room temperature, at atmospheric pressure and in atmospheric oxygen presence. NMR spectra of synthetic products were recorded using a Bruker AVIII 400 MHz spectrometer using the residual solvent peak as an internal reference unless stated otherwise. All the NMR experiments were conducted in $CDCl_3$ at 298 K and atmospheric pressure and conditions, unless specified otherwise. UV-Visible absorption spectra for the synthesised photocatalysts were obtained using a Perkin-Elmer Lambda 35 spectrometer in chloroform solution in quartz cuvettes with a path length of 1 cm. Emission spectra in the visible region were recorded using a Perkin Elmer LS 55 fluorescence spectrometer in chloroform solution using quartz cuvettes with a path length of 1 cm. Cyclic voltammetry (CV) measurements were performed on an Iviumstat instrument; with glassy carbon working electrode, platinum wire counter electrode and CHI150 saturated calomel electrode (SCE, saturated KCl solution). Tests were run in DCM (with a redox potential window of +/-2 V). The electrolyte solution was made using tetrabutylammonium hexafluorophosphate. The photoluminescence quantum yields were measured following the conventions and considerations from Jones *et al.*[1] FLS920 spectrofluorometer (Edinburgh Instruments Ltd) equipped with an extended red-sensitive photon multiplier detector (Hamamatsu, R2658P) and an integrating sphere with a 102 mm inner diameter (Yobin Yvon) were used for all PLQY measurements. For the excitation, a 450 W Xenon lamp (Edinburgh Instruments Ltd, Xe2) was employed. The samples consisted of diluted dyes in $CHCl_3$, typically at 20 µM. The sample was contained in a square quartz cuvette with10 mm light path and placed in the centre of the integrating sphere. The reference sample, or blank, was pure $CHCl_3$. The excitation wavelengths were set to the peak absorption of each sample with a bandwidth set to 10 nm. The excitation and emission regions were typically measured with a 1 nm step size. The associated measurement error is 0.2%. All HRMS (ESI-MS) spectra of the synthesised photocatalysts were measured using time-of flight mass spectrometry using a Waters Acquity UPLC-Xevo G2 QTof with the electrospray ionisation source in positive mode.

Table of contents

Table S1 .... S-3
1. Experimental procedures: S-4
2. UV-Visible Absorption and Emission spectra S-11
3. NMR Spectra S-21
4. Cyclic Voltammetry S-49
5. X-ray Crystallographic data S-59
6. Photocatalysed Phosphorylation in batch S-66
Table S2 .... S-68
7. Computational calculations (Table S3) S-70
8. Photocatalysed Phosphorylation in flow S-75
Table S4 .... S-78
9. Productivity and Space time yield calculations S-79
10. Raw Materials Cost Analysis S-80
11. References S-82

Table S1 Summary of optoelectronic properties of the **BTZ** library. Notice the colour of the transmetallated aryl groups has been inserted for clarity (purple for thiophenyl groups, blue for phenyl groups, red for furan groups). All measurements were taken in $CHCl_3$ unless specified. **(a)** Value calculated according to procedure explicated by Romero *et al.*[2] **(b)** Compound decomposed onto the glass-carbon surface during sweep. **(c)** Absolute PLQY. **(d)** Difference between ArBTZ-BAr' and its respective parent ArBTZ with same donor block. **(e)** $E^{(p/2)}$ recorded instead for irreversible oxidation/reduction processes, according to procedure explicated by Espinoza *et al.*[3] and Romero *et al.*[4]

Photocatalyst	Absorption wavelength (nm)	Emission wavelength (nm)	Stokes' shift (nm)	$\Delta\lambda_{abs}$ (nm)[d]	$\Delta\lambda_{emis}$ (nm)[d]	E_{ox} (V)[e]	E_{red} (V)[e]	$E_{0,0}$ (eV)[a]	E^*_{ox} (V)[a]	E^*_{red} (V)[a]	PLQY/%[c]
**Ph-BTZ**	388	485	97	-	-	1.64	-1.46	2.84	-1.2	1.38	86.4
**Ph**-BTZ-**BT**	502	606	104	114	121	1.52	-1.40	2.23	-0.71	0.83	56.3
**Ph**-BTZ-**BP**	498	600	102	110	115	1.51	-1.51	2.26	-0.75	0.75	2.0
**Ph**-BTZ-**BF**	497	611	114	109	126	1.68	-1.45	2.23	-0.55	0.78	11.2
**tBu-BTZ**	397	505	108	-	-	1.61	-1.58	2.75	-1.14	1.17	76.2
**tBu**-BTZ-**BT**	524	630	106	127	125	1.68	-0.68	2.15	-0.47	1.47	10.7
**tBu**-BTZ-**BP**	521	628	107	124	123	1.45	-0.95	2.15	-0.70	1.2	31.8
**2Nap-BTZ**	406	510	104	-	-	-[b]	-1.35	2.7	-[b]	1.35	66.2
**2Nap**-BTZ-**BT**	540	645	105	134	135	-[b]	-0.55	2.09	-[b]	1.54	1.7
**2Nap**-BTZ-**BP**	543	643	100	137	133	-[b]	-0.73	2.09	-[b]	1.36	0.3
**Flu-BTZ**	424	527	103	-	-	1.3	-1.51	2.60	-1.30	1.09	96.8
**Flu**-BTZ-**BT**	566	675	109	142	148	1.33	-0.85	2.00	-0.67	1.15	6.9
**Tp-BTZ**	450	547	95	-	-	1.05	-1.39	2.49	-1.44	1.1	88.1
**Tp**-BTZ-**BT**	578	666	81	128	119	1.35	-1.16	2.00	-0.65	0.84	3.3
**Tp**-BTZ-**BP**	586	700	121	136	153	1.42	-1.17	1.91	-0.49	0.74	2.2

1. Experimental procedures:

Bromination of Benzo[c][1,2,5]thiadiazole (Br_2BTZ)

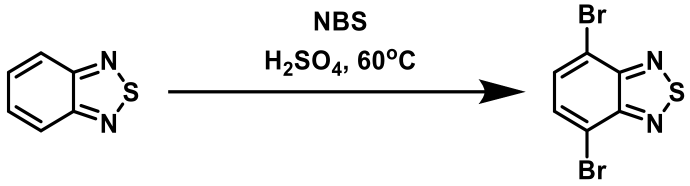

Benzo[*c*][1,2,5]thiadiazole (10g, 73.6 mmol, 1 equiv.) and *N*-bromosuccinimide (NBS) (26.34 g, 147.2 mmol, 2 equiv.) were put into a two-necked round bottomed flask. Concentrated sulfuric acid (100 mL) was added and the reaction mixture heated to 60 °C for 5 hours. An orange liquid and a yellow solid were formed. The solution was then cooled down to room temperature and poured into an ice water mixture. A white powder precipitated that was filtered by gravity, washed with copious amounts of deionised water, and dried under vacuum to yield white powder. The product was then recrystallised from chloroform (16.215 g, 75% yield).

^{1}H NMR data ($CDCl_3$, 300 MHz, 25.0 °C) δH 7.73 (s, 2H).

Full characterisation in literature procedures from Taylor *et al.*[5]

General Suzuki-Miyaura cross coupling procedure (4,7-diarylBTZ)

All 4,7-diarylBTZ were synthesised *via* Suzuki-Miyaura cross-coupling as follows, unless specified otherwise. Procedure followed from literature Taylor *et al.*[5]

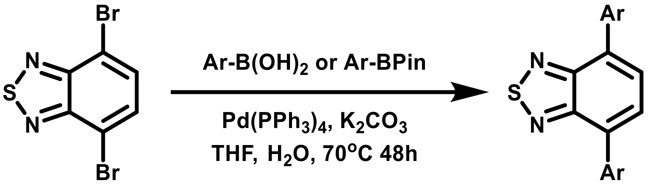

4,7-Dibromobenzothiadiazole (Br_2BTZ) (1 equiv.), aryl-boronic acid/boronic ester (2.5 equiv.), and K_2CO_3 (2 equiv.) were mixed in an oven-dry two-necked round bottomed flask and repeatedly evacuated and back-filled with N_2 gas twice under Schlenk line. $Pd(PPh_3)_4$ (5 mol%) was then added, and the flask sealed and cycled another three times. THF (40 mL) and water (5 mL) were degassed for 10 minutes and then added to the flask. The resulting mixture was heated with an aluminium heating block up to 70 °C under N_2 for 2 days. Hence, it was cooled down to room temperature and washed three times with water (20 mL x3) and DCM (20 mL x3), and organic layer collected and dried over Na_2SO_4. DCM was then removed by vacuum and product was recrystallised from hot methylated spirits.

4,7-Diphenylbenzo[c][1,2,5]thiadiazole (PhBTZ)

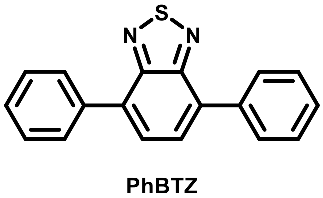

PhBTZ

Synthetic procedure followed as above, *via* Suzuki-Miyaura cross coupling using Br_2BTZ (294 mg, 1.0 mmol), Phenylboronic acid (305 mg, 2.5 mmol) and K_2CO_3 (276 mg, 2.0 mmol). The product was purified by recrystallisation from hot methylated spirits resulting in bright yellow needle-crystals (181 mg, 63% yield). **^{1}H NMR** ($CDCl_3$, 300 MHz, 25.0 °C) δH 7.97 (m, 4 H), 7.80 (s, 2 H), 7.59 (m, 4H), 7.47 (m, 2 H). **^{13}C NMR** {1H} ($CDCl_3$, 75.5 MHz, 25.0 °C) δC 154.1 (C), 137.5 (C), 133.4(C), 129.3 (CH), 128.7 (CH), 128.4 (CH), 128.2 (CH).

Full characterisation in literature procedures from Taylor *et al.*[5]

4,7-Bis(9,9-dioctyl-9H-fluoren-2-yl)benzo[c][1,2,5]thiadiazole (FluBTZ)

Synthetic procedure followed as above, *via* Suzuki-Miyaura cross coupling using Br_2BTZ (141 mg, 0.5 mmol), 9,9-dioctylfluorene-2-boronic acid (645 mg, 1.25 mmol) and K_2CO_3 (140 mg, 1.0 mmol). The product was purified by flash chromatography (Hexane/ethyl acetate 95:5) obtaining a bright yellow powder (475 mg, 52% yield). **^{1}H NMR** ($CDCl_3$, 300 MHz, 25.0 °C) δH 8.05 (d, 2 H), 7.98 (s, 2 H), 7.89 (d, 4H), 7.80 (m, 2 H), 7.39 (m, 6), 2.07 (m, 8 H), 1.12 (m, 48 H), 0.82 (t, 12 H).

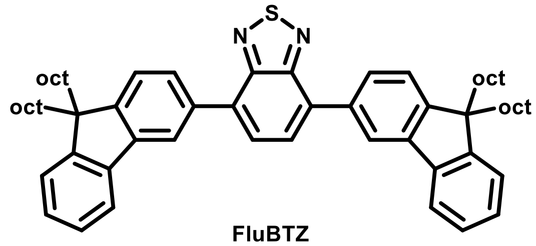

FluBTZ

Full characterisation in literature procedures from Taylor *et al.*[5]

4,7-Di(naphtalen-2-yl)benzo[c][1,2,5]thiadiazole (2NapBTZ)

Synthetic procedure followed as above, *via* Suzuki-Miyaura cross coupling using Br_2BTZ (141 mg, 0.5 mmol), 2-napthylboronic acid (215 mg, 1.25 mmol) and K_2CO_3 (140 mg, 1.0 mmol). The reaction was carried for 3 days under nitrogen and the product was purified by recrystallisation from hot methylated spirits resulting in green crystals (162 mg, 83% yield). **^{1}H NMR** ($CDCl_3$, 300 MHz, 25.0 °C) δH 8.51 (d, 2 H), 8.12 (dd, 2 H), 8.03 (d, 2 H), 7.99 (m, 2 H), 7.97 (s, 2 H), 7.93 (m, 2 H), 7.56 (m, 2 H), 7.56 (m, 2 H). **^{13}C NMR** {1H} (CDCl3, 75.5 MHz, 25.0 °C) δC 135.5 (C), 133.4 (C), 133.2 (C), 128.7 (CH), 128.6 (CH), 128.5 (CH), 128.2 (CH), 127.7 (CH), 127.0 (CH), 126.6 (CH), 126.4 (CH).

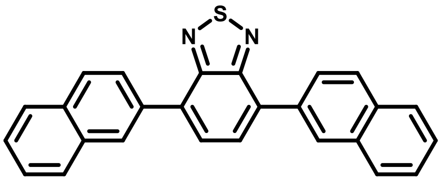

2NapBTZ

Full characterisation in literature procedures from Taylor *et al.*[5]

4,7-Di(thiophen-2-yl)benzo[c][1,2,5]thiadiazole (TpBTZ)

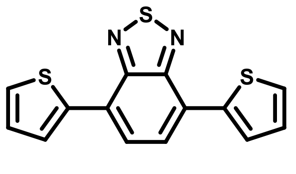

TpBTZ

Synthetic procedure followed as above, *via* Suzuki-Miyaura cross coupling using Br_2BTZ (294 mg, 1 mmol), 2-thienylboronic acid (320 mg, 2.5 mmol) and K_2CO_3 (280 mg, 2.0 mmol). The product was purified by recrystallisation from hot methylated spirits resulting in bright orange crystals (210 mg, 70% yield). **^{1}H NMR** ($CDCl_3$, 300 MHz, 25.0 °C) δH 8.13 (dd, 2 H), 7.89 (s, 2 H), 7.45 (dd, 2 H), 7.22 (dd, 2 H). **^{13}C NMR** {1H} ($CDCl_3$, 75.5 MHz, 25.0 °C) δC 152.7 (C), 139.4 (C), 128.0 (CH), 127.5 (CH), 126.8 (CH), 126.1 (C), 125.8 (CH).

Full characterisation in literature procedures from Taylor *et al.*[5]

4,7-Bis(4-tert-butylphenyl)benzo[c][1,2,5]thiadiazole (tBuBTZ)

Synthetic procedure followed as above, *via* Suzuki-Miyaura cross coupling using Br_2BTZ (294 mg, 1.0 mmol), 4-tert-butylphenylboronic acid (444 mg, 2.5 mmol) and K_2CO_3 (276 mg, 2.0 mmol). The product was purified by recrystallisation from hot methylated spirits resulting in bright yellow needle-crystals (272 mg, 68% yield). **^{1}H NMR** ($CDCl_3$, 300 MHz, 25.0 °C) δH 7.84 (d, 4 H), 7.71 (s, 2 H), 7.51 (d, 4 H), 1.34 (s, 18 H). **^{13}C NMR** {1H} ($CDCl_3$, 75.5 MHz, 25.0 °C) δC 154.3 (C), 151.5 (C), 134.7 (C), 133.2 (C), 129.0 (CH), 128.1 (CH), 125.7 (CH), 34.9 (CH_3), 31.5 (CH).

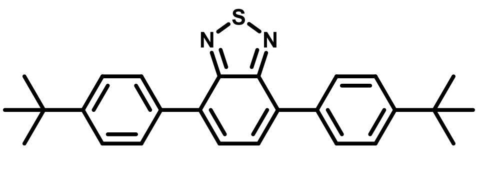

tBuBTZ

Full characterisation in literature procedures from Taylor *et al.*[5]

General procedure for ortho-borylation and transmetallation of 4,7-diarylBTZ

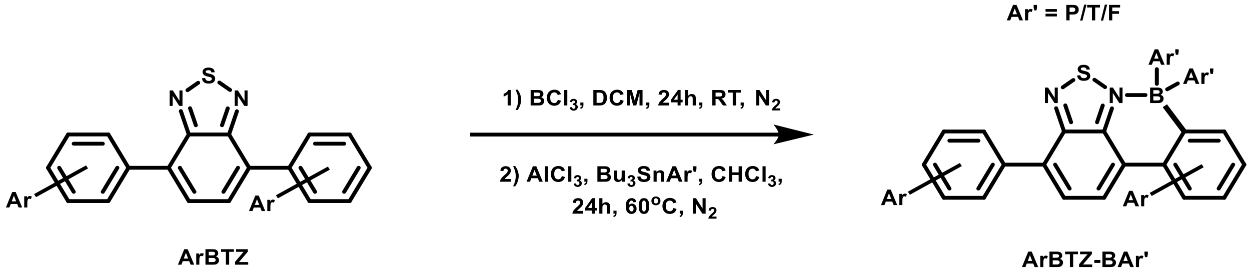

1) *Ortho*-borylation step

4,7-Diarylbenzo[c][1,2,5]thiadiazole (1 equiv.) was added to an oven-dry 2-necked round bottomed flask repeatedly evacuated and back-filled with N_2 gas thrice under Schlenk line. Dry DCM (5 mL) and BCl_3 (1M solution in dry DCM, 6 equiv.) were subsequetly added to the stirring solution. The colour changed immediately from dark red to purple or navy blue depending on the 4,7-diarylbenzothiadiazole used. The reaction was then stirred overnight under constant positive nitrogen pressure. Hence, solvent and residual BCl_3 were removed by vacuum under the Schlenk line with a liquid N_2 trap, resulting in a dry powder of the same colour.

2) Transmetallation step

Anhydrous $AlCl_3$ (3 mg, sublimed and kept under inert conditions) was added to the borylated intermediate (1 equiv.) in an oven-dry 2-necked round bottomed flask and repeatedly evacuated and back-filled with N_2 gas thrice under Schlenk line. Dry chloroform or DCM (10 mL) and the respective tin reagent (2.2 equiv.) were then added to the flask. Hence, the reaction mixture was heated up to reflux temperature with an aluminium heating block under N_2 atmosphere overnight. The reaction progression can be determined by TLC (Ethyl acetate or DCM 100%), as the borylated intermediate is not stable on silica and degrades to the original colour of the ArBTZ, while product maintains its modified colour.

Following this time, the reaction was cooled to room temperature. Hexane was added to the solution to allow product to crash out due to insolubility in the latter (when specified), and residual solid was filtered and washed with hexane to remove the majority of the tin reagent and tributyltin chloride byproduct leftover. Product purification was then finalised *via* either column chromatography, flash chromatography, dry vacuum column chromatography (DCVC),[6] as specified below.

The purification methods varied throughout the series, depending on the solubility of the products in hexane. It is worth of notice that with photocatalysts that had higher solubility in hexane (**PhBTZ-BT**, **PhBTZ-BP**, **tBuBTZ-BT**, **FluBTZ-BT**), the wash step was ignored and after filtering off the crude mixture (removing residual $AlCl_3$) the crude was directly purified *via* chromatography. This resulted often in great loss in yield, as when tributyltin chloride byproduct was not removed beforehand the separation resulted much more challenging, hence obtaining a much smaller fraction of uncontaminated product.

Furthermore, the Lewis acid for the transmetallation step was commercially available $AlCl_3$ (which was sublimed to ensure purity), however, the same reaction was tested also with $Hf(OTf)_4$ for **2NapBTZ-BP** and **TpBTZ-BT**, as this has been proven in literature to have outstanding performance in catalytic Friedel-Crafts acylations and Mannich-type reactions.[7] Hence, due to the variety of purification methods explored and the reagents, the yields of transmetallation reactions are widely variable (spanning from lowest 10% for **tBuBTZ-BT** to highest 71% for **2NapBTZ-BP**), unlike the consistent yields for the borylation itself (>95%) and the Suzuki

coupling (70-85%). The yields have been accounted for in the cost analysis presented in the electronic supporting information document.

Borylation and transmetallation of PhBTZ-BT

Two-step synthetic procedure followed as above, *via* ortho-borylation reaction using PhBTZ (146 mg, 0.5 mmol), and BCl_3 (3 ml, 3 mmol) obtaining a dark red powder. Hence, transmetallation of PhBTZ-BCl (192 mg, 0.5 mmol) was carried through using 2-(Tributylstannyl)thiophene (0.35 mL, 1.1 mmol). Crude product was washed with hexane and purified by column chromatography (hexane/DCM 4:1) resulting in bright red needle-crystals (60 mg, 25% yield). **^{1}H NMR** ($CDCl_3$, 400 MHz, 25.0 °C) δH 8.38 (d, 7.67Hz, 1 H), 8.11 (dd, 1.70-8.10 Hz, 1 H), 7.94 (m, 3 H), 7.68-7.48 (m, 4 H), 7.42-7.32 (m, 4 H), 7.02 (m 4 H). **^{13}C NMR** {1H} ($CDCl_3$, 75.5 MHz, 25.0 °C) δC 153.7 (C), 147.3 (C), 135.4(CH), 135.5 (CH), 132.9 (C), 131.2 (CH), 129.9 (C), 129.1 (CH), 128.9 (CH), 128.3 (C), 127.4 (3x CH) 127.2 (CH), 127.0 (CH), 126.9 (CH), 124.9 (CH), 124.8 (CH), 122.2 (CH), 122.1 (CH). **^{11}B NMR** ($CDCl_3$, 400 MHz, 25.0 °C) δB -2.4(broad). **HRMS** (ESI) m/z: [M + H]+ Calcd for C26H18BN2S3 465.0715; Found 465.0719.

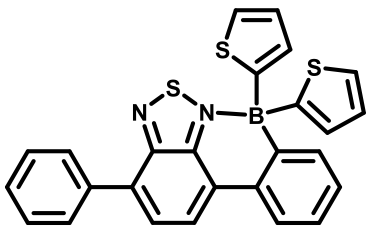

PhBTZ-BT

Borylation and transmetallation of PhBTZ-BP

Two-step synthetic procedure followed as above, *via* ortho-borylation reaction using PhBTZ (146 mg, 0.5 mmol), and BCl_3 (3 ml, 3 mmol) obtaining a dark red powder. Hence, transmetallation of PhBTZ-BCl (192 mg, 0.5 mmol) was carried through using tributylphenylstannane (0.34 mL, 1.1 mmol). Crude product was washed with hexane and purified by column chromatography (hexane/DCM 4:1) resulting in bright red powder (32 mg, 15% yield). **^{1}H NMR** ($CDCl_3$, 400 MHz, 25.0 °C) δH 8.37 (d, 7.76Hz, 1 H), 8.13 (d, 7.73Hz, 1 H), 7.96 (dd, 8.12-1.74 Hz, 3 H), 7.55 (m, 4 H), 7.34 (m, 2 H), 7.25 (m, 10 H). **^{13}C NMR** {1H} ($CDCl_3$, 75.5 MHz, 25.0 °C) δC 135.4 (C), 134.8 (CH), 133.6 (CH), 132.5 (C), 131.1 (CH), 130.2 (C), 129.1 (CH), 128.9 (CH), 127.5 (CH), 126.2 (CH), 126.0 (CH), 124.3 (CH), 122.2 (CH). **^{11}B NMR** ($CDCl_3$, 400 MHz, 25.0 °C) δB 1.6(broad). **HRMS** (ESI) m/z: [M + Na]+ Calcd for C30H21BN2SNa 465.0719 ; Found 465.0687.

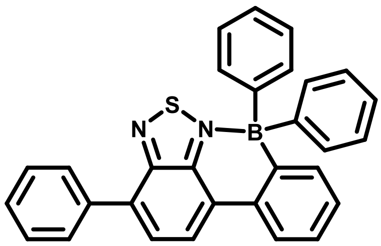

PhBTZ-BP

Borylation and transmetallation of PhBTZ-BF

Two-step synthetic procedure followed as above, *via* ortho-borylation reaction using PhBTZ (146 mg, 0.5 mmol), and BCl_3 (3 ml, 3 mmol) obtaining a red powder. Hence, transmetallation of PhBTZ-BCl (80 mg, 0.2 mmol) was carried through using 2-(Tributylstannyl)furan (0.15 mL, 0.44 mmol). Crude product was purified by washing with hexane, followed by column chromatography (hexane/DCM 1:4 to DCM 100%) resulting in bright red powder (102 mg, 47% yield). **^{1}H NMR** ($CDCl_3$, 400 MHz, 25.0 °C) δH 7.73 (m, 3 H), 7.63 (d, 8.25 Hz, 2 H), 7.48 (m, 12 H). **^{13}C NMR** {1H} ($CDCl_3$, 75.5 MHz, 25.0 °C) δC 134.2 (CH), 129.4 (CH), 129.0 (CH), 128.6 (CH), 128.4 (CH), 128.1 (CH), 127.3 (CH), 127.0 (CH). **^{11}B NMR** ($CDCl_3$, 400 MHz, 25.0 °C) δB 2.1(broad). **HRMS** (ESI) m/z: [M]+ Calcd for C26H17BN2O2S 432.1543; Found 432.1563.

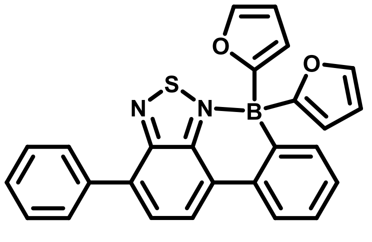

PhBTZ-BF

Borylation and transmetallation of tBuBTZ-BT

Two-step synthetic procedure followed as above, *via* ortho-borylation reaction using tBuBTZ (200 mg, 0.5 mmol), and BCl_3 (3 ml, 3 mmol) obtaining a dark purple powder. Hence, transmetallation of tBuBTZ-BCl (240 mg, 0.5 mmol) was carried through using 2-(Tributylstannyl)thiophene (0.35 mL, 1.1 mmol) for 5 hours. Crude product was purified by flash column chromatography (hexane/ethyl acetate 95:5) resulting in dark burgundy powder (20 mg, 7% yield). **^{1}H NMR** ($CDCl_3$, 400 MHz, 25.0 °C) δH 8.23 (d, 7.23 Hz, 1 H), 7.95 (d, 8.89 Hz, 1 H), 7.83 (d, 7.23 Hz, 1 H), 7.79 (d, 8.34 Hz, 2 H), 7.70 (d, 1.67 Hz, 1 H), 7.50 (d, 8.34 Hz, 2 H), 7.35 (dd, 2.22-9.45 Hz, 1 H), 7.27 (dd, 1.17-4.68 Hz, 2 H), 6.94 (m, 4 H), 1.32 (s, 9 H), 1.25 (s, 9 H). **^{13}C NMR** {1H} ($CDCl_3$, 75.5 MHz, 25.0 °C) δC 153.7 (C), 152.2 (C), 151.6 (C), 147.3 (C), 132.7 (C), 132.3 (CH), 132.1 (C), 130.8 (CH), 128.7 (CH), 128.1 (C), 127.5 (C), 127.4 (CH),127.1 (CH), 125.9 (CH), 124.3 (CH), 123.9 (CH), 121.8 (CH), 31.3 (CH_3), 31.2 (CH_3). **^{11}B NMR** ($CDCl_3$, 400 MHz, 25.0 °C) δB -2.2(broad). **HRMS** (ESI) m/z: [M]+ Calcd for C34H33BN2S3 577.1972; Found 577.1978 .

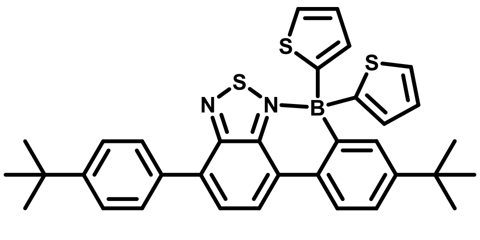

tBuBTZ-BT

Borylation and transmetallation of tBuBTZ-BP

Two-step synthetic procedure followed as above, *via* ortho-borylation reaction using tBuBTZ (150 mg, 0.375 mmol), and BCl_3 (2.25 ml, 2.25 mmol) obtaining a dark purple powder. Hence, transmetallation of tBuBTZ-BCl (173 mg, 0.36 mmol) was carried out using tributylphenylstannane (0.27 mL, 0.88 mmol) for 4 hours. Crude product was purified by hexane wash, followed by DCVC (ethyl acetate/hexane 1:19 to 1:0) yielding a bright burgundy powder (130 mg, 63% yield). **^{1}H NMR** ($CDCl_3$, 400 MHz, 25.0 °C) δH 8.28 (d, 8.15 Hz, 1 H), 8.03 (d, 9.16 Hz, 1 H), 7.91 (d, 7.67 Hz, 1 H), 7.87 (d, 8.85 Hz, 2 H), 7.58 (m, 3 H), 7.36 (dd, 1.77-8.26 Hz, 1 H), 7.23 (m, 10 H), 1.40 (s, 9 H), 1.28 (s, 9 H). **^{13}C NMR** {1H} ($CDCl_3$, 75.5 MHz, 25.0 °C) δC ($CDCl_3$, 75.5 MHz, 25.0 °C) δC 153.7 (C), 152.2 (C), 151.6 (C), 147.3 (C), 132.7 (C), 133.5 (CH), 132.7 (C), 131.8 (CH), 130.8 (CH), 128.7 (CH), 127.5 (CH), 125.9 (CH),123.8 (CH), 123.3 (CH), 121.9 (CH), 121.3 (C), 31.3 (CH_3), 31.2 (CH_3). **^{11}B NMR** ($CDCl_3$, 400 MHz, 25.0 °C) δB 4.2(broad). **HRMS** (ESI) m/z: [M]+ Calcd for C38H37BN2S 565.2843 ; Found 565.2850 .

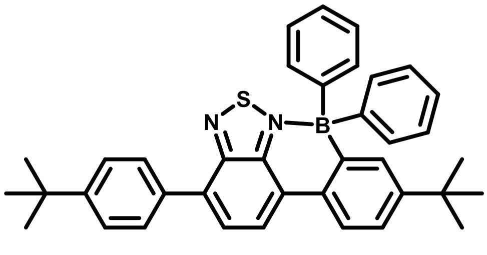

tBuBTZ-BP

Borylation and transmetallation of 2NapBTZ-BT

Two-step synthetic procedure followed as above, *via* ortho-borylation reaction using 2NapBTZ (97 mg, 0.25 mmol), and BCl_3 (1.5 ml, 1.5 mmol) obtaining a dark purple powder. Hence, transmetallation of 2NapBTZ-BCl (115 mg, 0.25 mmol) was carried through using 2-(Tributylstannyl)thiophene (0.16 mL, 0.55 mmol). Crude product was purified by hexane wash, followed by short silica plug (hexane/DCM 4:1) resulting in dark purple powder (50 mg, 35% yield). **^{1}H NMR** ($CDCl_3$, 400 MHz, 25.0 °C) δH 8.84 (d, 7.60 Hz, 1 H), 8.62 (d, 5.06 Hz, 2 H), 8.53 (s, 2 H), 8.20 (d, 8.07 Hz, 1 H), 8.12-7.93 (m, 9 H), 7.62-7.50 (m, 6 H). **^{13}C NMR** {1H} ($CDCl_3$, 75.5 MHz, 25.0 °C) δC ($CDCl_3$, 75.5 MHz, 25.0 °C) δC 134.3 (CH), 133.5 (C), 131.9 (C), 131.3 (CH), 131.2 (CH), 128.9 (CH), 128.7 (C), 128.6 (CH), 128.5 (CH), 128.4 (CH), 128.4 (CH), 128.3 (C), 128.2 (CH), 128.0 (CH), 127.9 (CH),

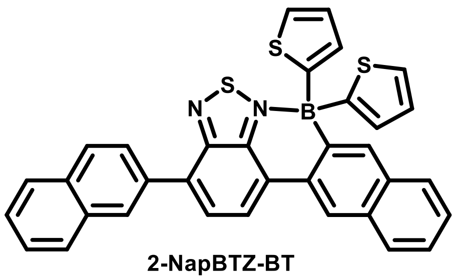

2-NapBTZ-BT

127.8 (CH), 127.6 (CH), 127.5 (CH), 127.3 (CH), 126.7 (C), 126.3 (C), 126.2 (CH), 125.7(CH), 125.7(CH), 121.8 (CH), 121.9 (CH). ^{11}**B NMR** ($CDCl_3$, 400 MHz, 25.0 °C) δB 6.2 (broad). **HRMS** (ESI) m/z: [M + H]+ Calcd for C34H22BN2S3 565.3866 ; Found 565.3859 .

Borylation and transmetallation of 2NapBTZ-BP

Two-step synthetic procedure followed as above, *via* ortho-borylation reaction using 2NapBTZ (97 mg, 0.25 mmol), and BCl_3 (1.5 ml, 1.5 mmol) obtaining a dark purple powder. Hence, transmetallation of 2NapBTZ-BCl (110 mg, 0.24 mmol) was carried out using tributylphenylstannane (0.16 mL, 1.1 mmol). Crude product was purified by washing with hexane, followed by flash chromatography (ethyl acetate/hexane 10/90 ramping up to ethyl acetate 100%) yielding a purple powder (98 mg, 71% yield). ^{1}**H NMR** ($CDCl_3$, 400 MHz, 25.0 °C) δH 8.65 (s, 1 H), 8.63 (d, 8.07 Hz, 1 H), 8.50 (s, 1 H), 8.12 (s, 7.63 Hz, 1 H), 8.03 (m, 2 H), 8.00-7.87 (m, 5 H), 7.72 (dd, 3.5-6.1 Hz, 1H), 7.57 (dd, 3.5-6.1 Hz, 2 H), 7.42 (dd, 3.5-6.1 Hz, 2 H), 7.33-7.28 (m, 3 H), 7.26-7.15 (m, 7 H). ^{13}**C NMR** {1H} ($CDCl_3$, 75.5 MHz, 25.0 °C) δC ($CDCl_3$, 75.5 MHz, 25.0 °C) δC 153.8 (C), 134.0 (C), 133.9 (CH), 133.7 (C), 133.4 (C), 133.4 (C), 132.8 (C), 132.8 (C), 132.1 (C), 131.2 (CH), 129.5 (C), 128.8 (CH), 128.8 (C), 128.6 (CH), 128.1 (CH), 127.9 (CH), 127.8 (CH), 127.6 (CH), 127.0 (CH), 126.7 (CH), 126.5 (CH), 126.3 (CH), 126.0(CH), 125.4 (CH), 125.2 (CH), 121.8 (CH). ^{11}**B NMR** ($CDCl_3$, 400 MHz, 25.0 °C) Δb 4.0 (broad). **HRMS** (ESI) m/z: [M]+ Calcd for C38H25BN2S 550.6369; Found 550.6360.

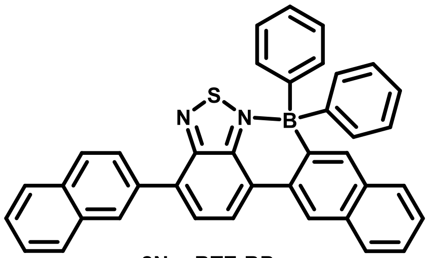

2NapBTZ-BP

Borylation and transmetallation of FluBTZ-BT

Two-step synthetic procedure followed as above, *via* ortho-borylation reaction using FluBTZ (228 mg, 0.25 mmol), and BCl_3 (1.5 ml, 1.5 mmol) obtaining a dark blue powder. Hence, transmetallation of FluBTZ-BCl (240 mg, 0.25 mmol) was carried through using 2-(Tributylstannyl)thiophene (0.16 mL, 0.55 mmol) and sublimed anhydrous aluminium chloride. Crude product was purified by base-treated short silica plug (hexane/DCM 95:5) followed by column chromatography (hexane/ethyl acetate 85:15) resulting in dark purple powder (154 mg, 56% yield). ^{1}**H NMR** ($CDCl_3$, 400 MHz, 25.0 °C) δH 8.38 (d, 6.72 Hz, 1 H), 7.98 (s, 1 H), 7.96 (d, 6.72 Hz, 1 H), 7.89 (s, 1H), 7.85 (m, 3 H), 7.69 (m, 1 H), 7.60 (m, 1 H), 7.31 (m, 8 H), 7.02 (m, 4 H), 1.96 (m, 8 H), 1.02 (m, 48 H), 0.72, (t, 12 H). ^{13}**C NMR** {1H} ($CDCl_3$, 75.5 MHz, 25.0 °C) δC 154.0 (C), 151.3 (C), 149.8 (C), 142.2 (C), 140.3 (C), 132.8 (C), 131.3 (CH), 131.3 (CH_2), 130.8 (CH), 130.8 (CH_2), 128.7 (C), 127.9-126.0 (m, CH), 127.9-126.0 (m, CH_2), 124.6-116.11 (m, CH_2), 124.6-116.11 (m, CH), 55.3 (C), 54.9 (C), 40.68 (CH_3), 40.30 (CH_3), 31.84 (CH), 31.6 (CH_3), 30.1 (CH), 29.28 (CH), 23.9 (CH_3), 22.6 (CH), 14.06 (CH_2). ^{11}**B NMR** ($CDCl_3$, 400 MHz, 25.0 °C) δB -1.9 (broad). **HRMS** (ESI) m/z: [M + H]+ Calcd for C72H82BN2O4S3 1089.6353 ; Found 1089.6354.

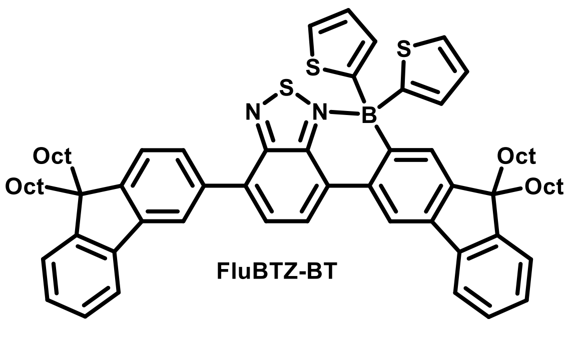

FluBTZ-BT

Borylation and transmetallation of TpBTZ-BT

Two-step synthetic procedure followed as above, *via* ortho-borylation reaction using TpBTZ (75 mg, 0.25 mmol), and BCl_3 (1.5 ml, 1.5 mmol) obtaining a navy-blue powder. Hence, transmetallation of TpBTZ-BCl (95 mg, 0.25 mmol) was carried through using 2-(Tributylstannyl)thiophene (0.16 mL, 0.55 mmol). Dark blue crude product was purified by hexane wash, followed by flash chromatography (ethyl acetate/hexane 10/90 ramping up to ethyl acetate 100%) resulting in a blue powder (83 mg, 70% yield). **^{1}H NMR** ($CDCl_3$, 400 MHz, 25.0 °C) δH 8.03 (dd, 1.43-3.8 Hz, 1 H), 7.91 (d, 7.39 Hz, 1 H), 7.73 (d, 7.39 Hz, 1 H), 7.46 (dd, 1.17-5.18 Hz, 1 H), 7.41 (d, 4.83 Hz, 1 H), 7.39-7.34 (m, 2 H), 7.24-7.17 (m, 2 H), 7.07-7.00 (m, 5 H). **^{13}C NMR** {1H} ($CDCl_3$, 75.5 MHz, 25.0 °C) δC 136.7 (C), 132.2 (CH), 129.6 (CH), 127.7 (CH), 127.3 (CH), 126.7 (CH), 126.6 (CH), 126.4 (CH), 126.3 (CH), 126.2 (CH), 123.7 (C), 122.3 (CH). **^{11}B NMR** ($CDCl_3$, 400 MHz, 25.0 °C) δB -1.3 (broad). **HRMS** (ESI) m/z: [M + H]+ Calcd for C22H14BN2S5 476.9848; Found 476.9852.

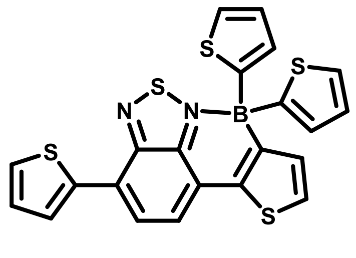

TpBTZ-BT

Borylation and transmetallation of TpBTZ-BP

Two-step synthetic procedure followed as above, *via* ortho-borylation reaction using TpBTZ (75 mg, 0.25 mmol), and BCl_3 (1.5 ml, 1.5 mmol) obtaining a navy-blue powder. Hence, transmetallation of TpBTZ-BCl (95 mg, 0.25 mmol) was carried through using tributylphenylstannane (0.16 mL, 0.55 mmol) with sublimed aluminium chloride. Crude product was purified by washing with hexane followed by flash chromatography (ethyl acetate/hexane 10/90 ramping up to ethyl acetate 100%) yielding a navy-blue powder (62 mg, 54% yield). **^{1}H NMR** ($CDCl_3$, 400 MHz, 25.0 °C) δH 8.04 (dd, 1.1-3.6 Hz, 1 H), 7.93 (d, 7.8 Hz, 1 H), 7.70 (d, 7.8 Hz, 1 H), 7.46 (dd, 1.2-5.4 Hz, 1 H), 7.38 (d, 5.6 Hz, 1 H), 7.25-7.14 (m, 11 H), 7.12 (d, 4.9 Hz, 1 H). **^{13}C NMR** {1H} ($CDCl_3$, 75.5 MHz, 25.0 °C) δC 151.6 (C), 138.7 (C), 133.6 (C), 133.1 (CH), 131.30 (C), 128.8 (C), 127.7 (CH), 126.7 (CH), 127.6 (CH), 127.0 (CH), 126.3 (CH), 122.2 (CH), 118.5 (C). **^{11}B NMR** ($CDCl_3$, 400 MHz, 25.0 °C) δB 0.7 (broad). **HRMS** (ESI) m/z: [M + H]+ Calcd for C26H17BN2S3 465.0720; Found 465.0724.

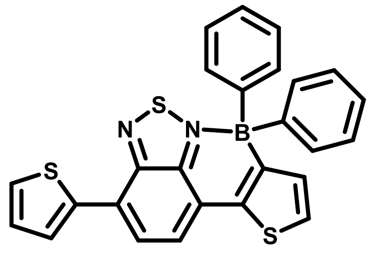

TpBTZ-BP

Safety statement

The bromination of BTZ was conducted using literature routes employing N-Bromosuccinimide rather than neat bromine as brominating agent (as stated in the manuscript), to reduce bromine usage and spillage risks. The reaction was run connecting a trap containing an aqueous solution of sodium thiosulfate to the reflux apparatus, to quench any escape of bromine from the reaction vessel.

The Suzuki-Miyaura cross coupling reaction was carried using a Schlenk line and a condenser to carry the reaction at reflux temperature. No obvious risk was detected during the reaction.

The borylation reaction require the use of boron trichloride, which was purchased in a 1M solution (Dichloromethane) with a septum capped bottle. The reaction was also carried under positive nitrogen pressure using the Schlenk line to ensure dryness; Thus, addition of the boron reagent into the vessel was achieved by syringe. Once the reaction was complete, the excess boron trichloride was removed by vacuum under Schlenk line, with employment of a liquid nitrogen trap to impede the reagent to reach the vacuum pump.

The following transmetallation step did not require any abnormal laboratory safety precaution arising from the use of tin reagents. Analogous BTZ compounds have been reported as non-harmful.[5]

2. UV-Visible Absorption and Emission spectra

Absorption spectra

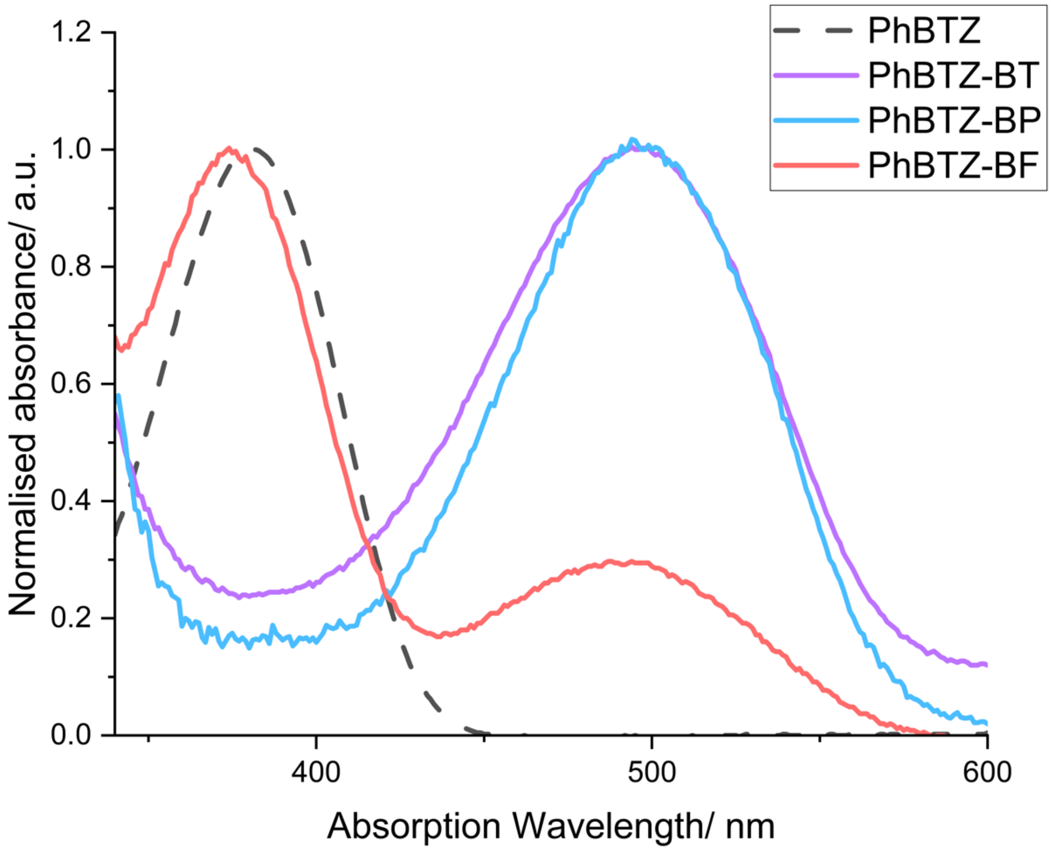

Figure S1. Absorption spectrum of PhBTZ.

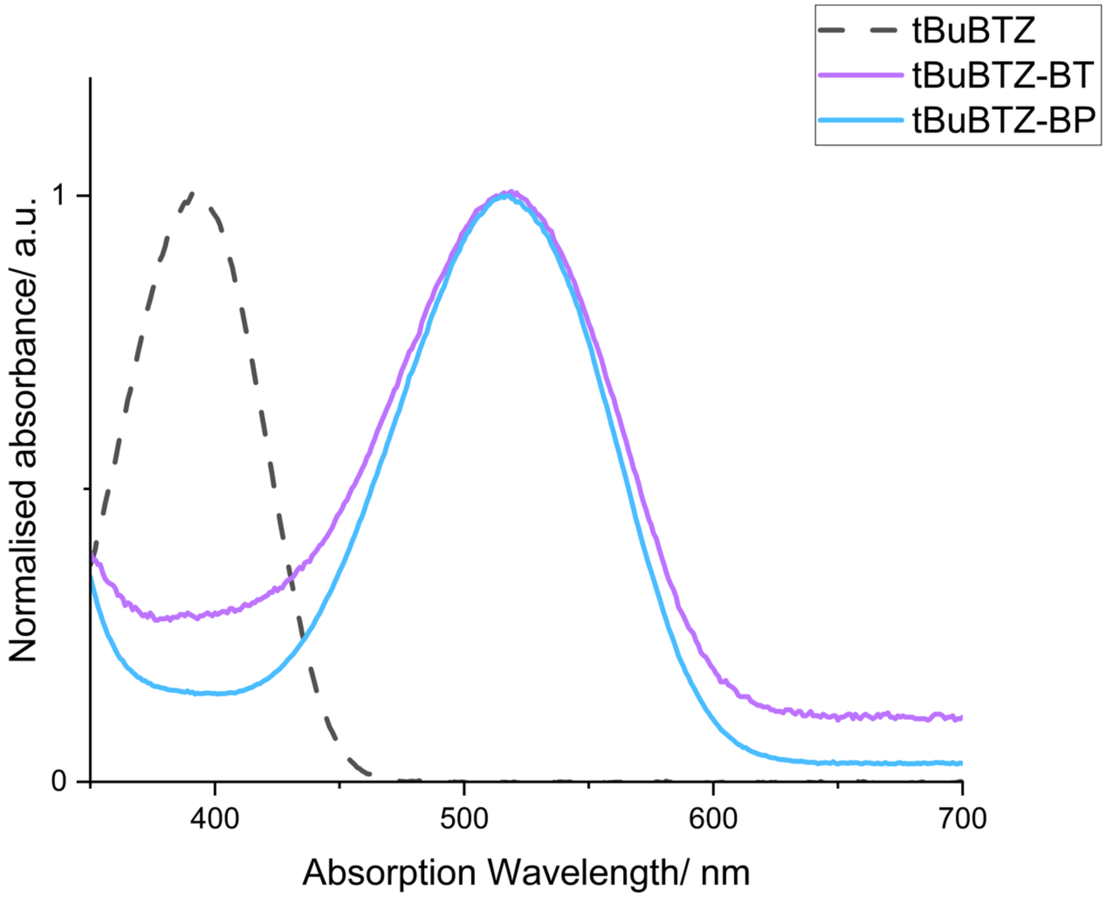

Figure S2. Absorption spectrum of tBuBTZ.

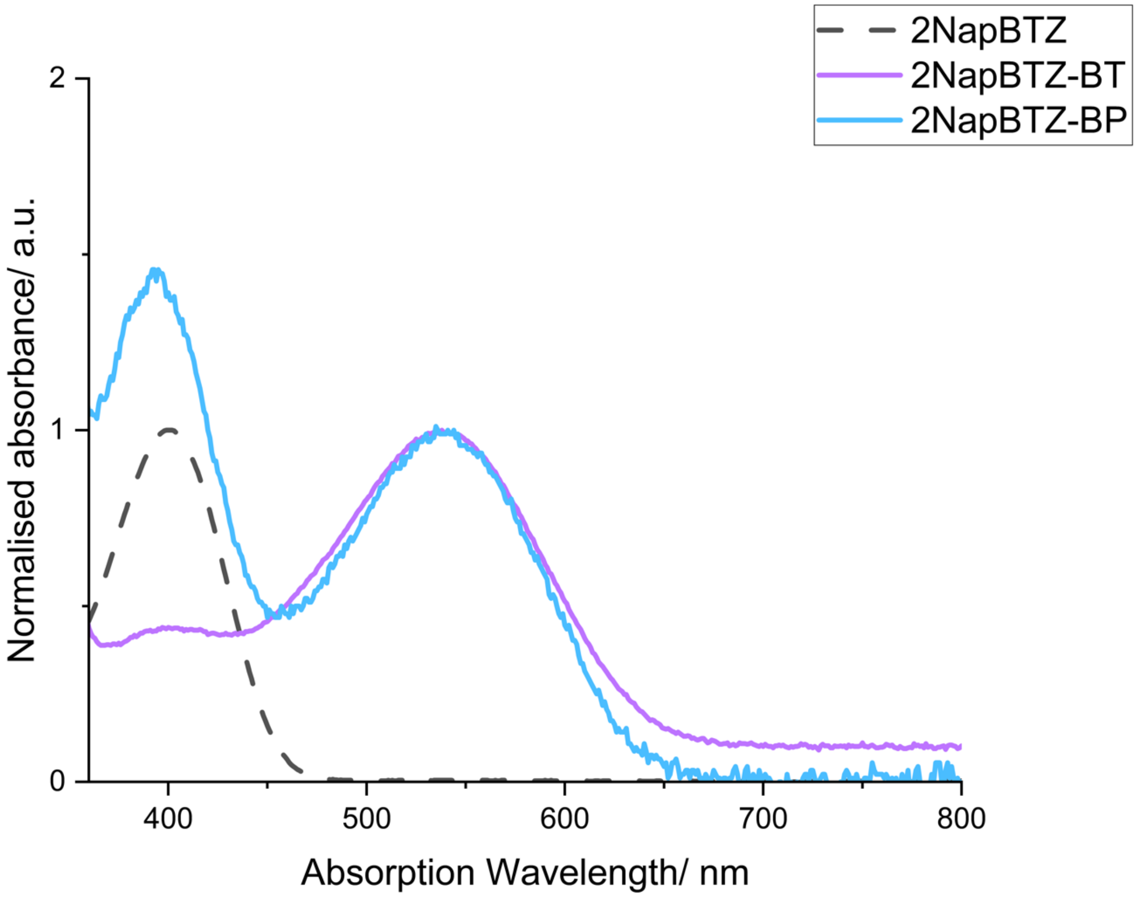

Figure S3. Absorption spectrum of 2NapBTZ.

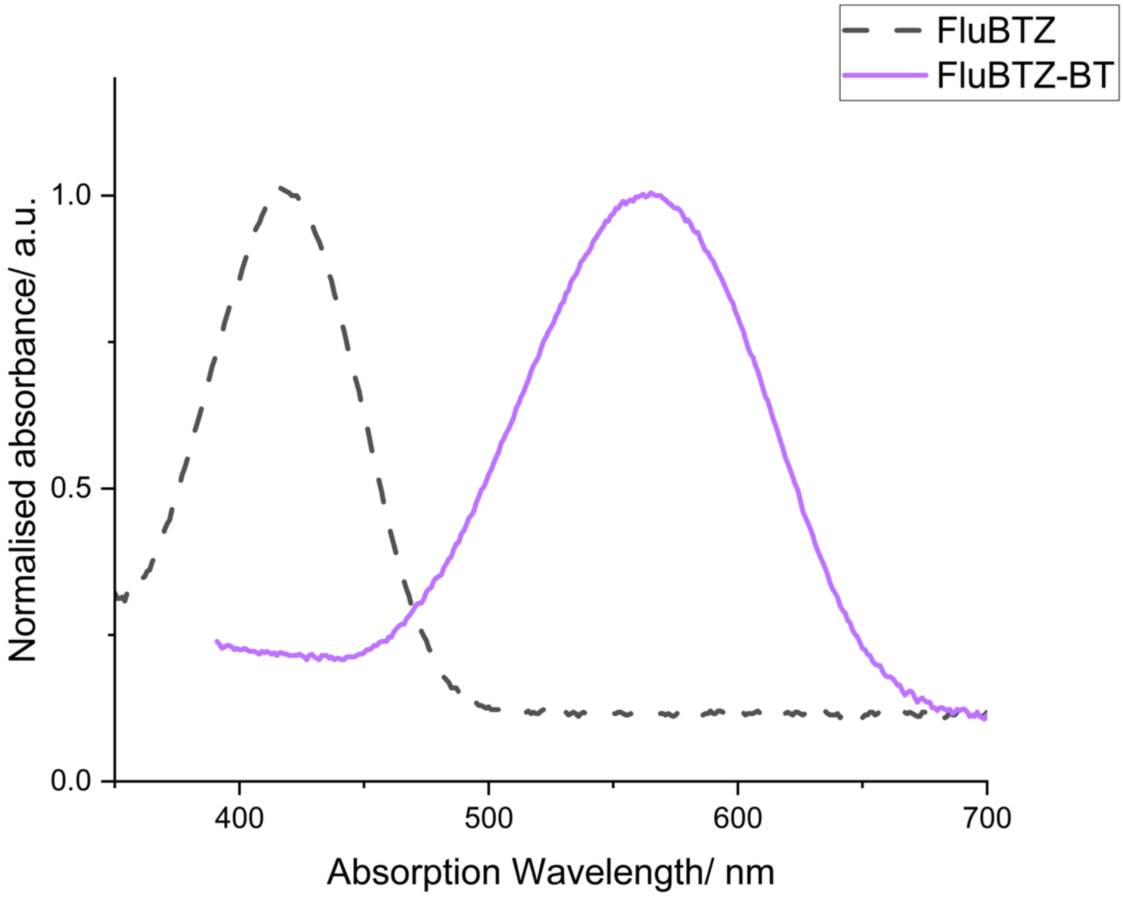

Figure S4. Absorption spectrum of FluBTZ.

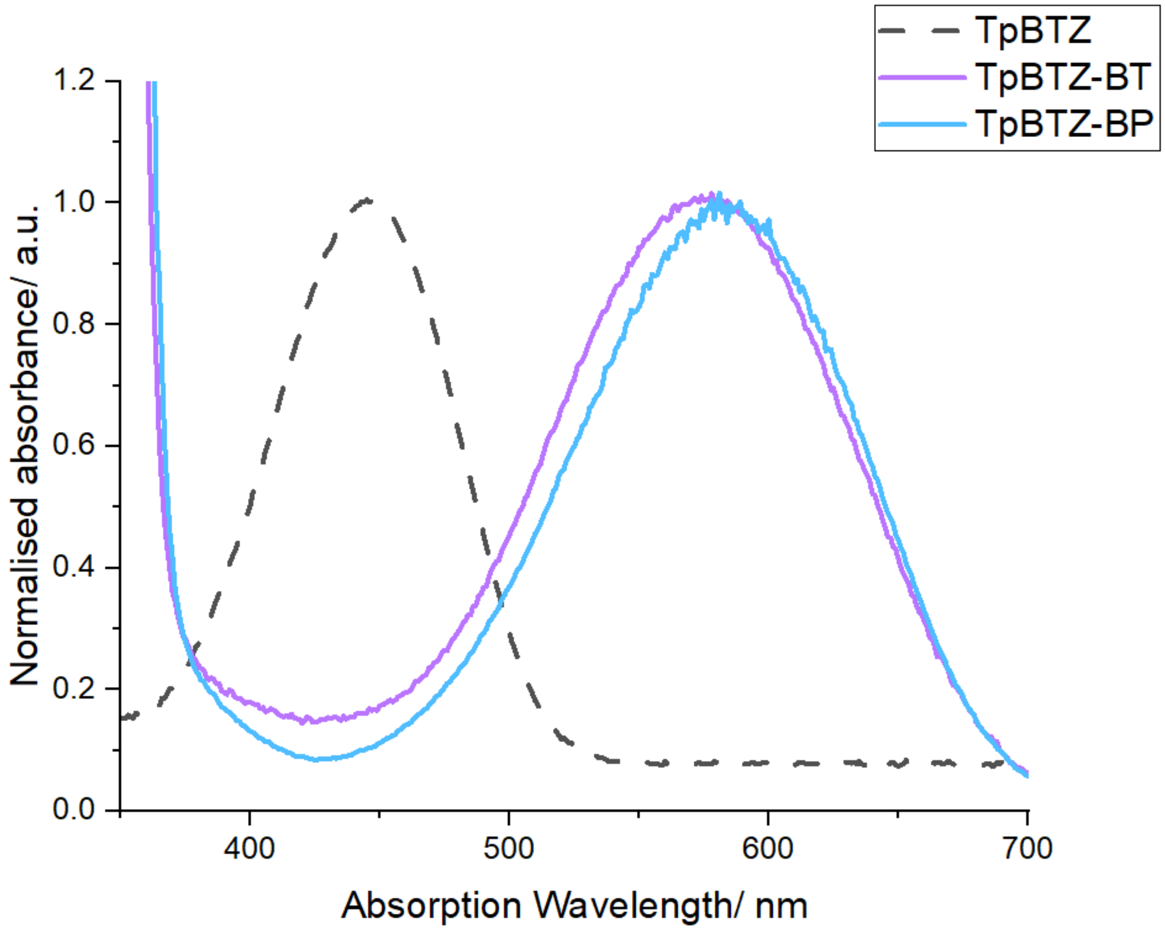

Figure S5. Absorption spectrum of TpBTZ.

Emission spectra

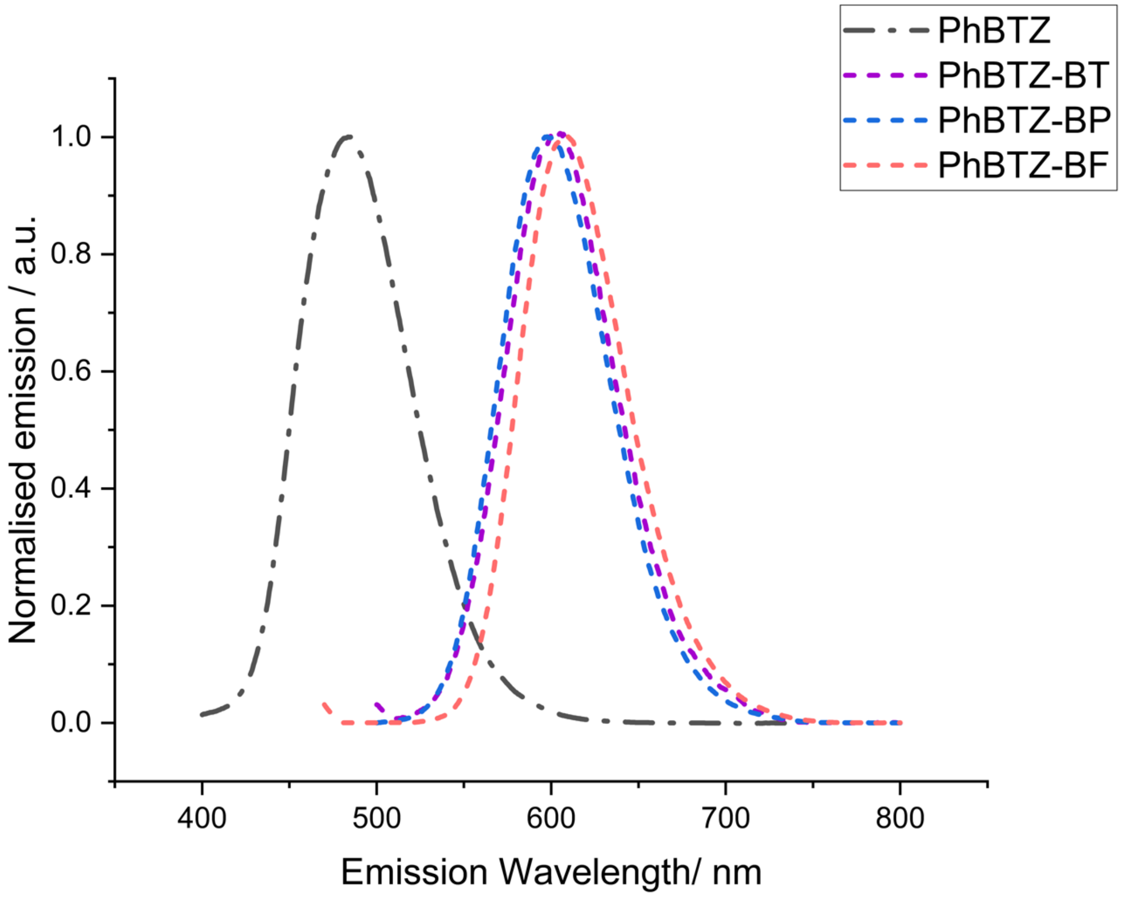

Figure S6. Emission spectrum of PhBTZ.

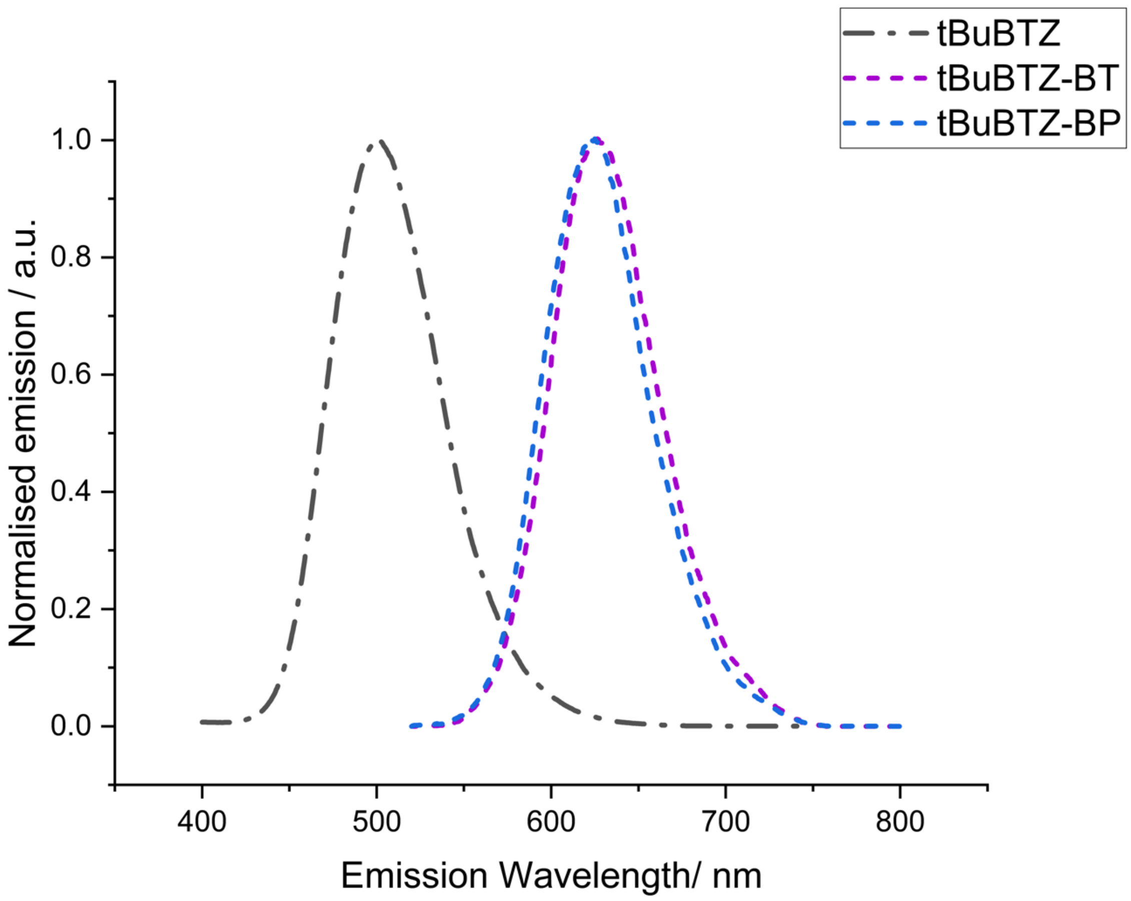

Figure S7. Emission spectrum of tBuBTZ.

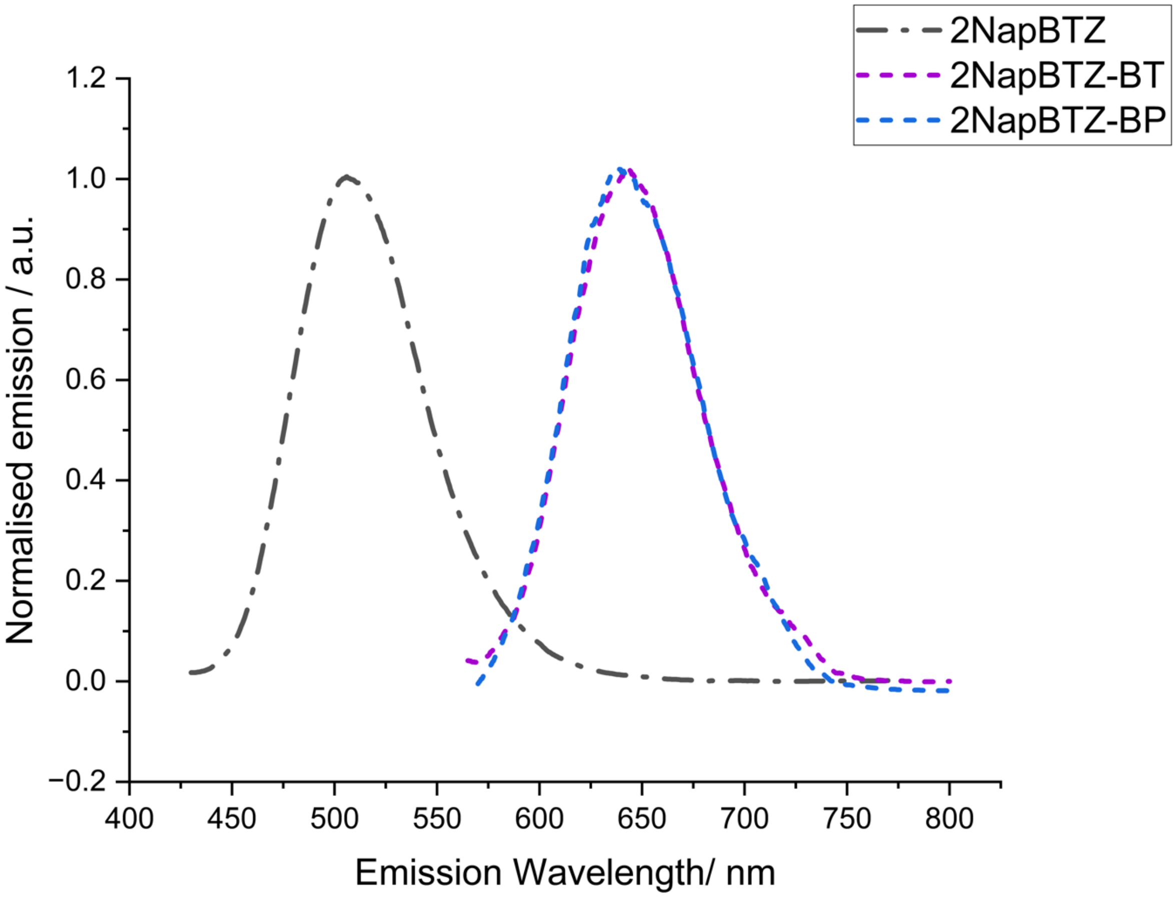

Figure S8. Emission spectrum of 2NapBTZ.

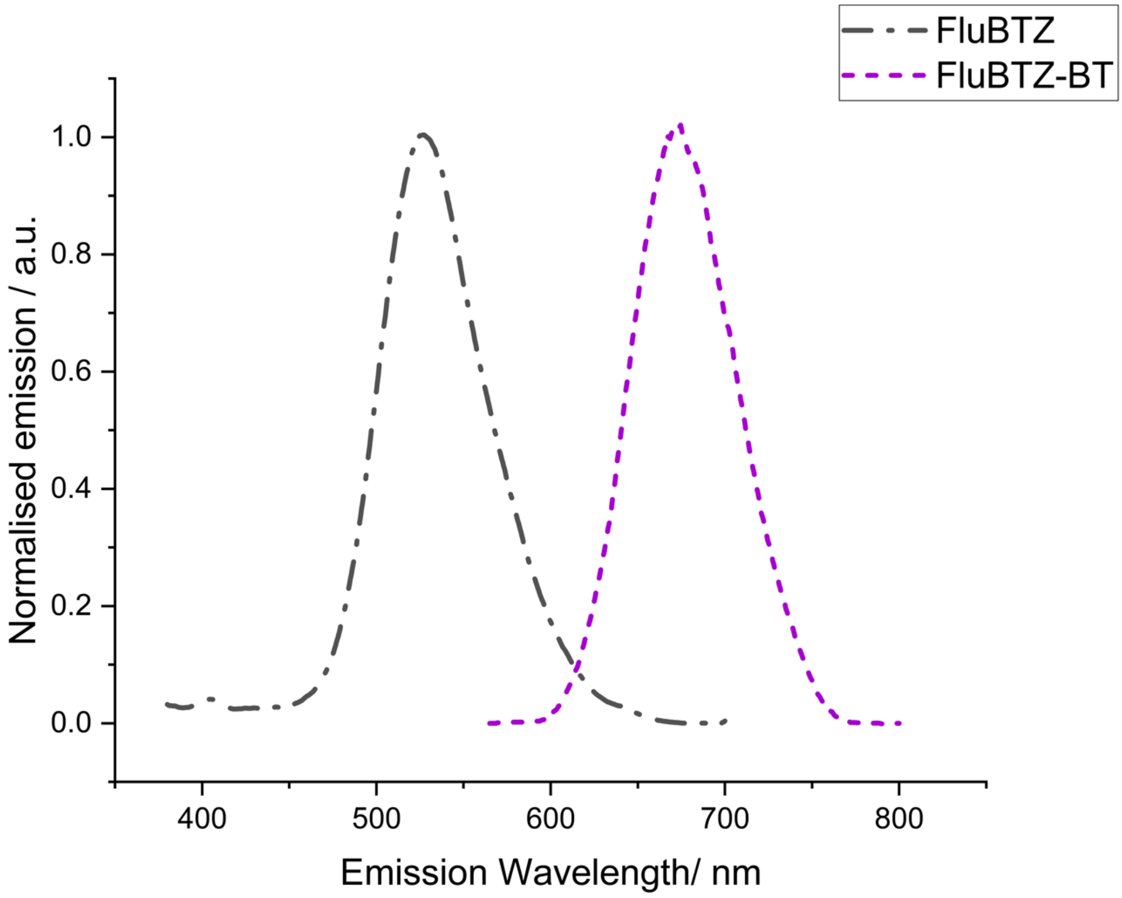

Figure S9. Emission spectrum of FluBTZ.

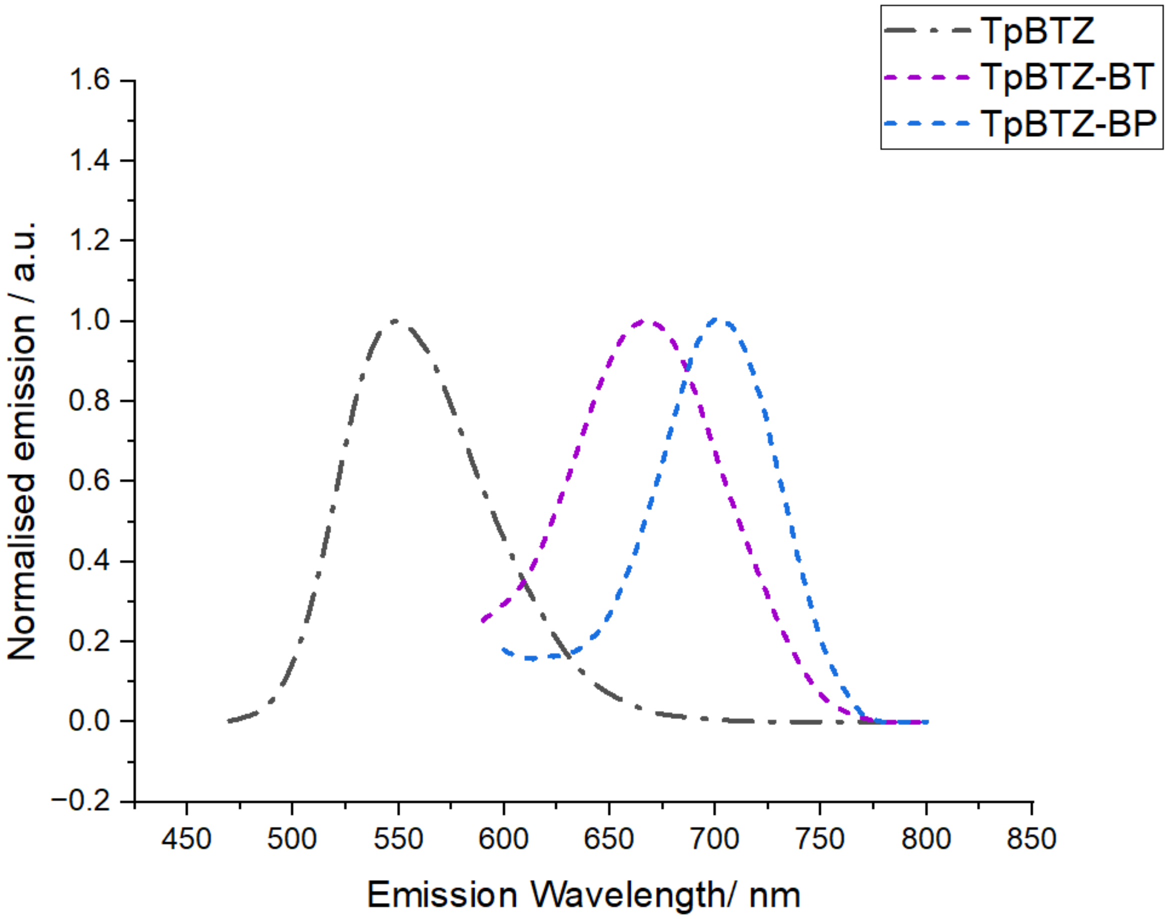

Figure S10. Emission spectrum of TpBTZ.

3. NMR Spectra

All NMR spectra below were collected in $CDCl_3$ (1H NMR peak: 7.26 ppm, s) unless specified otherwise.
The ^{11}B NMRs of the 10 photocatalysts present only a broad singlet from 5(-5) ppm. This is attributed to the higher grade of relaxation of the tetrahedral boron atom in the photocatalyst structure. The boron trichloride starting material presents instead a typical sharper peak generally at 21 ppm; whilst the empty borosilicate glass does not show any broad peak but background noise.[8,9]
*Known residual solvent impurity (eg. Dichloromethane, hexane, ethyl acetate, acetone, water) vacuum grease.[10]
Each catalyst was dried under vacuum (8.0 mbar) at 50 °C prior to NMR analysis.

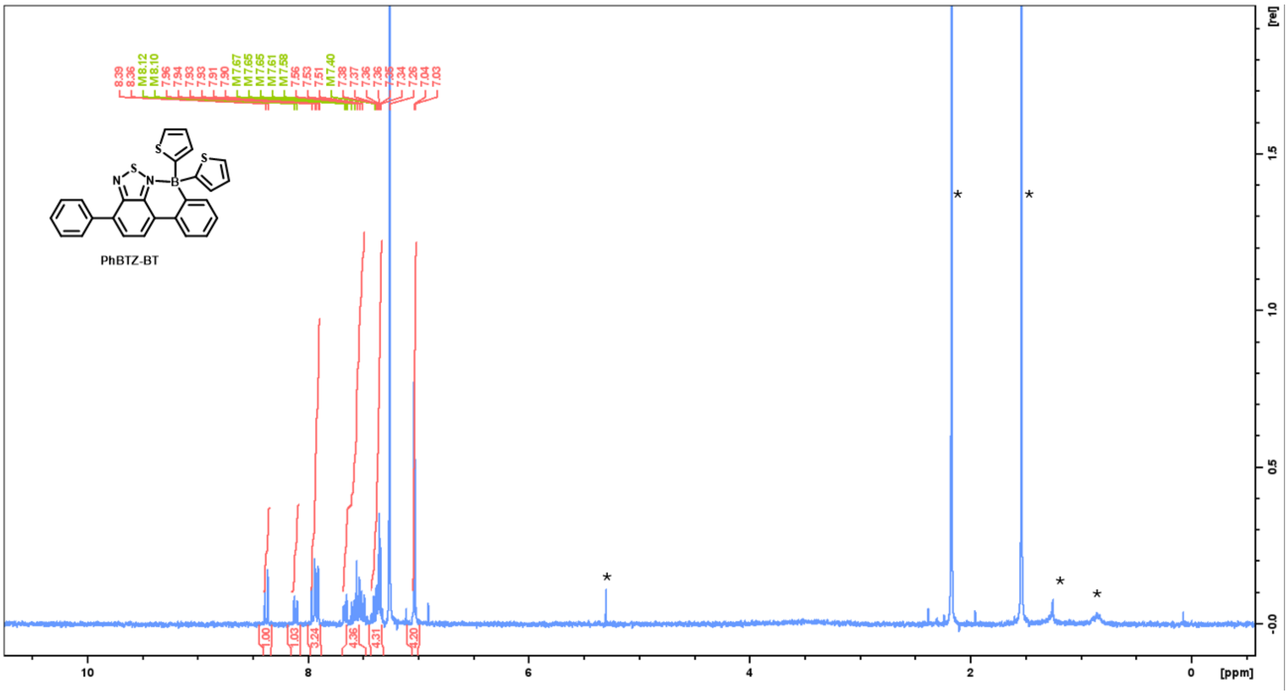

Figure S11. ^1H NMR (400 MHz) spectrum of PhBTZ-BT in $CDCl_3$.

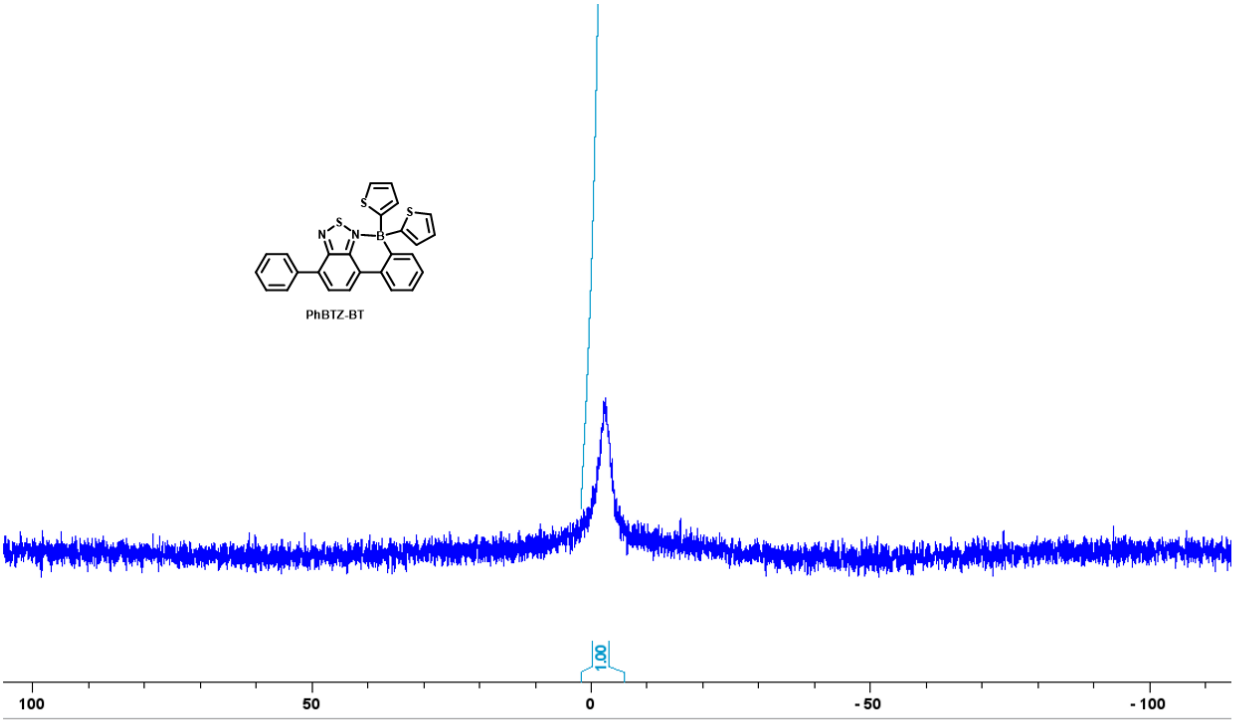

Figure S12. ^{11}B NMR (400 MHz) spectrum of PhBTZ-BT in $CDCl_3$.

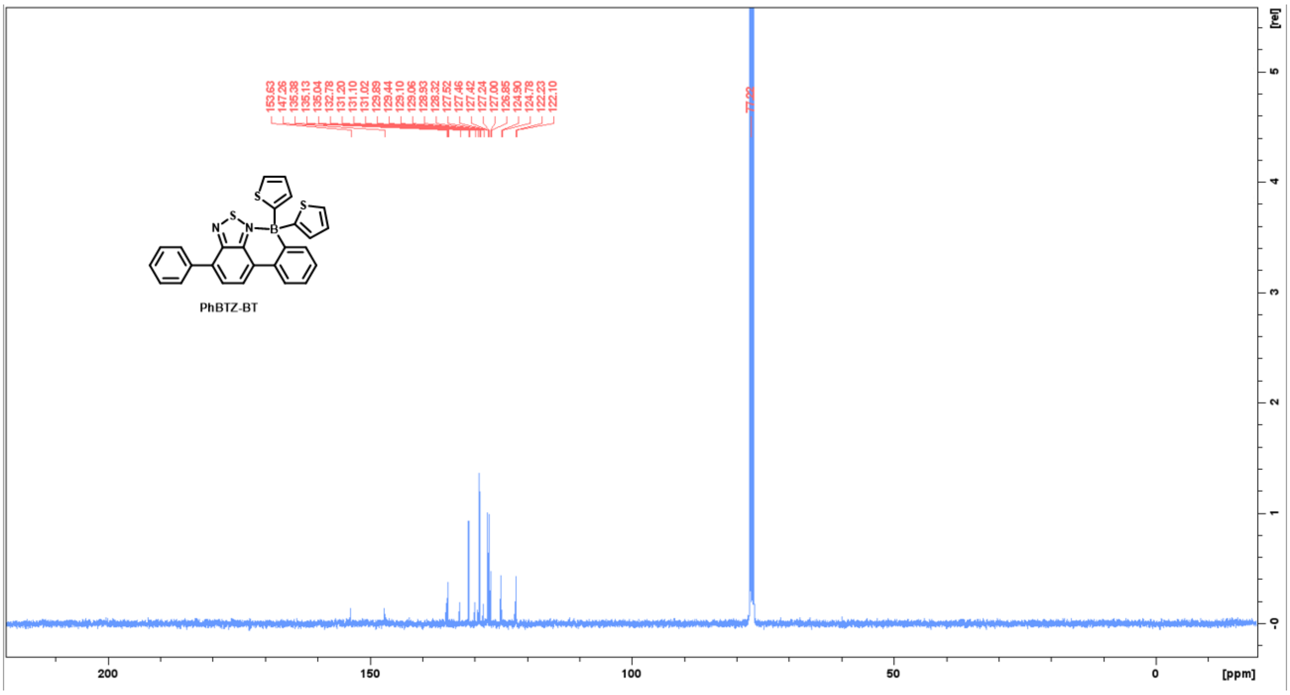

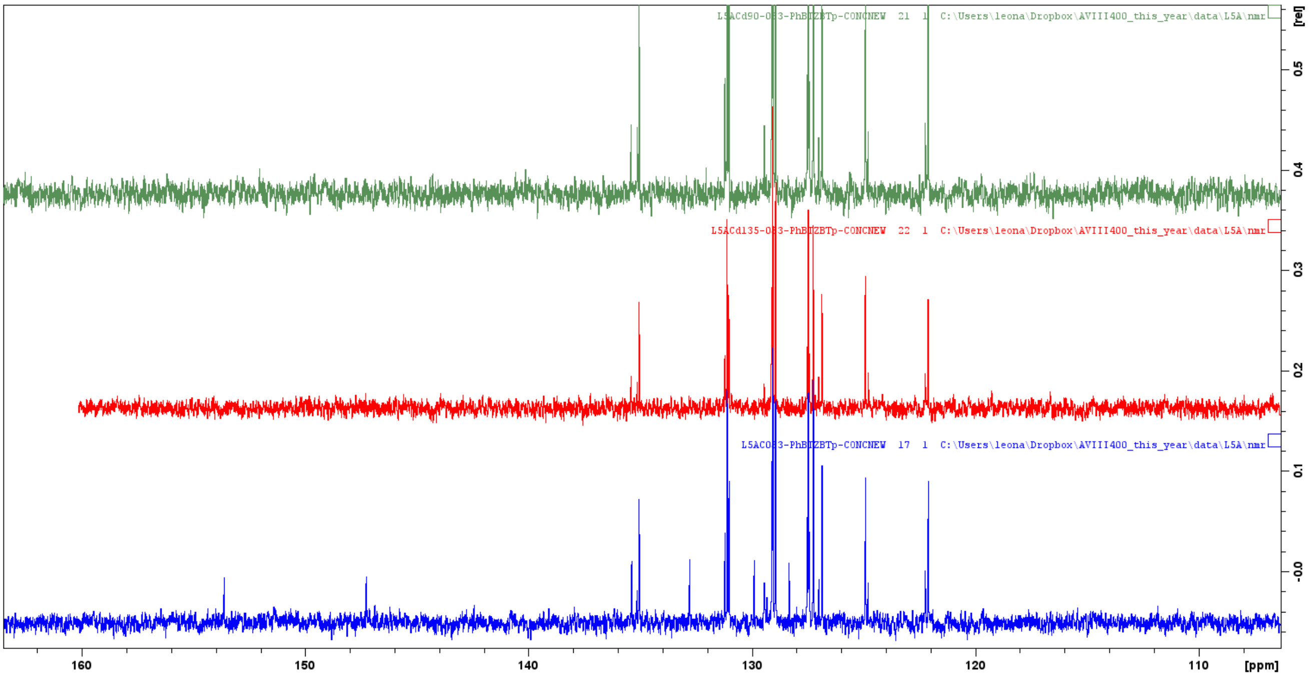

Figure S13. $^{13}C\{^1H\}$ NMR, DEPT90 and DEPT135 spectra of PhBTZ-BT in $CDCl_3$.

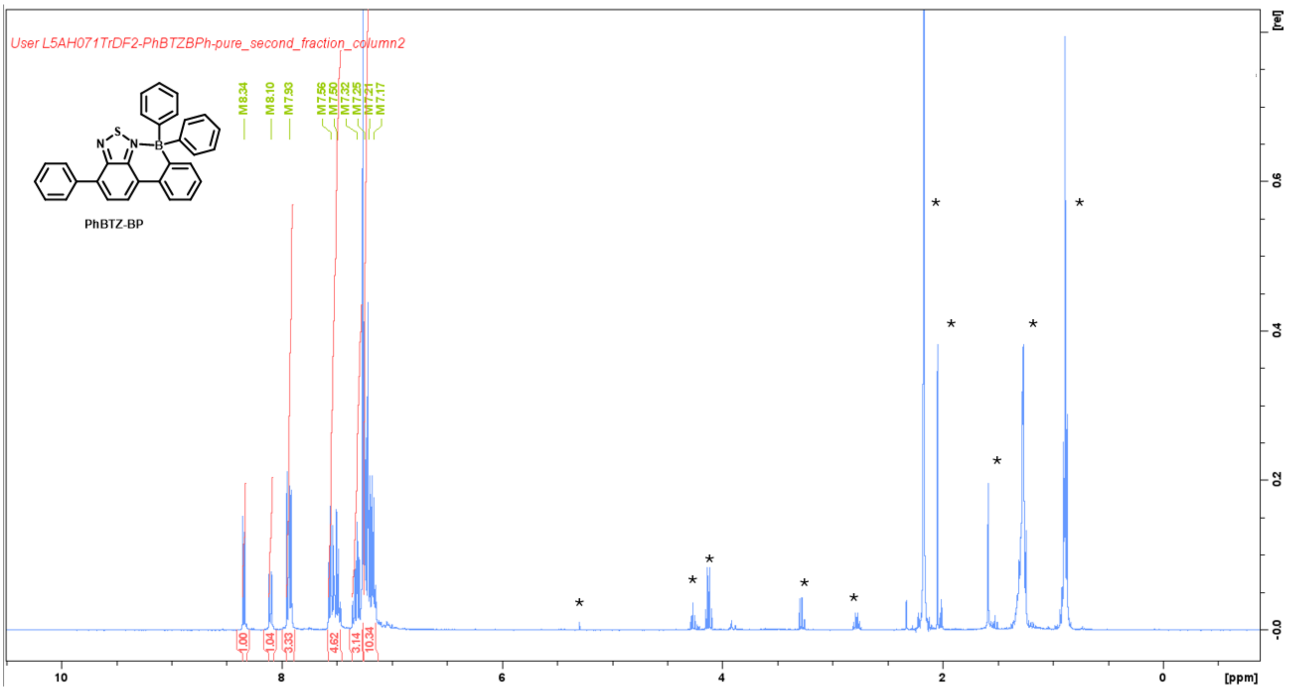

Figure S14. ^1H NMR (400 MHz) spectrum of PhBTZ-BP in $CDCl_3$.

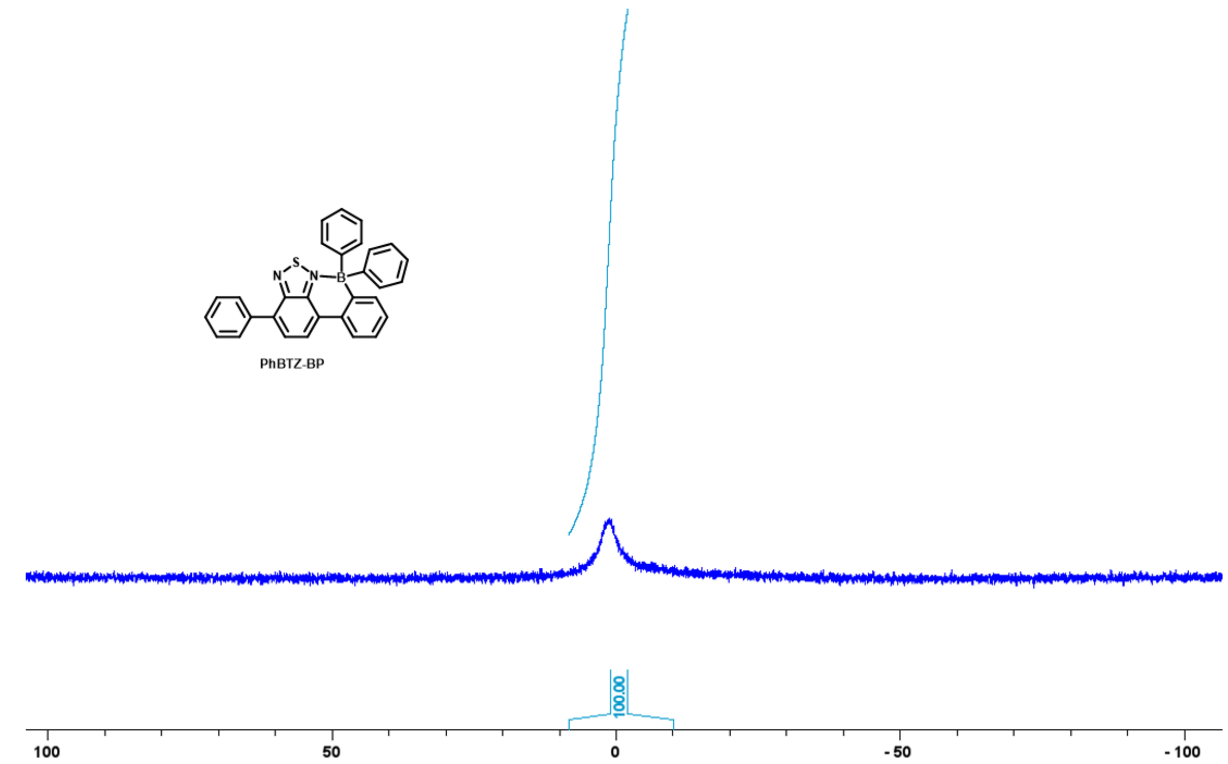

Figure S15. ^{11}B NMR (400 MHz) spectrum of PhBTZ-BP in $CDCl_3$.

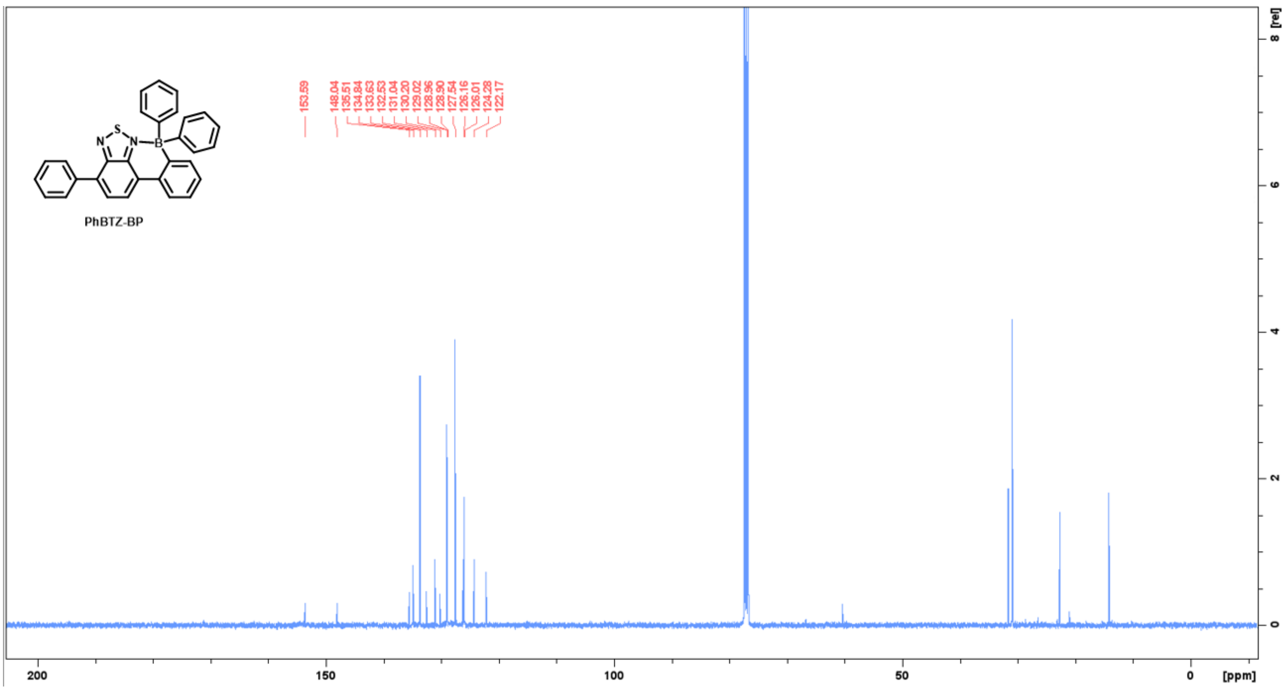

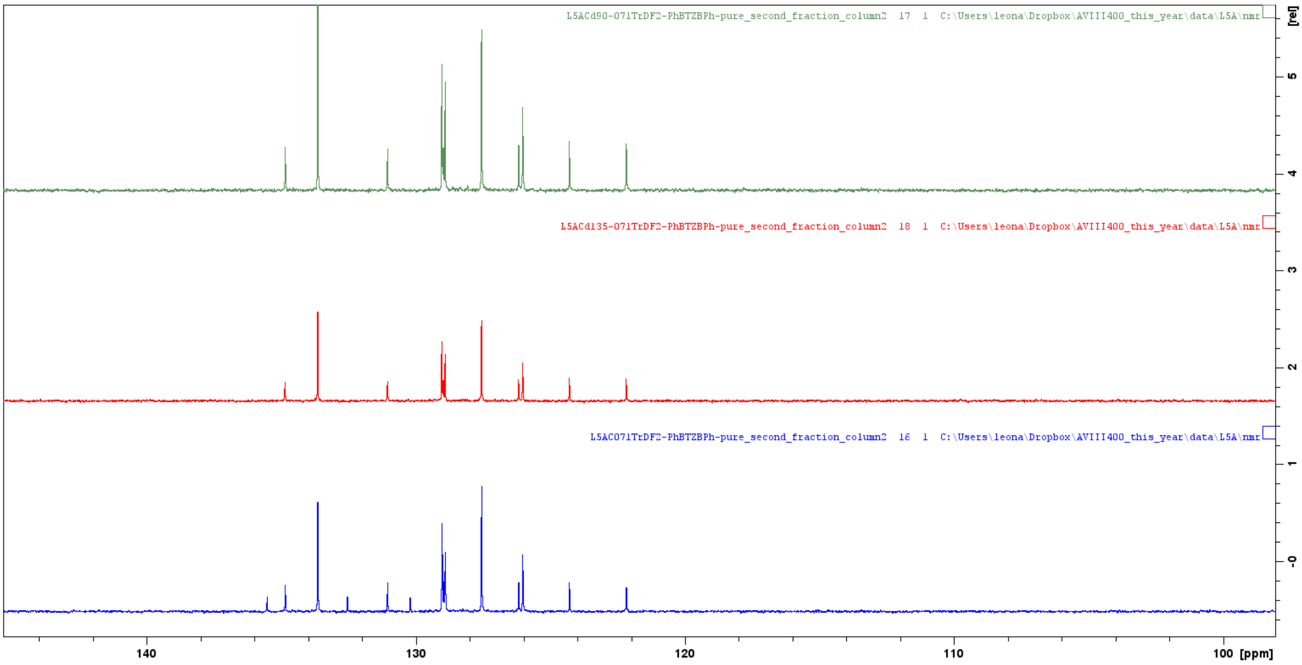

Figure S16. $^{13}C\{^1H\}$ NMR, DEPT90 and DEPT135 spectra of PhBTZ-BP in $CDCl_3$.

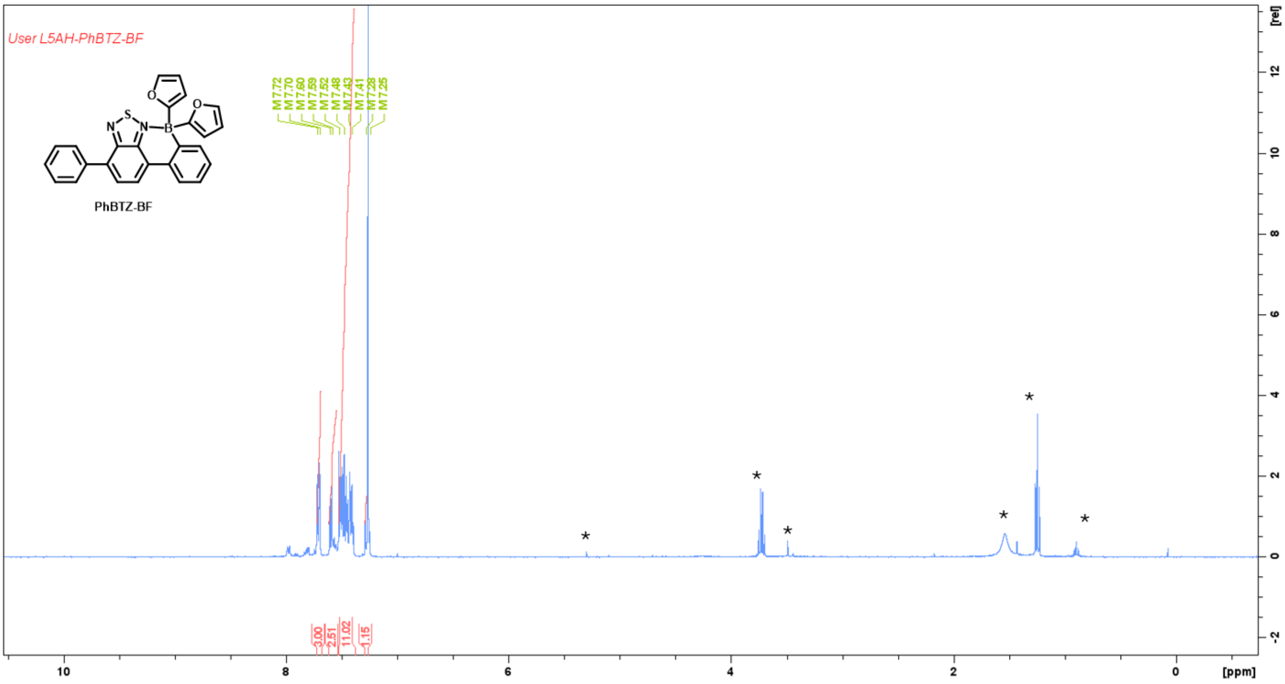

Figure S17. ^1H NMR (400 MHz) spectrum of PhBTZ-BF in $CDCl_3$.

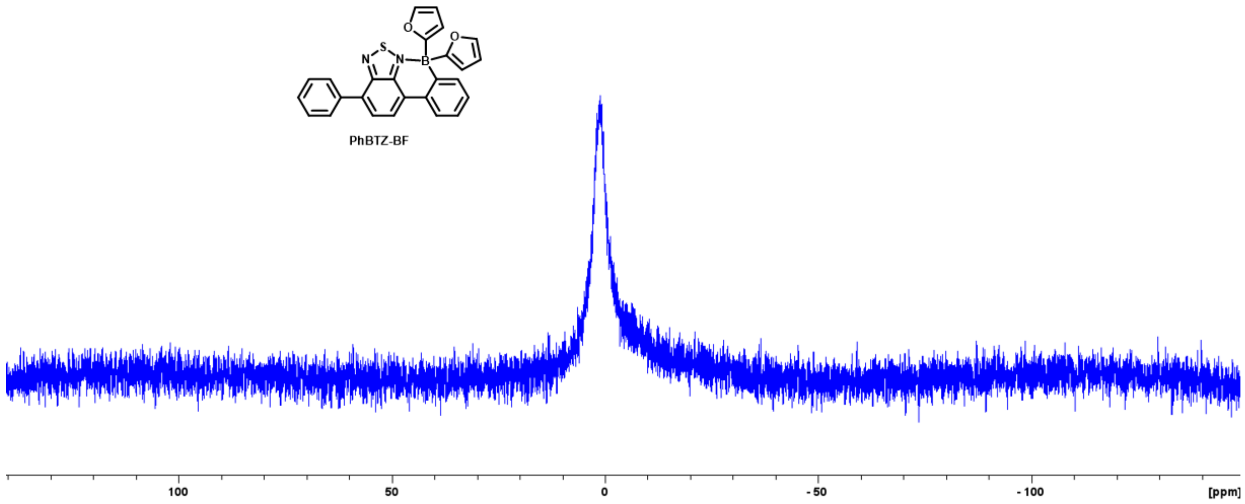

Figure S18. ^{11}B NMR (400 MHz) spectrum of PhBTZ-BF in $CDCl_3$.

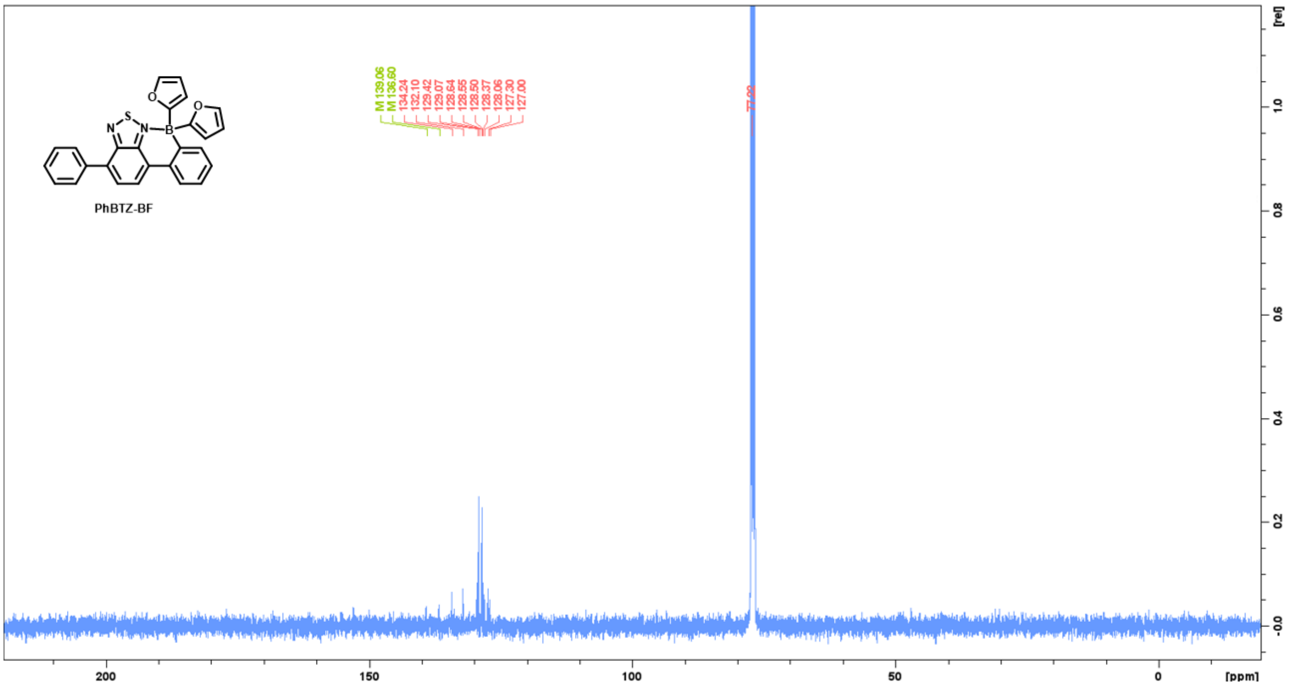

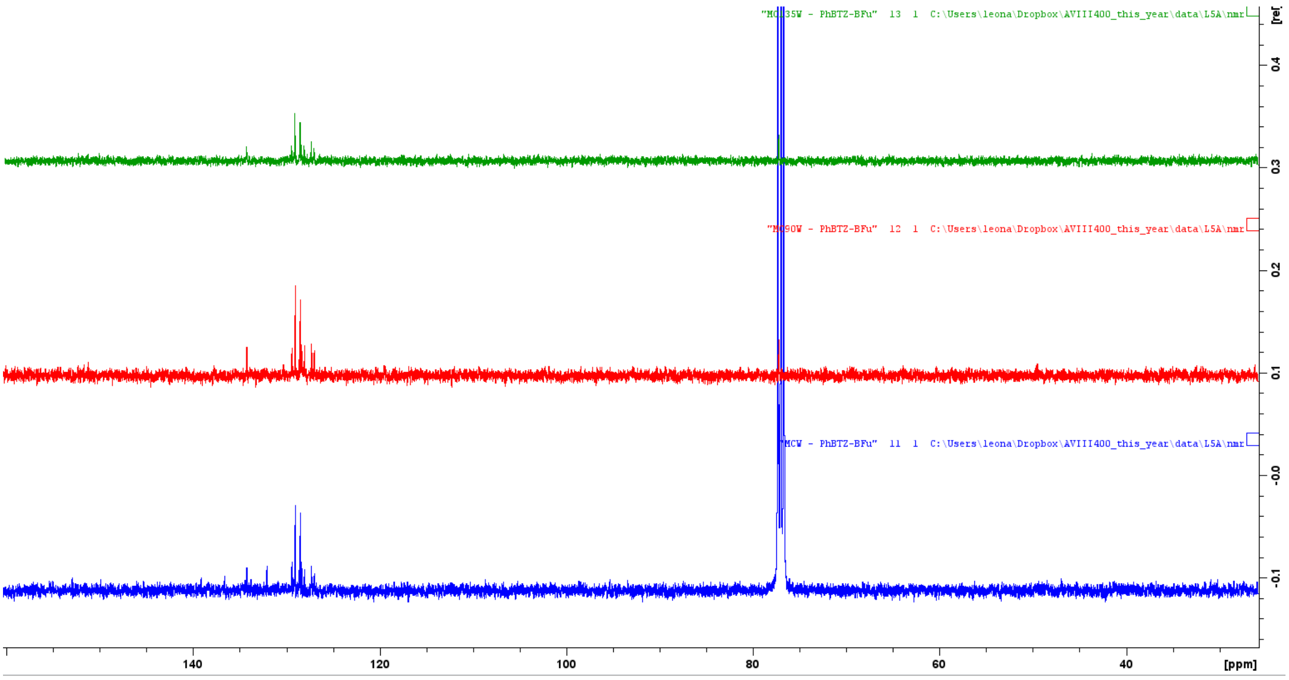

Figure S19. $^{13}C\{^{1}H\}$ NMR, DEPT90 and DEPT135 spectra of PhBTZ-BF in $CDCl_3$.

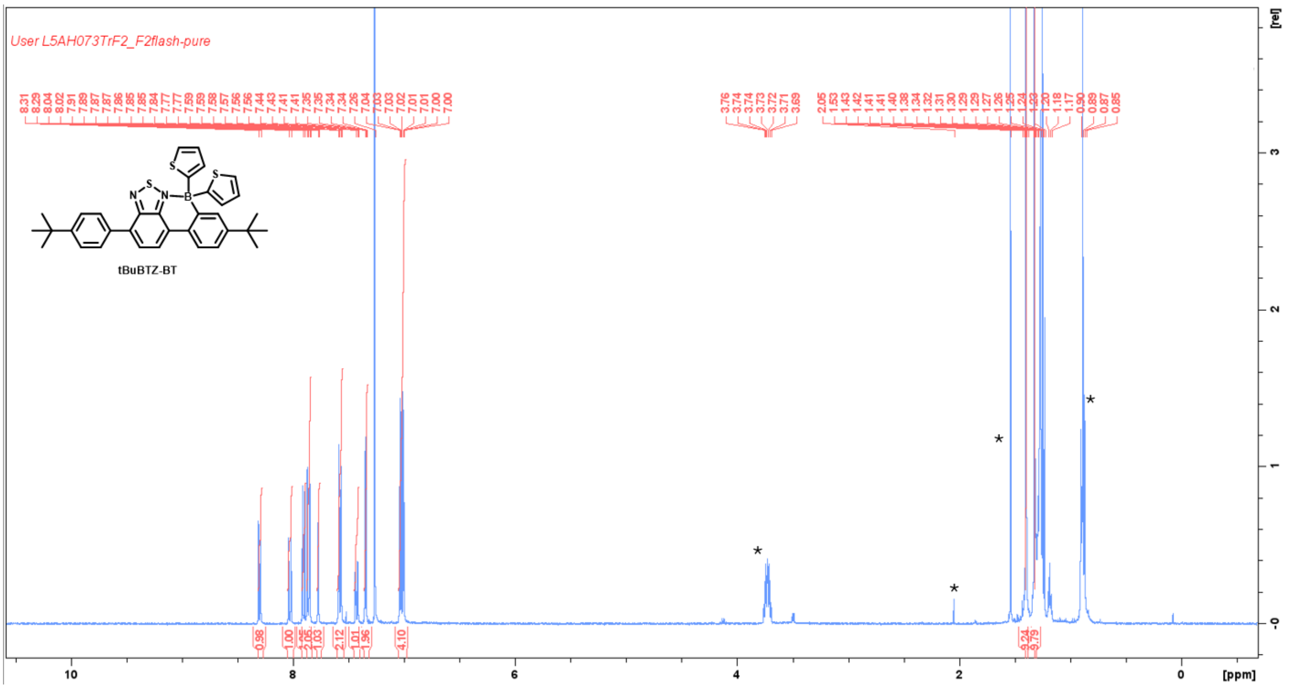

Figure S20. ^{1}H NMR (400 MHz) spectrum of tBuBTZ-BT in $CDCl_3$.

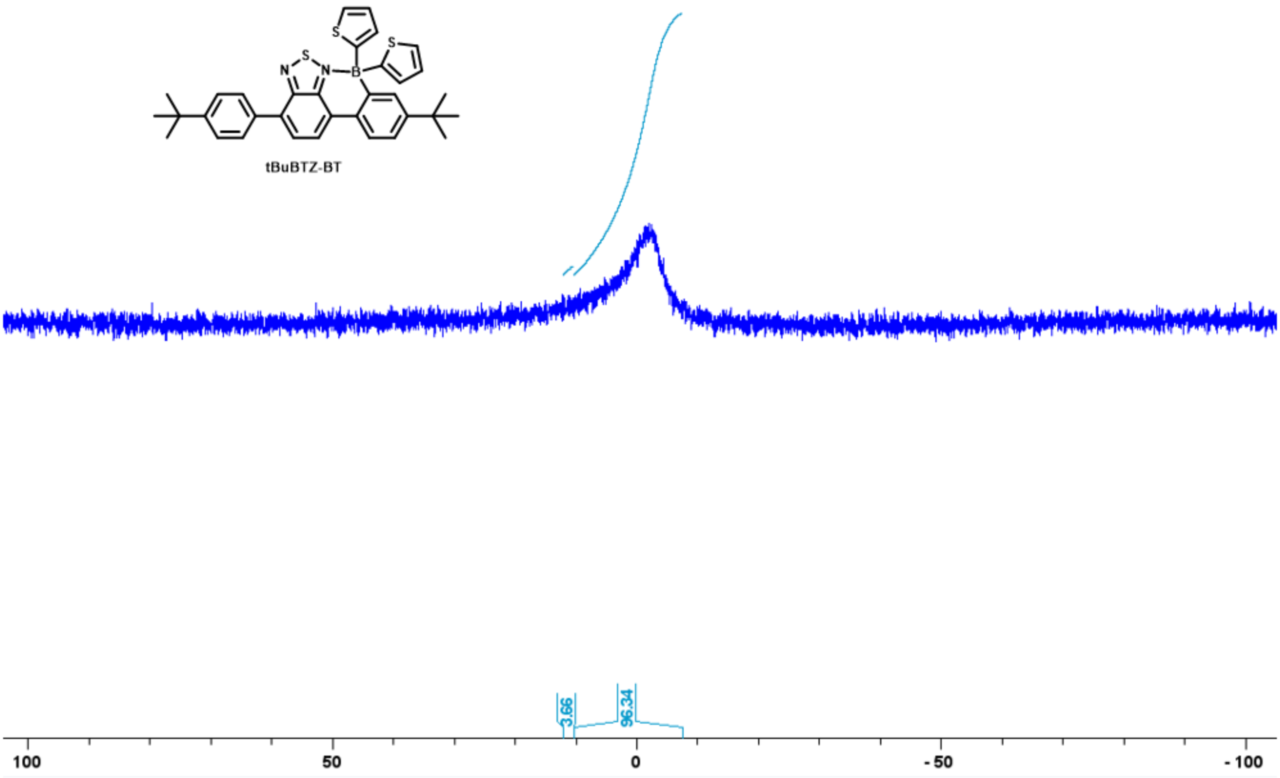

Figure S21. ^{11}B NMR (400 MHz) spectrum of tBuBTZ-BT in $CDCl_3$.

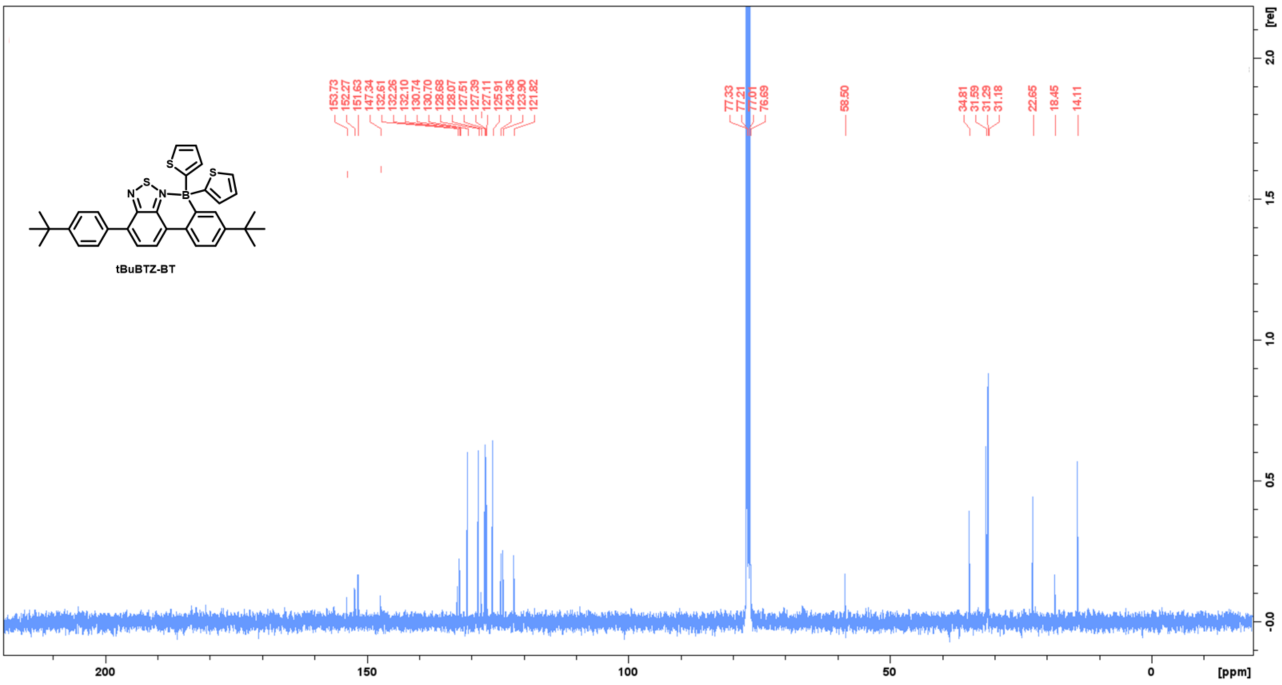

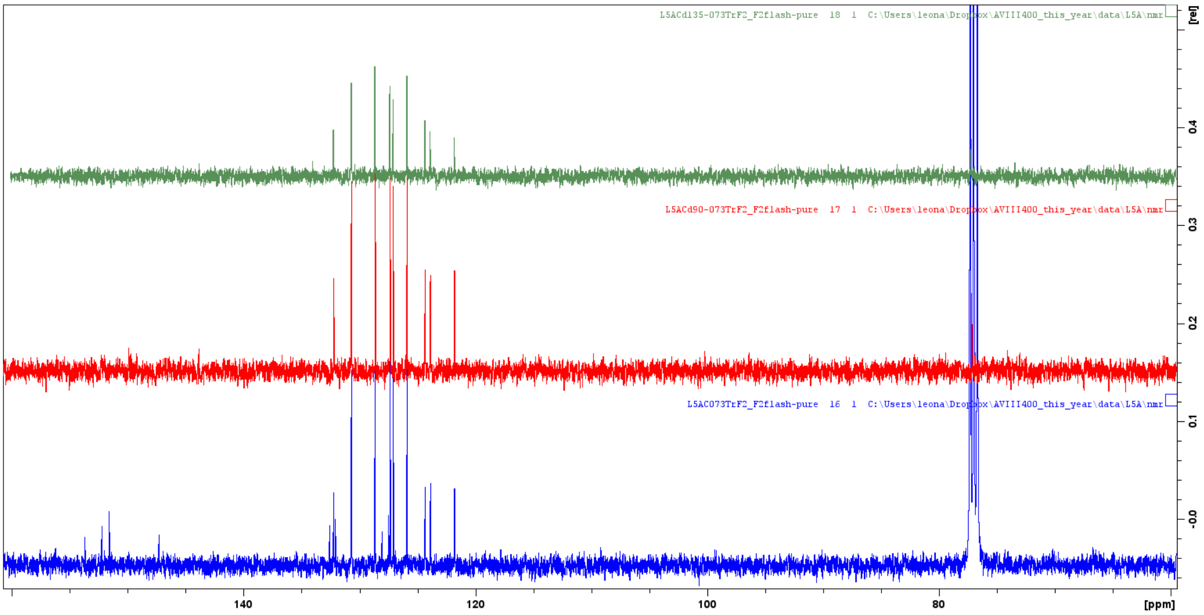

Figure S22. $^{13}C\{^1H\}$ NMR, DEPT90 and DEPT135 spectra of tBuBTZ-BT in $CDCl_3$.

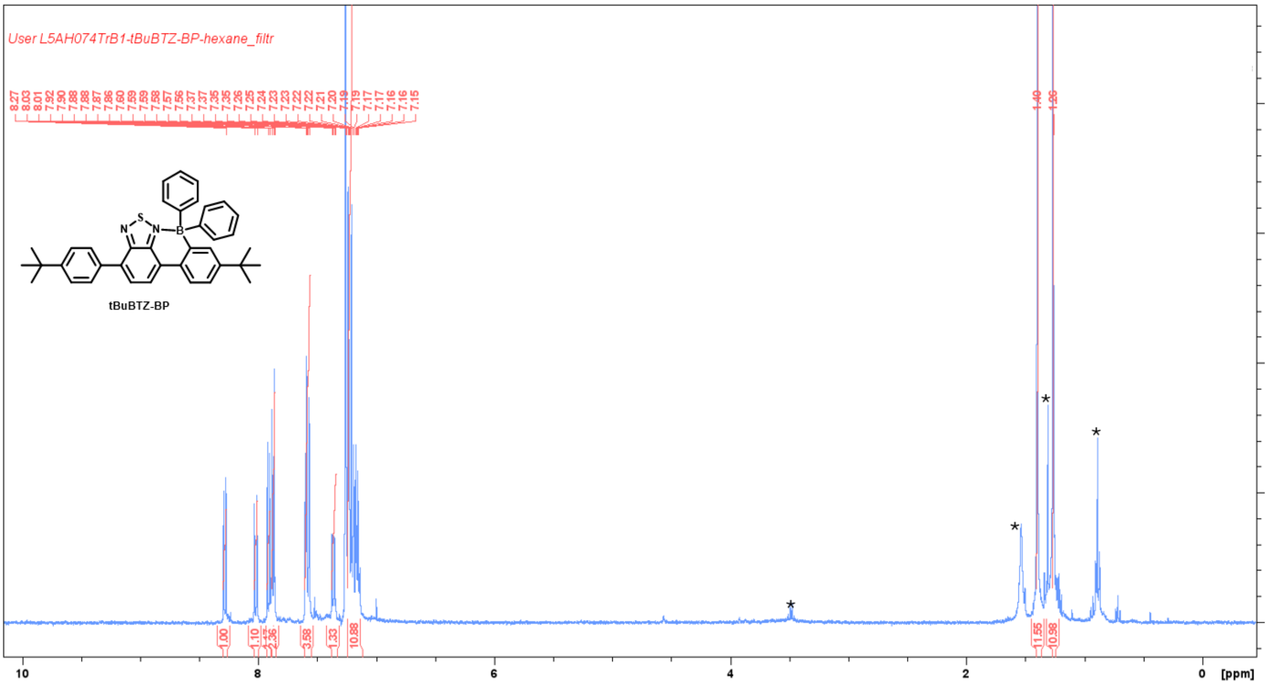

Figure S23. ^1H NMR (400 MHz) spectrum of tBuBTZ-BP in $CDCl_3$.

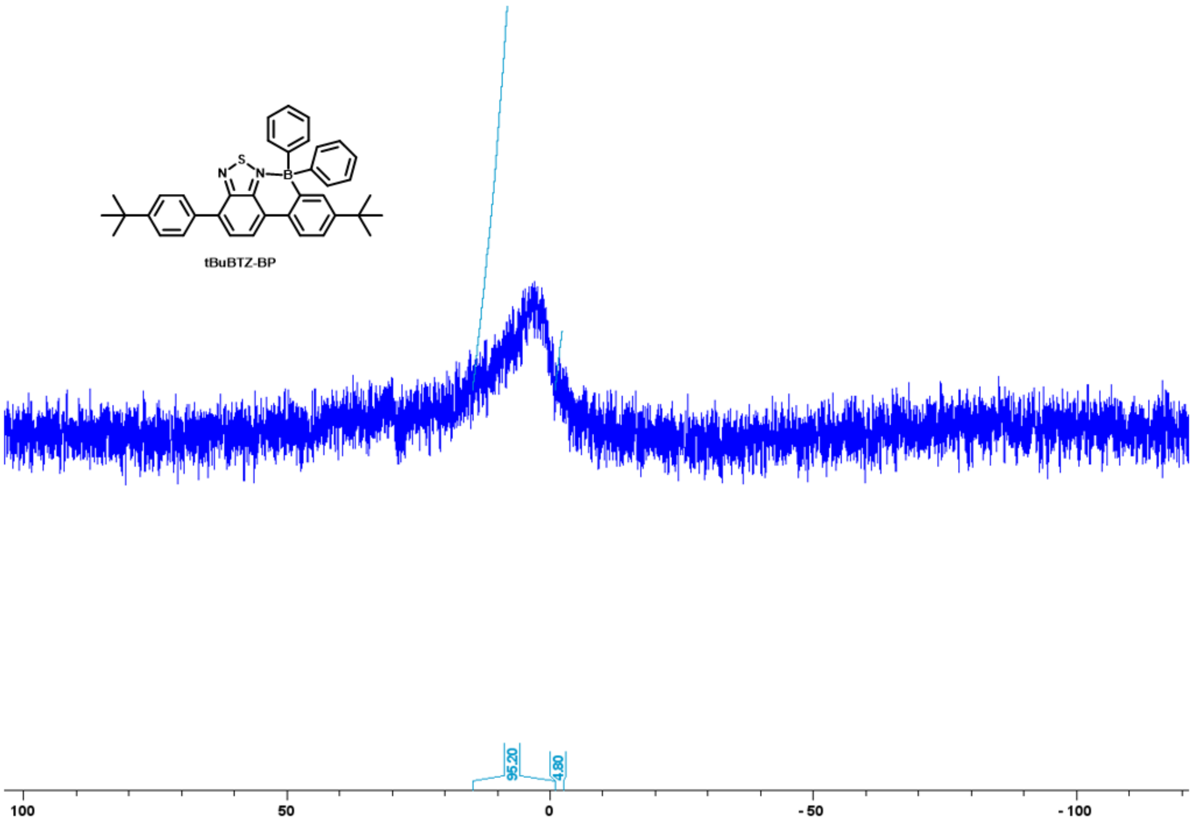

Figure S24. ^{11}B NMR (400 MHz) spectrum of tBuBTZ-BP in $CDCl_3$.

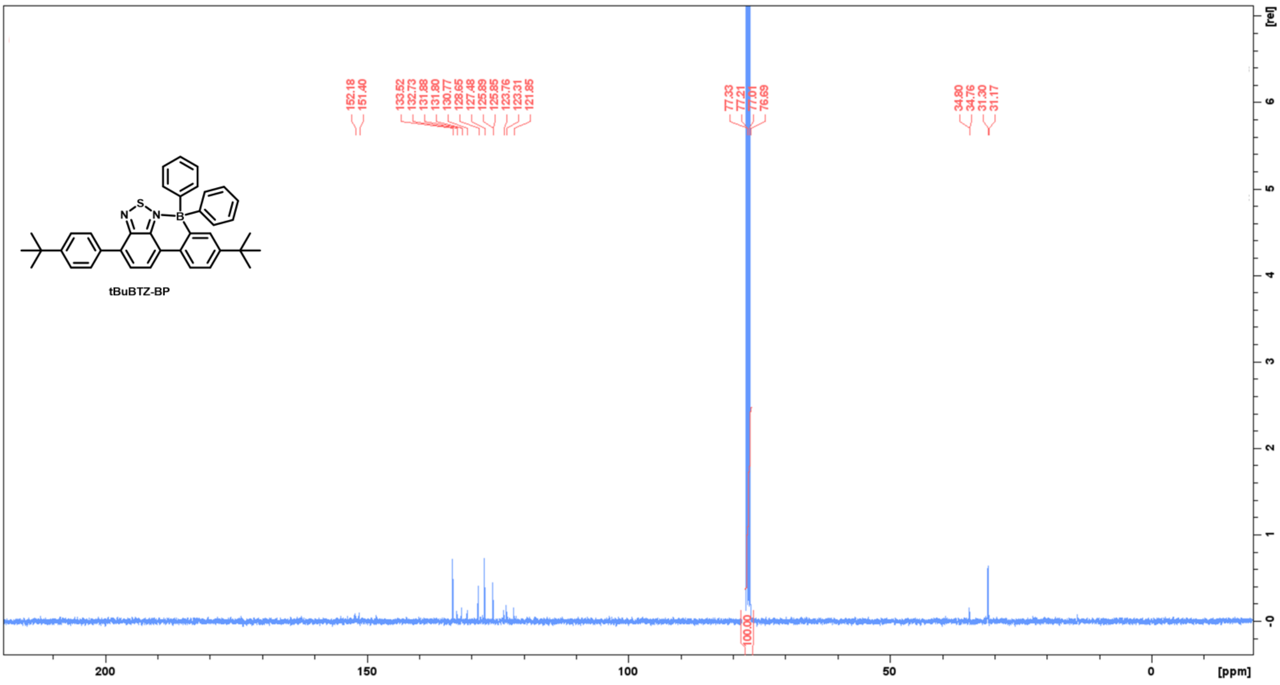

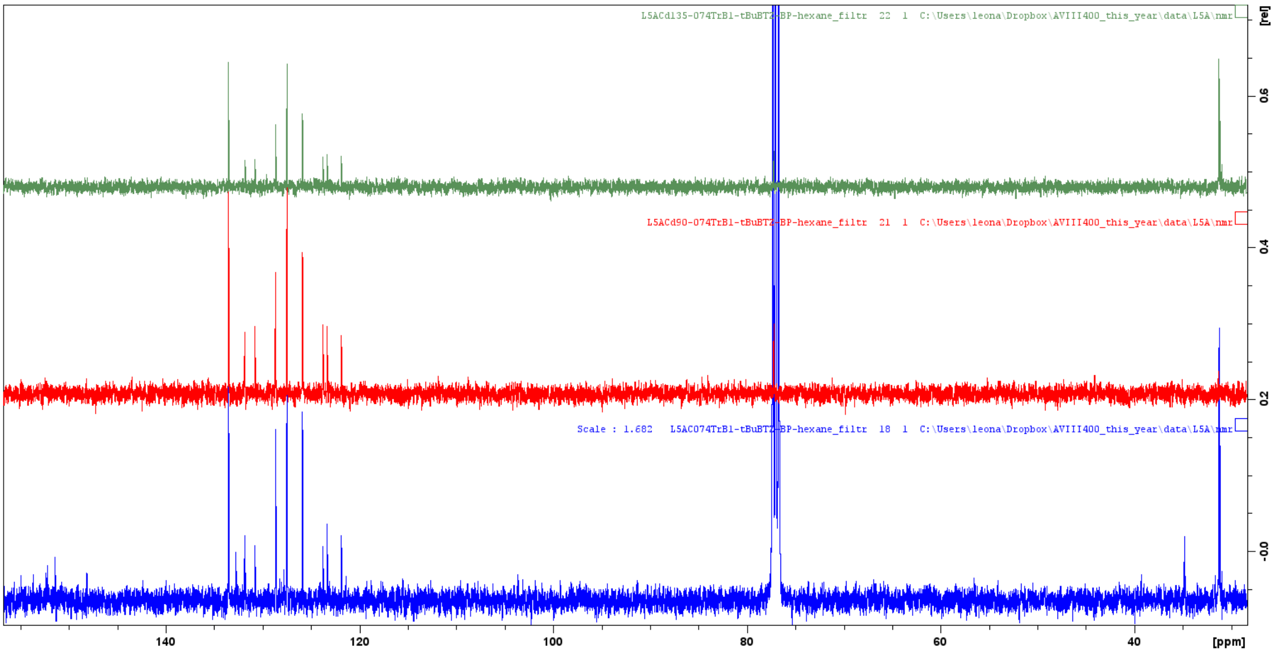

Figure S25. $^{13}C\{^1H\}$ NMR, DEPT90 and DEPT135 spectra of tBuBTZ-BP in $CDCl_3$.

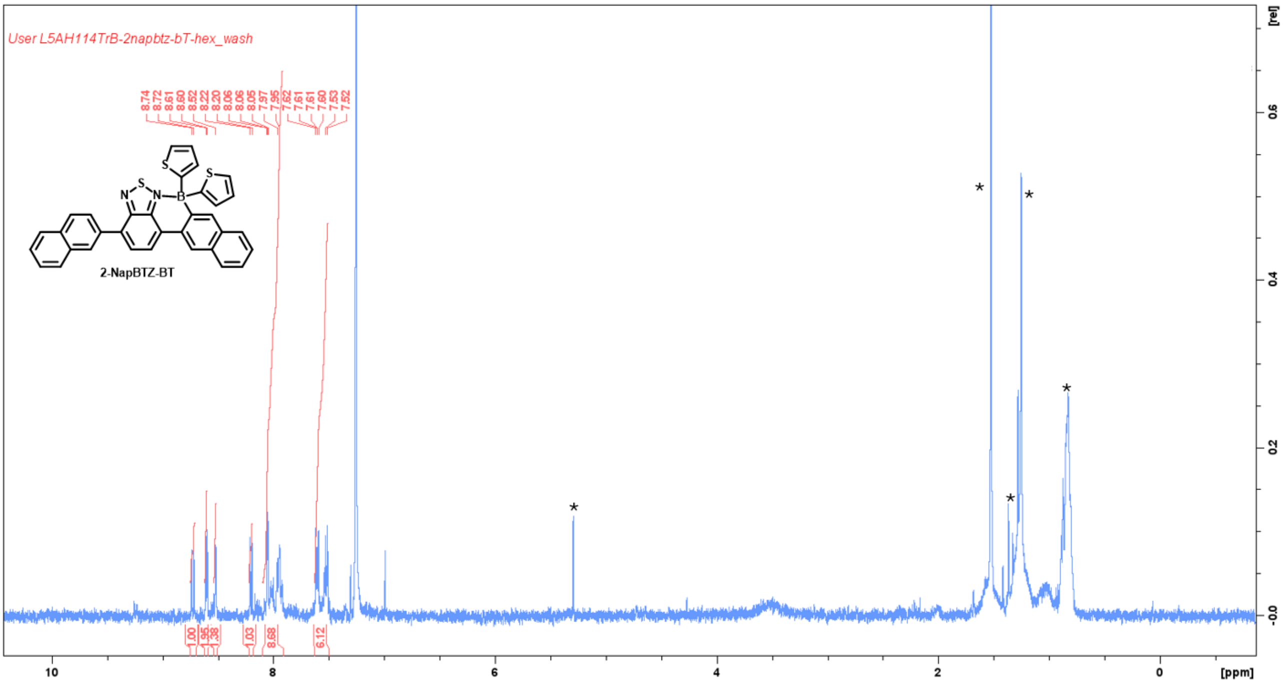

Figure S26. ^1H NMR (400 MHz) spectrum of 2NapBTZ-BT in $CDCl_3$.

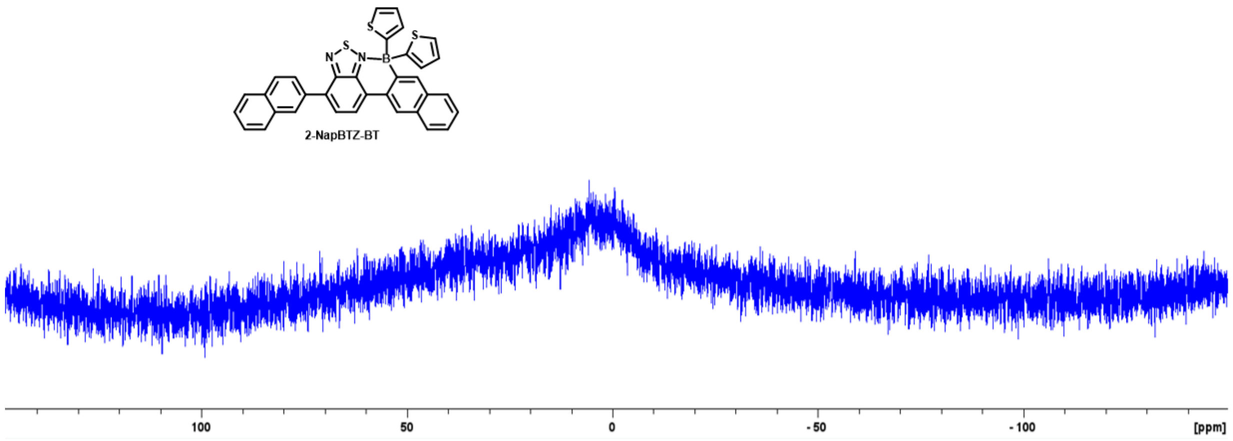

Figure S27. ^{11}B NMR (400 MHz) spectrum of 2NapBTZ-BT in $CDCl_3$.

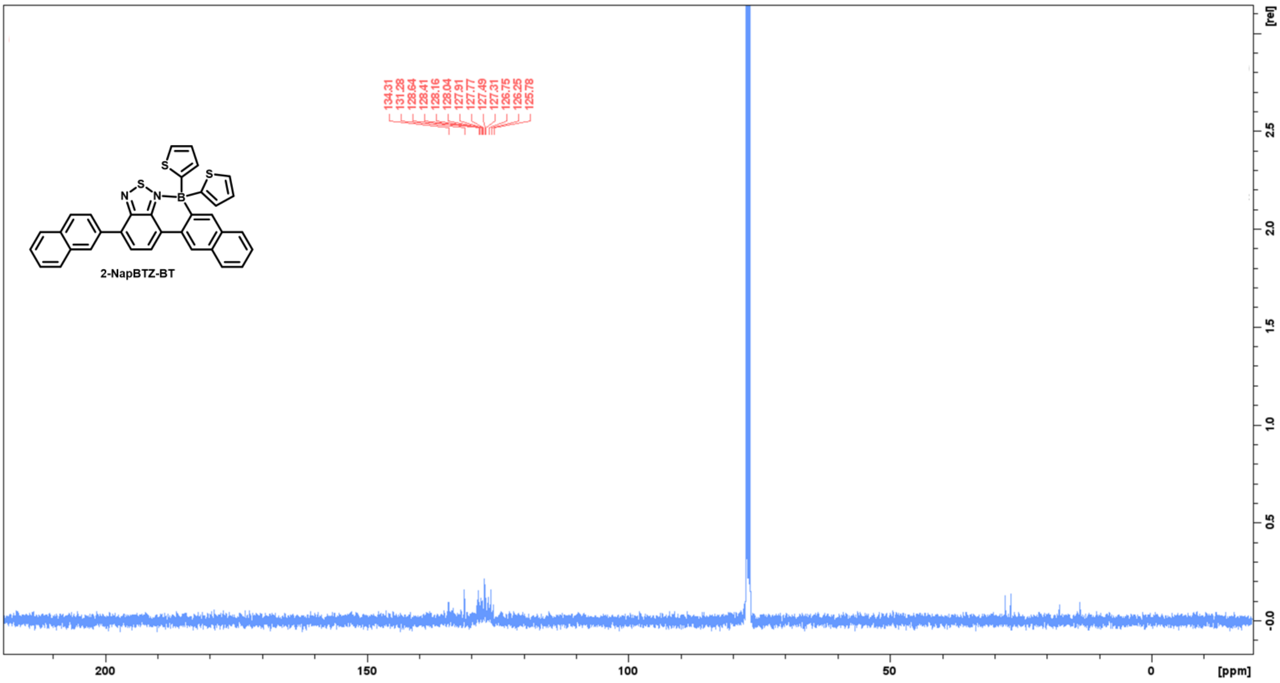

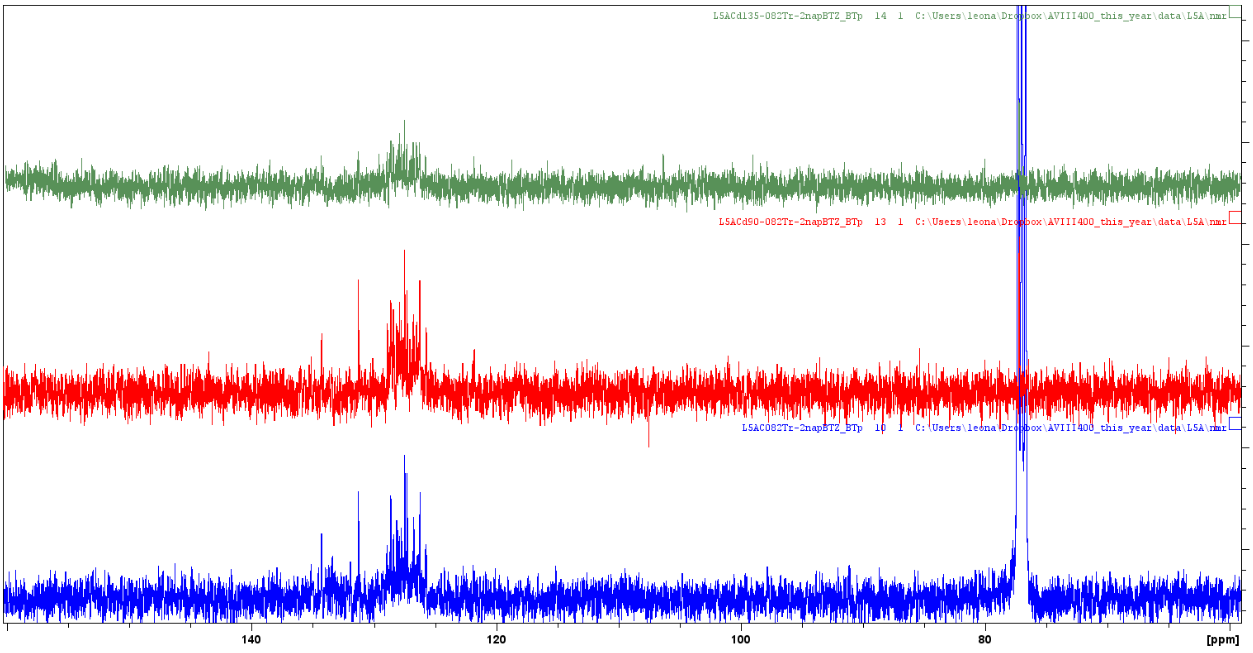

Figure S28. $^{13}C\{^1H\}$ NMR, DEPT90 and DEPT135 spectra of 2NapBTZ-BT in $CDCl_3$.

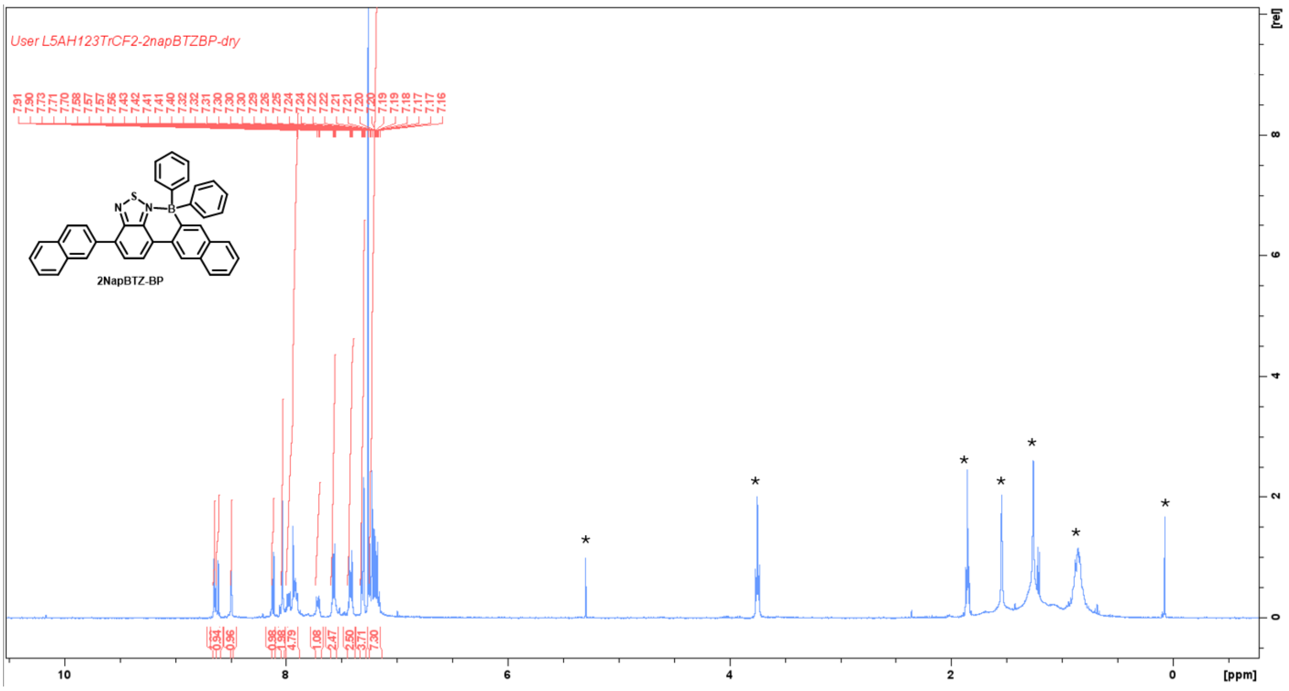

Figure S29. ^1H NMR (400 MHz) spectrum of 2NapBTZ-BP in $CDCl_3$.

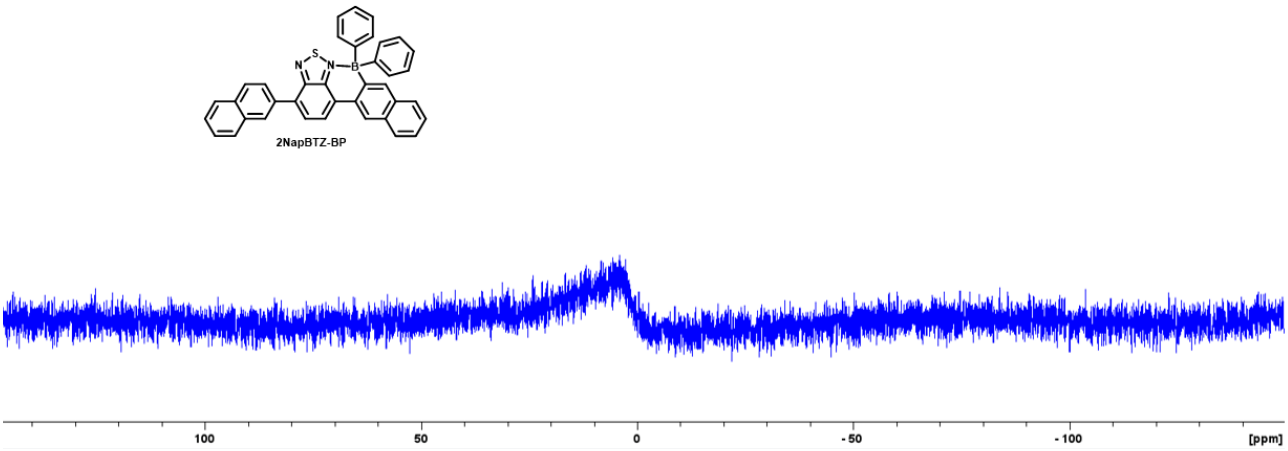

Figure S30. ^{11}B NMR (400 MHz) spectrum of 2NapBTZ-BP in $CDCl_3$.

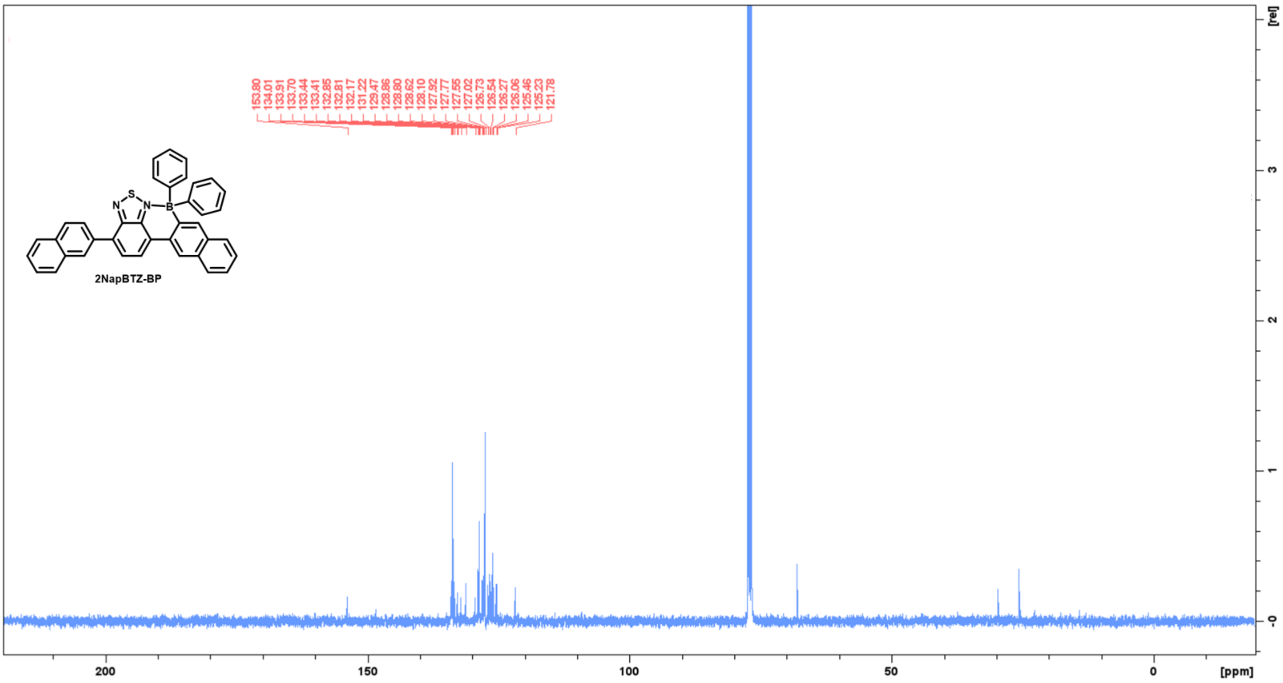

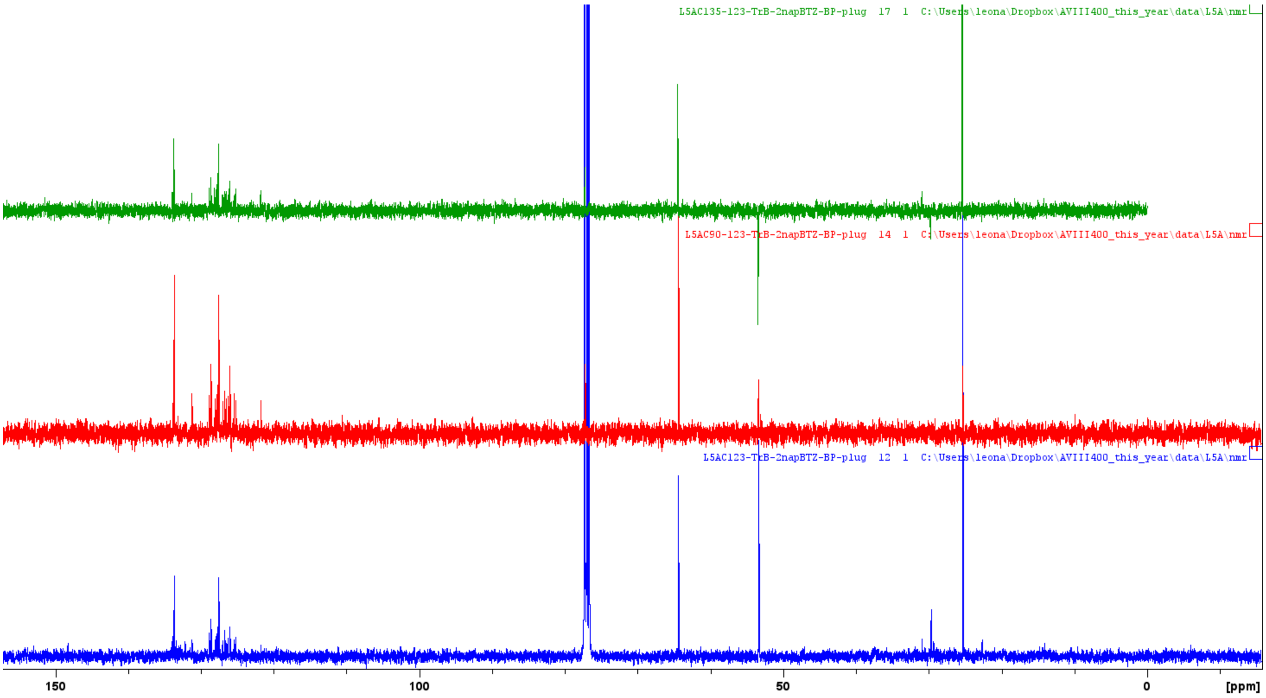

Figure S31. $^{13}C\{^{1}H\}$ NMR, DEPT90 and DEPT135 spectra of 2NapBTZ-BP in $CDCl_3$.

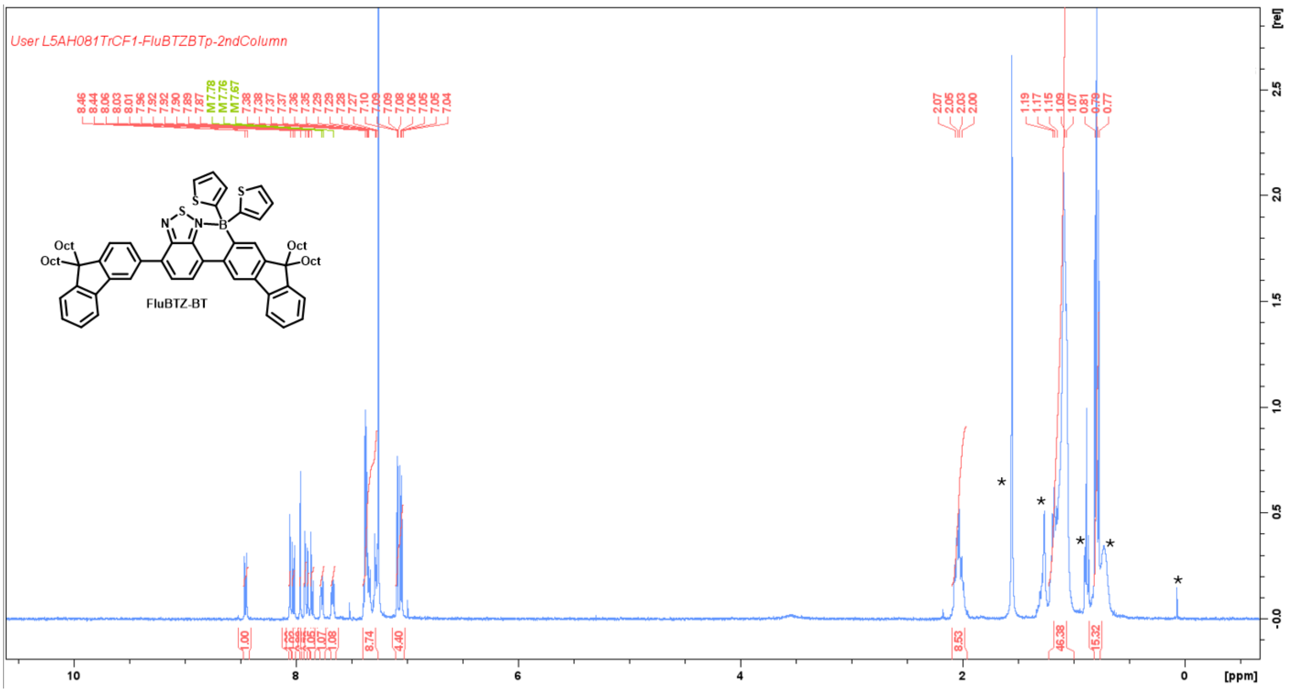

Figure S32. ^{1}H NMR (400 MHz) spectrum of FluBTZ-BT in $CDCl_3$.

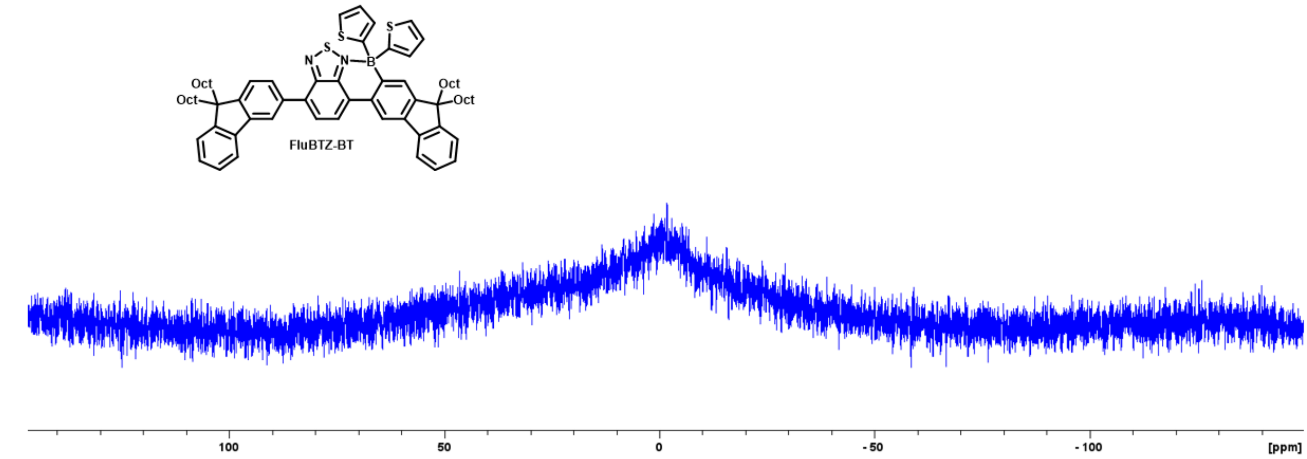

Figure S33. ^{11}B NMR (400 MHz) spectrum of FluBTZ-BT in $CDCl_3$.

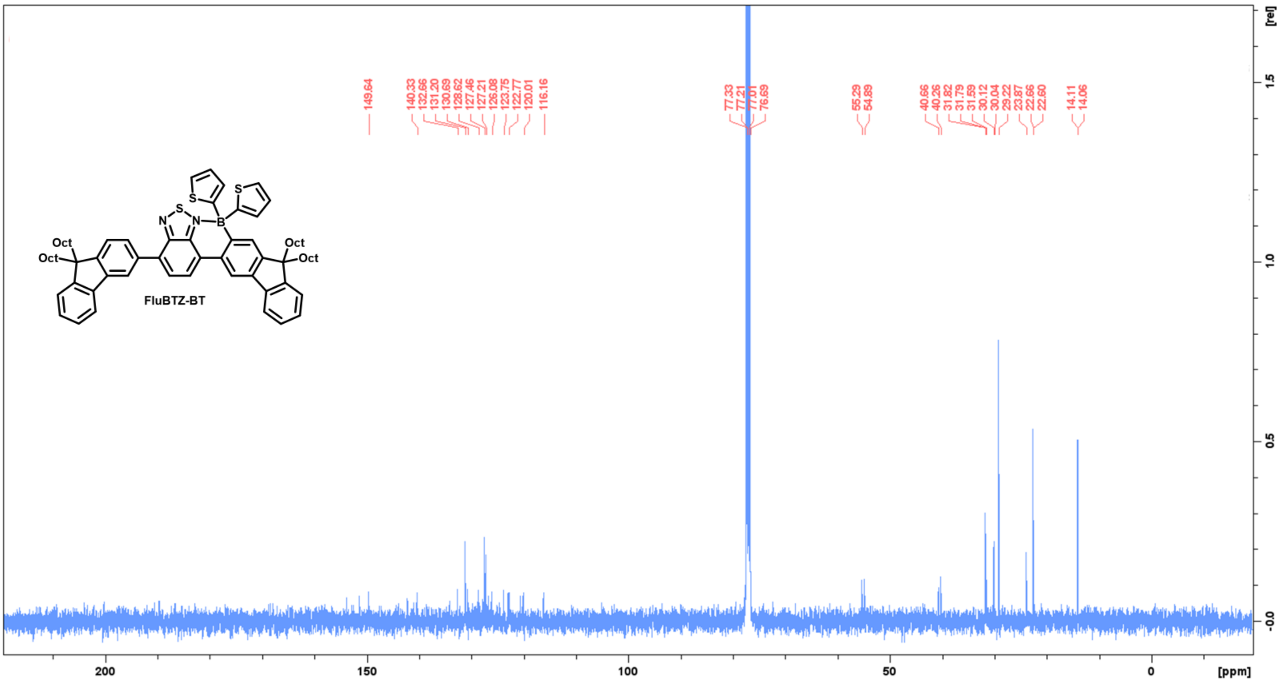

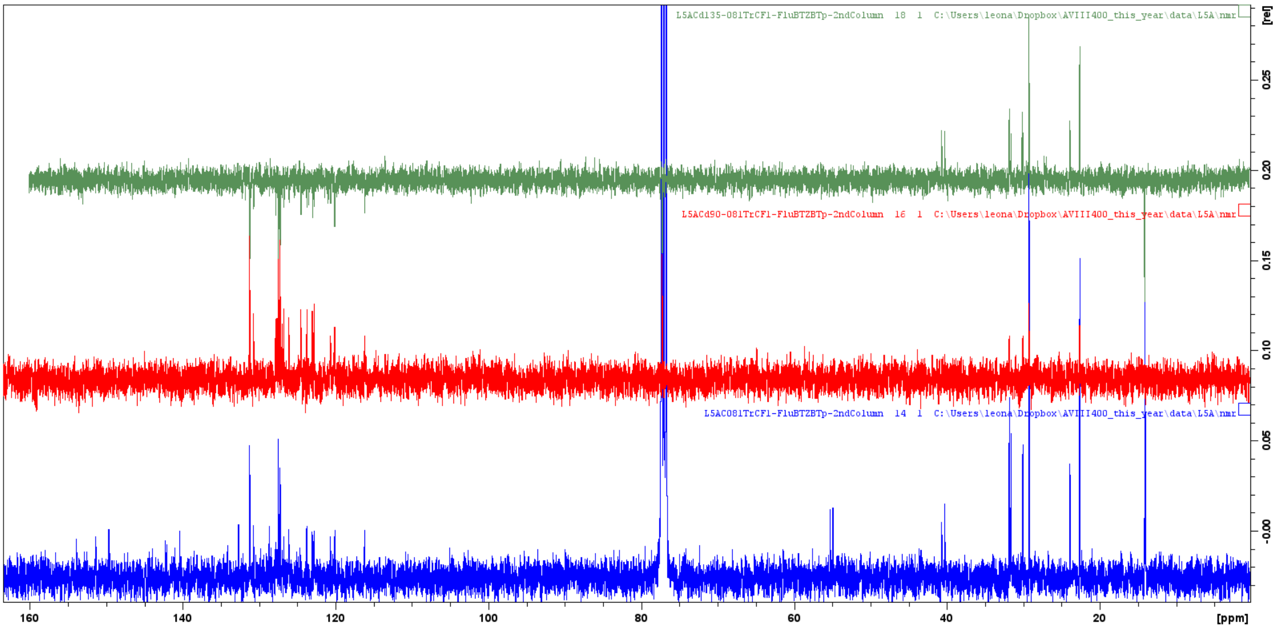

Figure S34. $^{13}C\{^1H\}$ NMR, DEPT90 and DEPT135 spectra of FluBTZ-BT in $CDCl_3$.

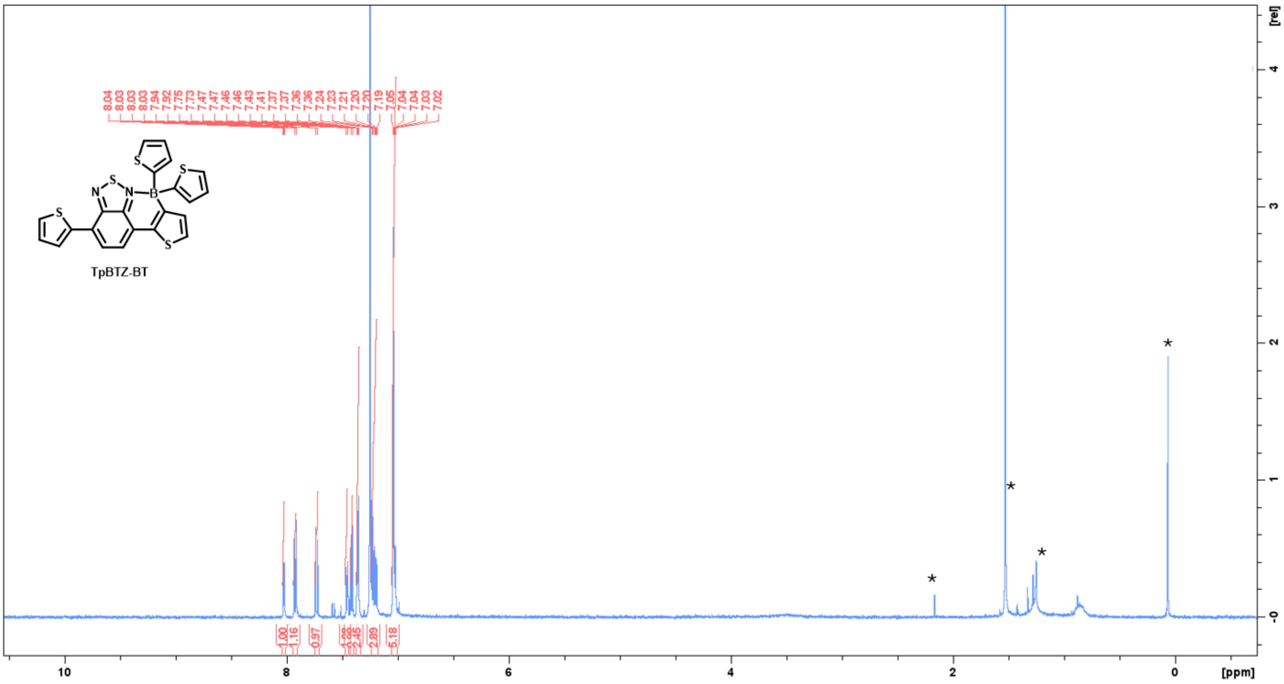

Figure S35. ^1H NMR (400 MHz) spectrum of TpBTZ-BT in $CDCl_3$.

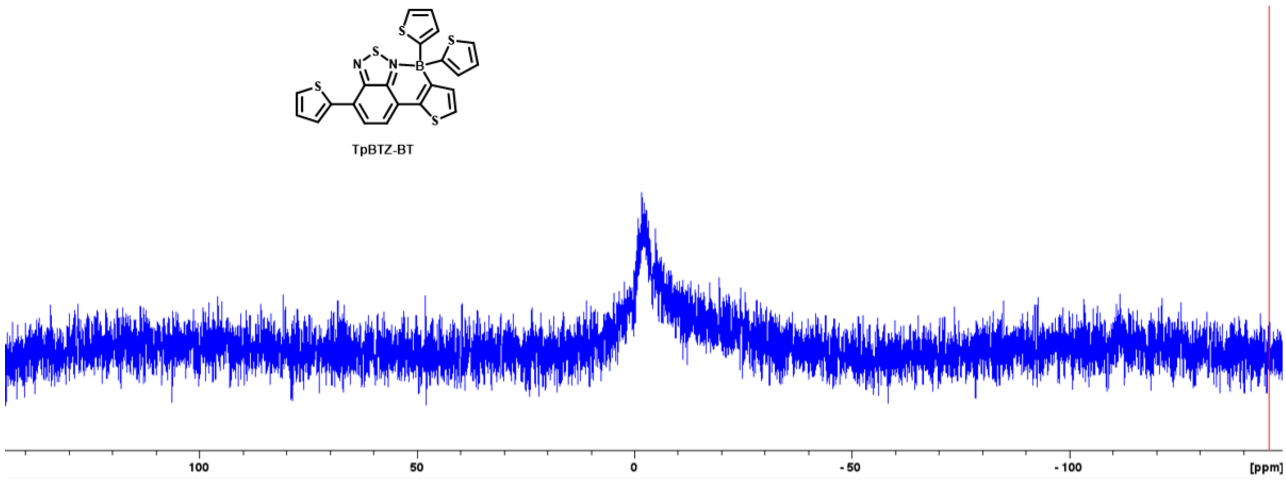

Figure S36. ^{11}B NMR (400 MHz) spectrum of TpBTZ-BT in $CDCl_3$.

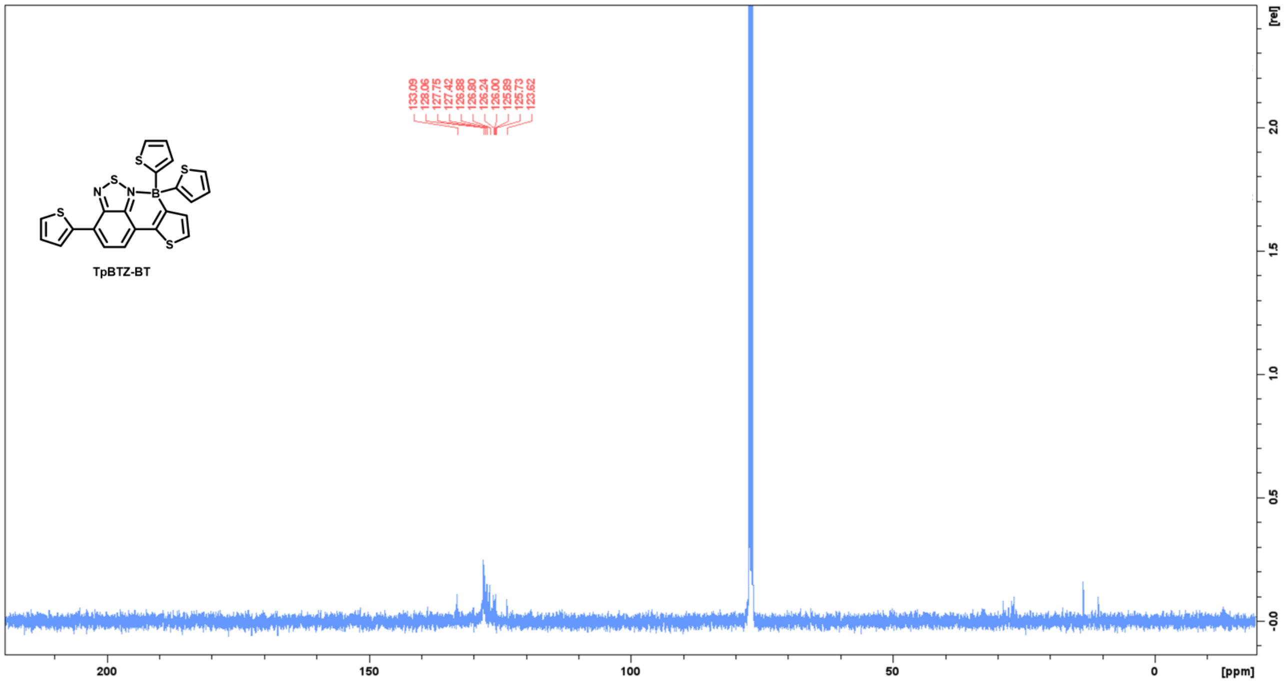

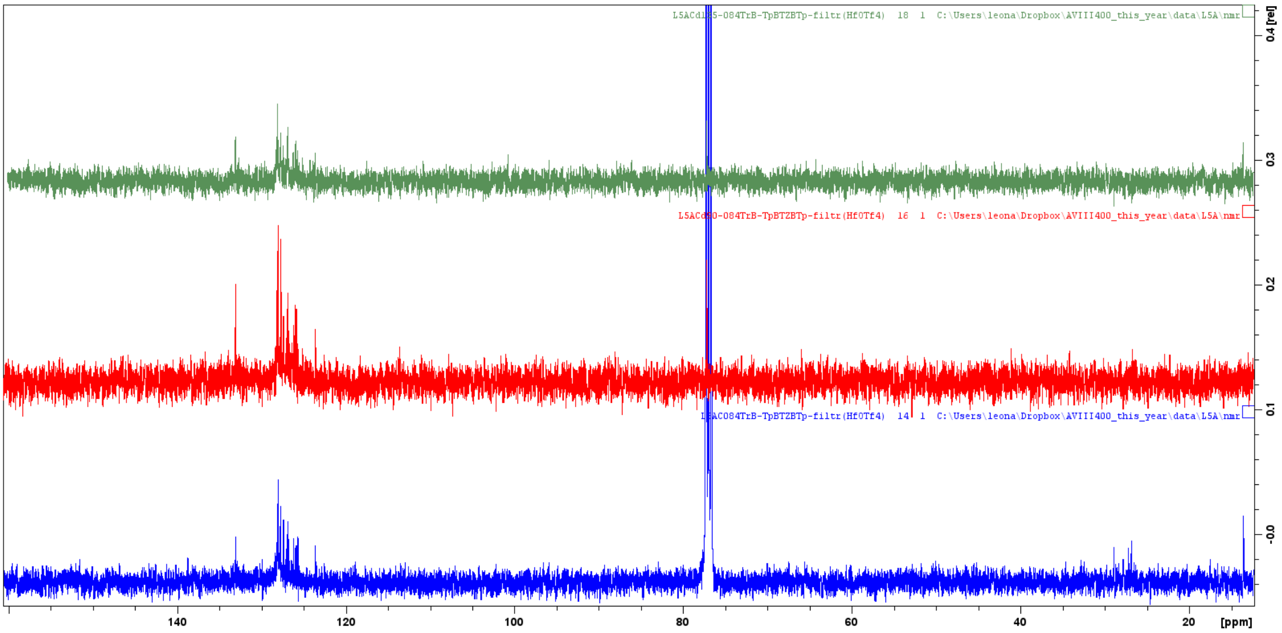

Figure S37. $^{13}C\{1H\}$ NMR, DEPT90 and DEPT135 spectra of TpBTZ-BT in $CDCl_3$.

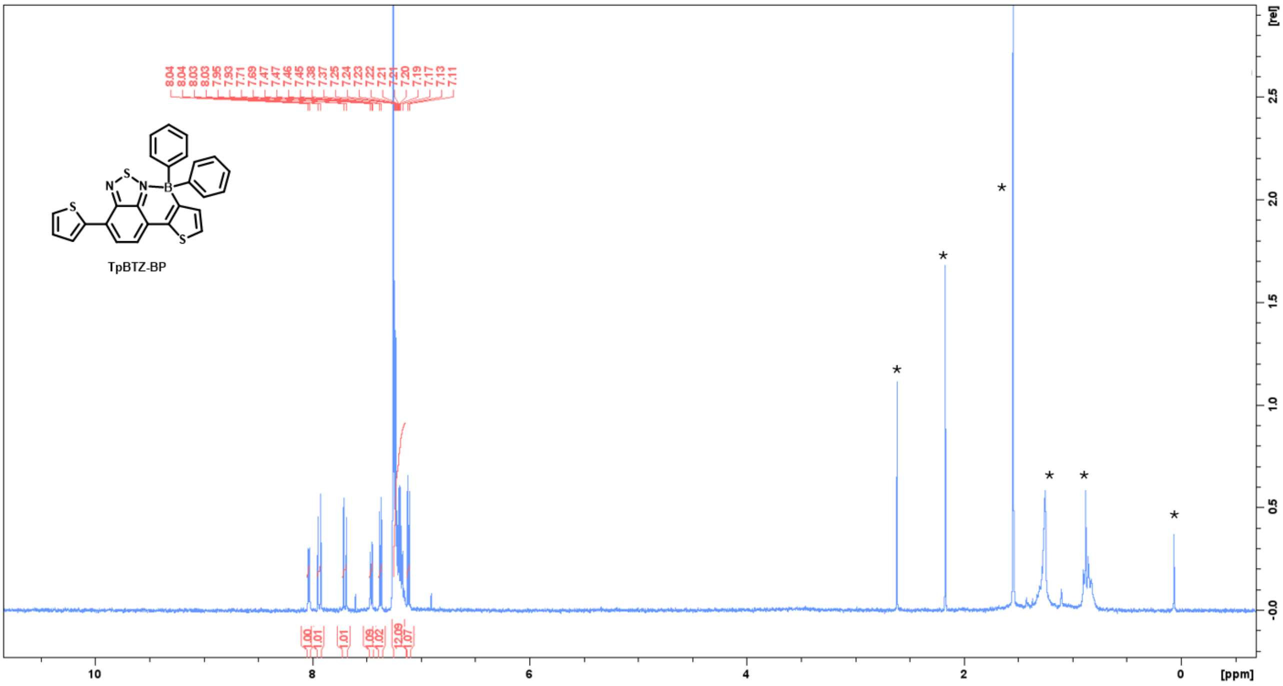

Figure S38. ^{1}H NMR (400 MHz) spectrum of TpBTZ-BP in $CDCl_3$.

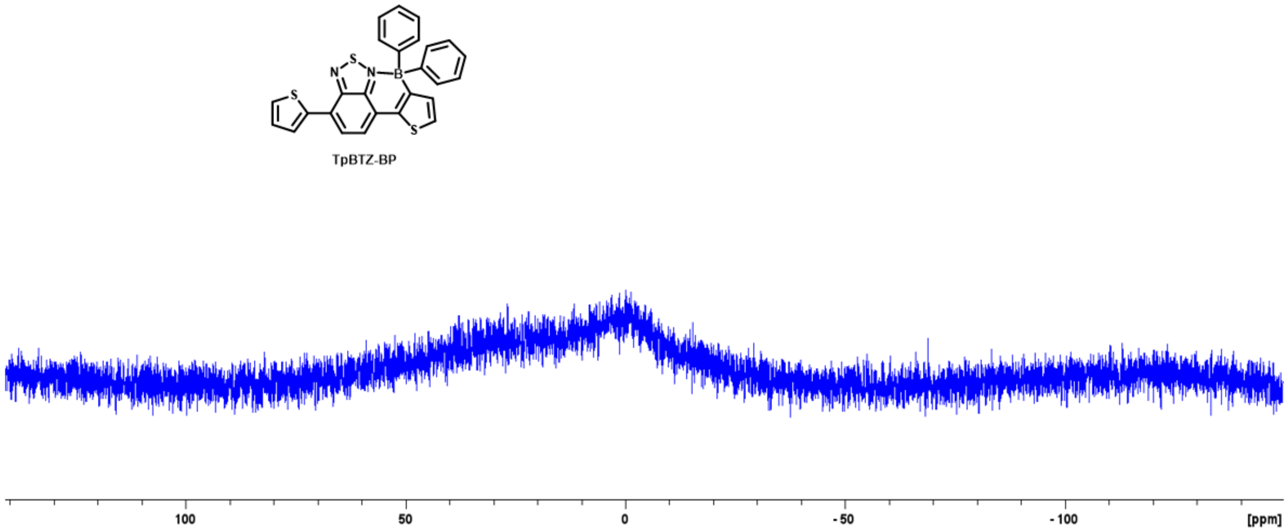

Figure S39. ^{11}B NMR (400 MHz) spectrum of TpBTZ-BP in $CDCl_3$.

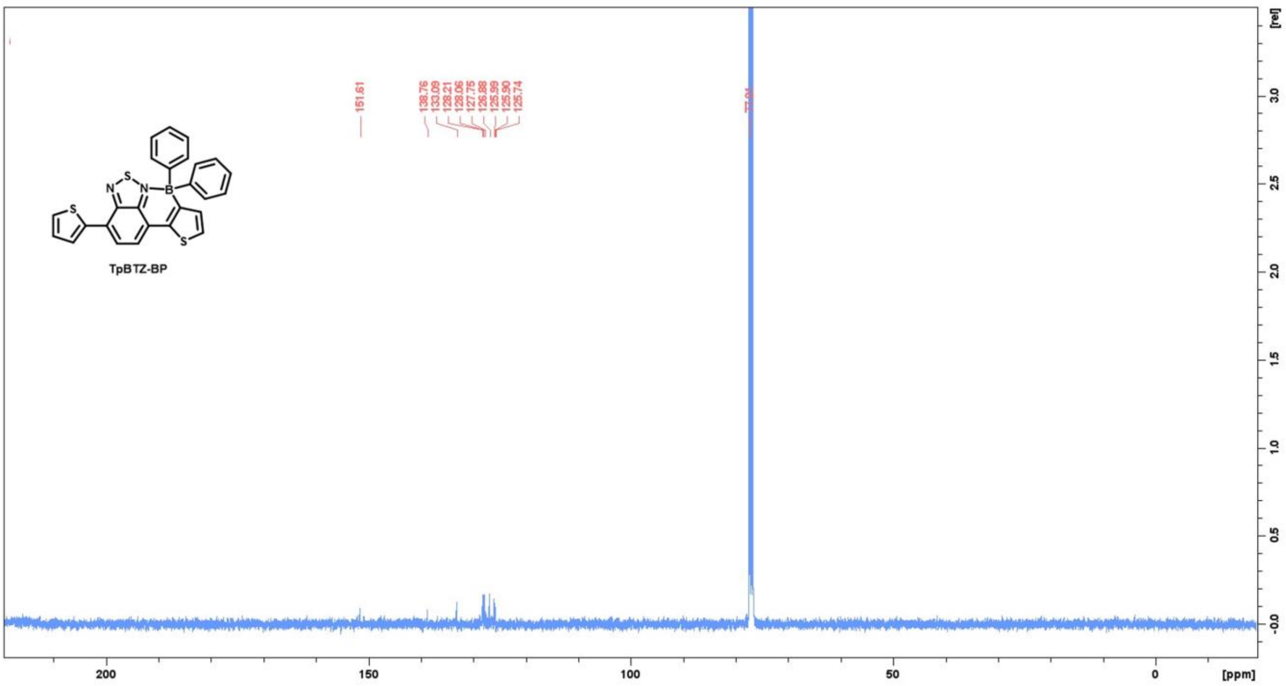

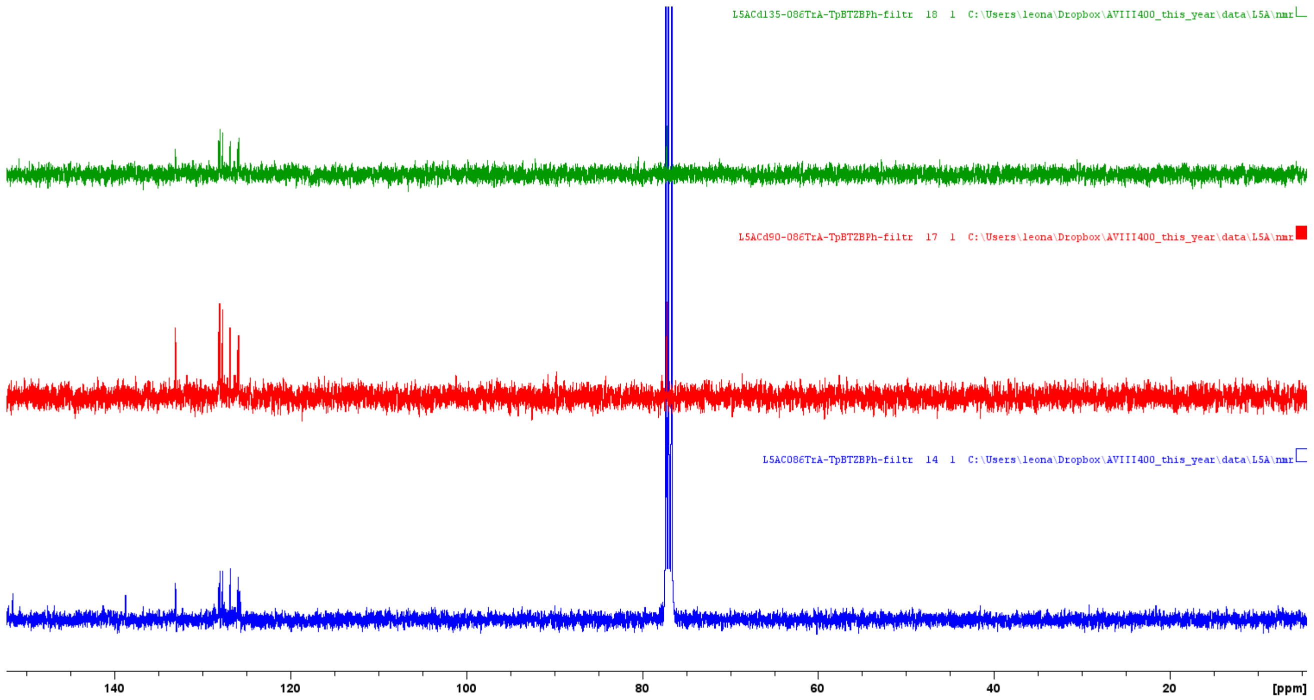

Figure S40. $^{13}C\{^1H\}$ NMR, DEPT90 and DEPT135 spectra of TpBTZ-BP in $CDCl_3$.

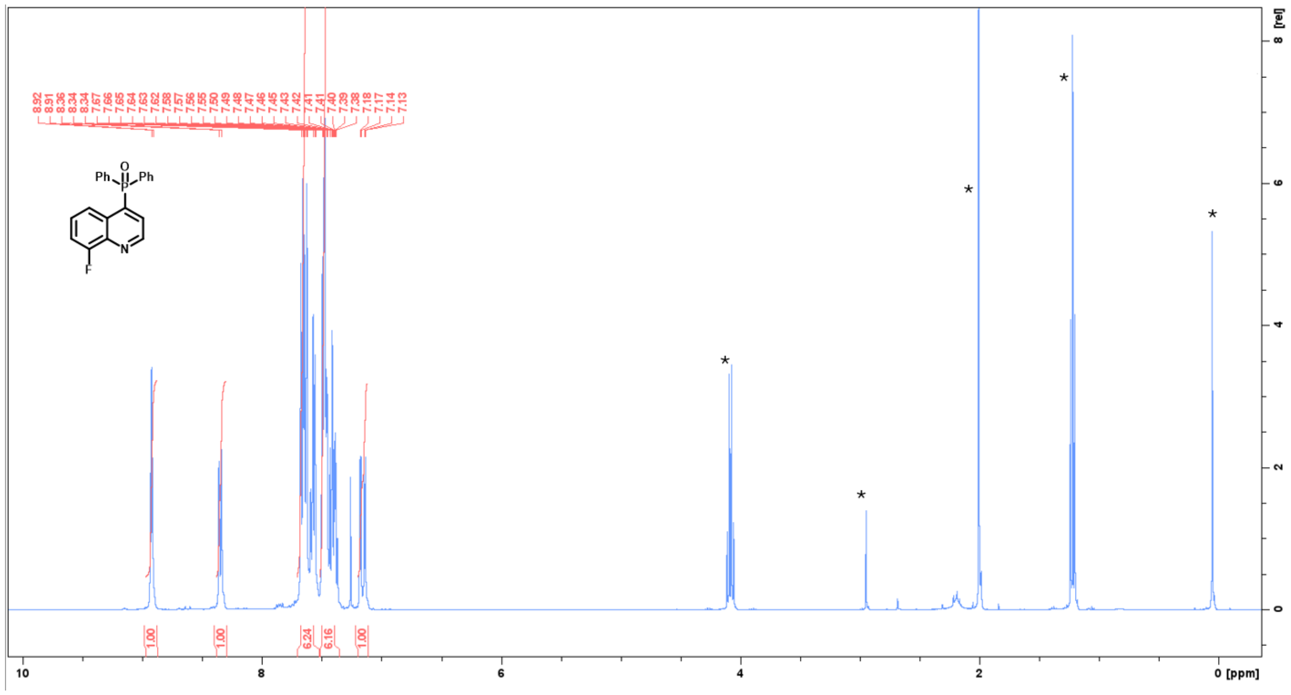

Figure S41. ^1H NMR (400 MHz) spectrum of Q-P in $CDCl_3$.

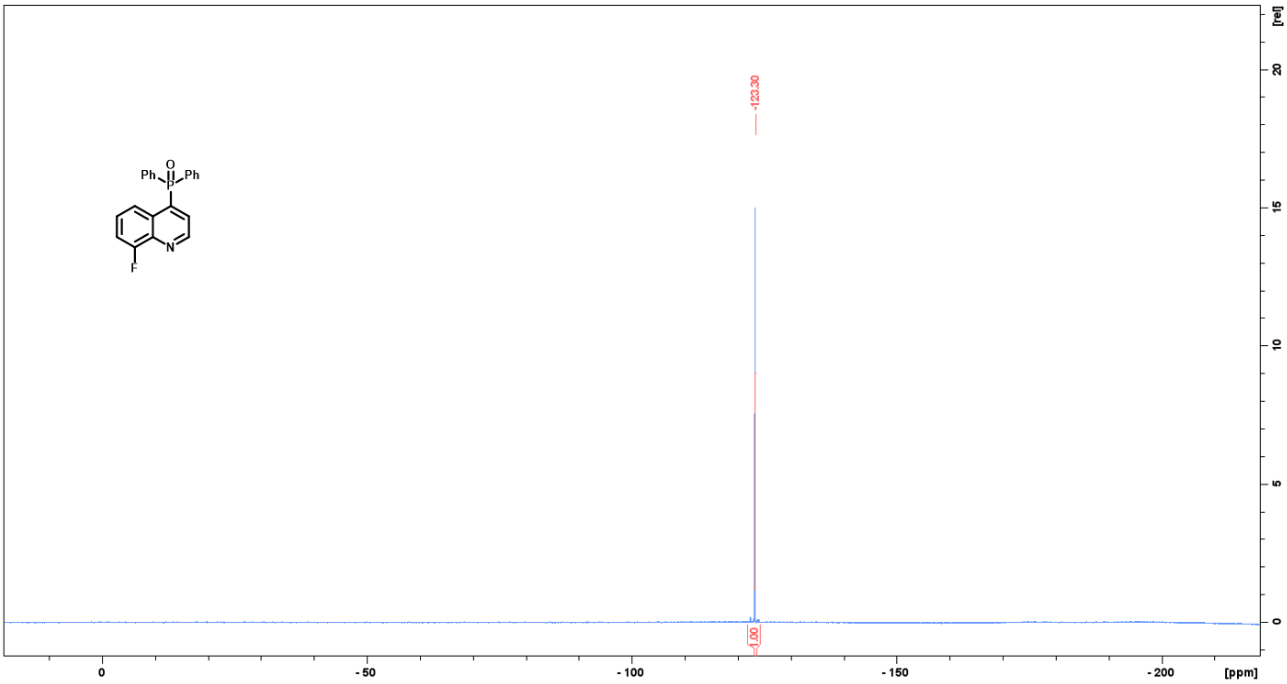

Figure S42. ^{19}F NMR (400 MHz) spectrum of Q-P in $CDCl_3$.

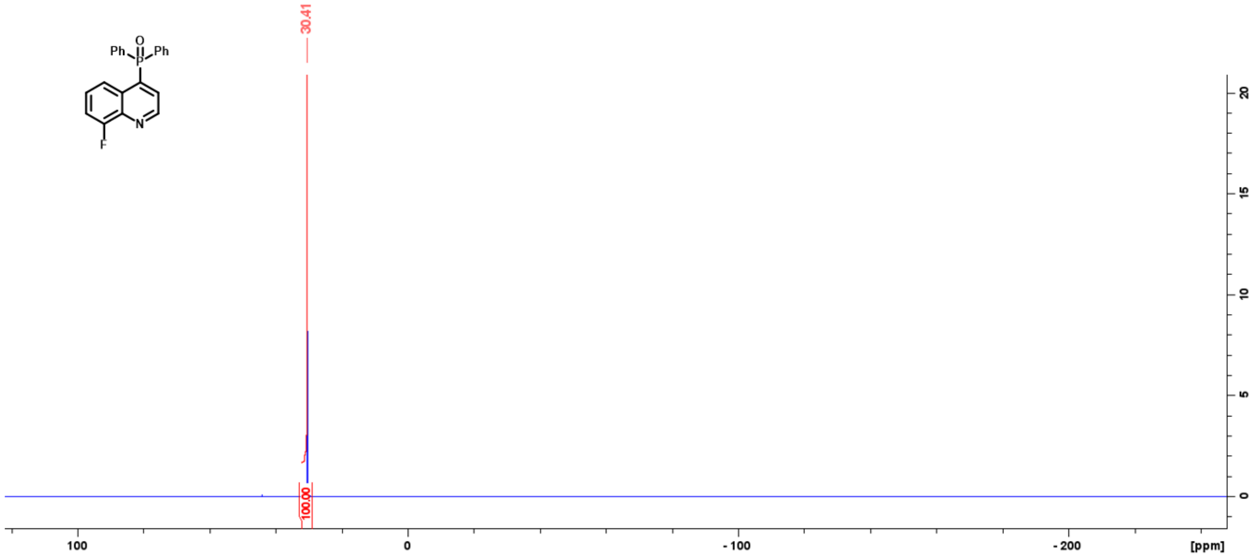

Figure S43. ^{31}P NMR (400 MHz) spectrum of Q-P in $CDCl_3$.

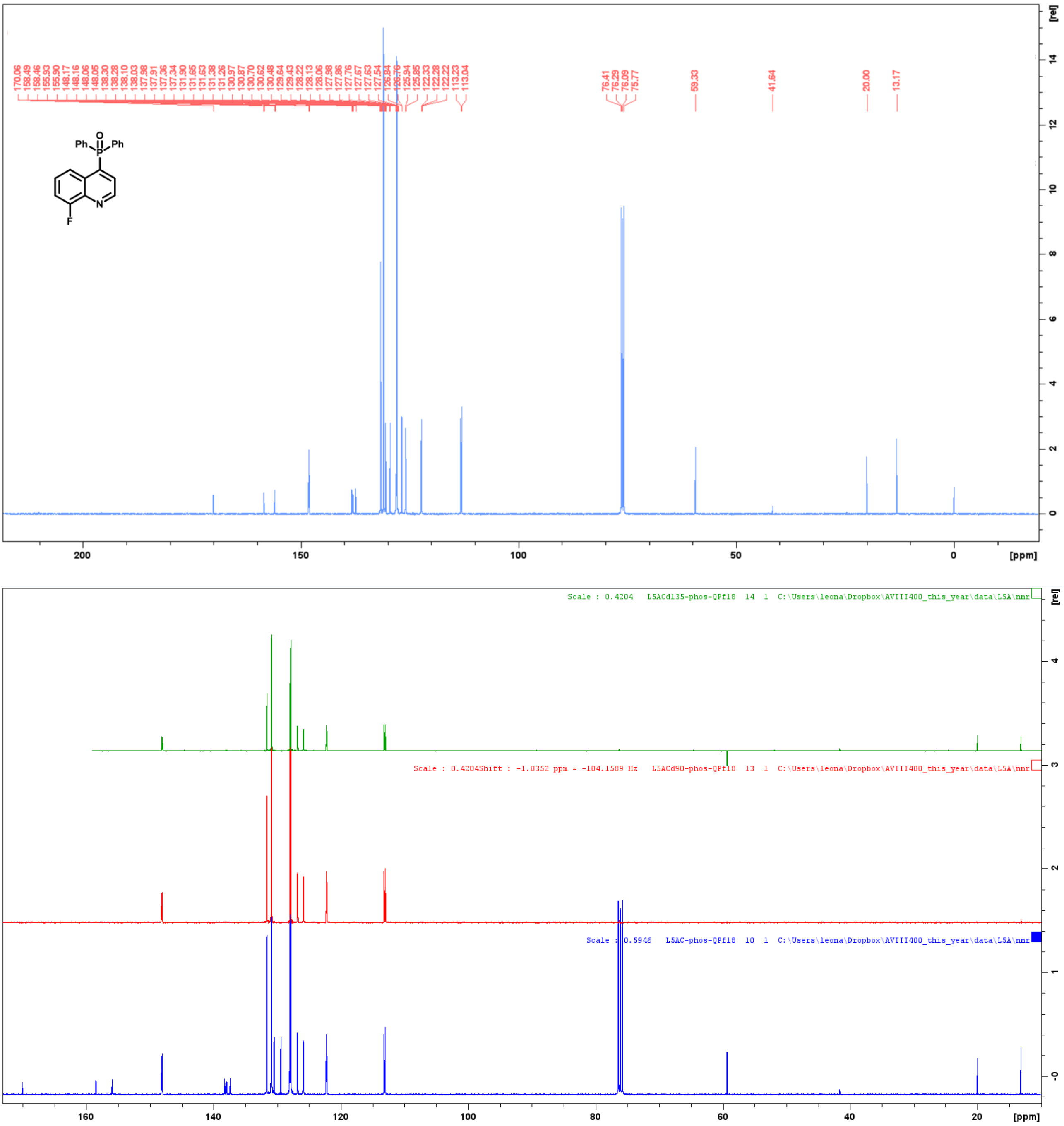

Figure S44. $^{13}C\{^1H\}$ NMR, DEPT90 and DEPT135 spectra of Q-P in $CDCl_3$.

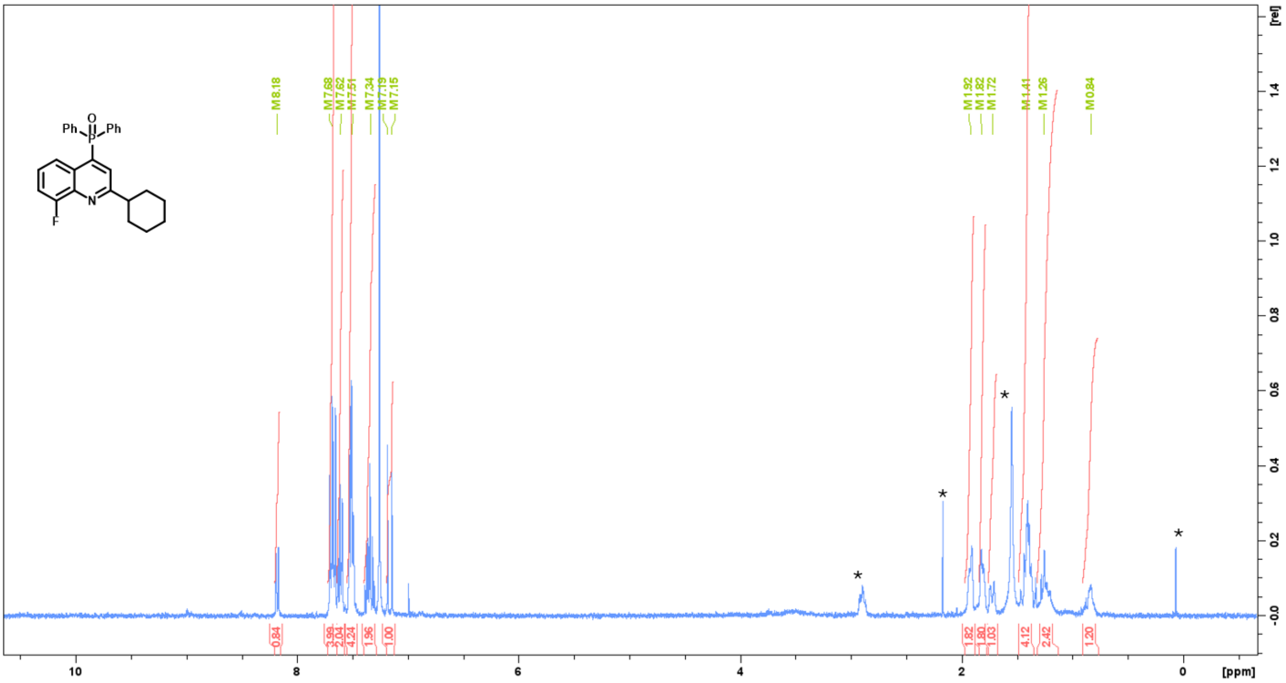

Figure S45. ^{1}H NMR (400 MHz) spectrum of Q-P-Cy in $CDCl_3$.

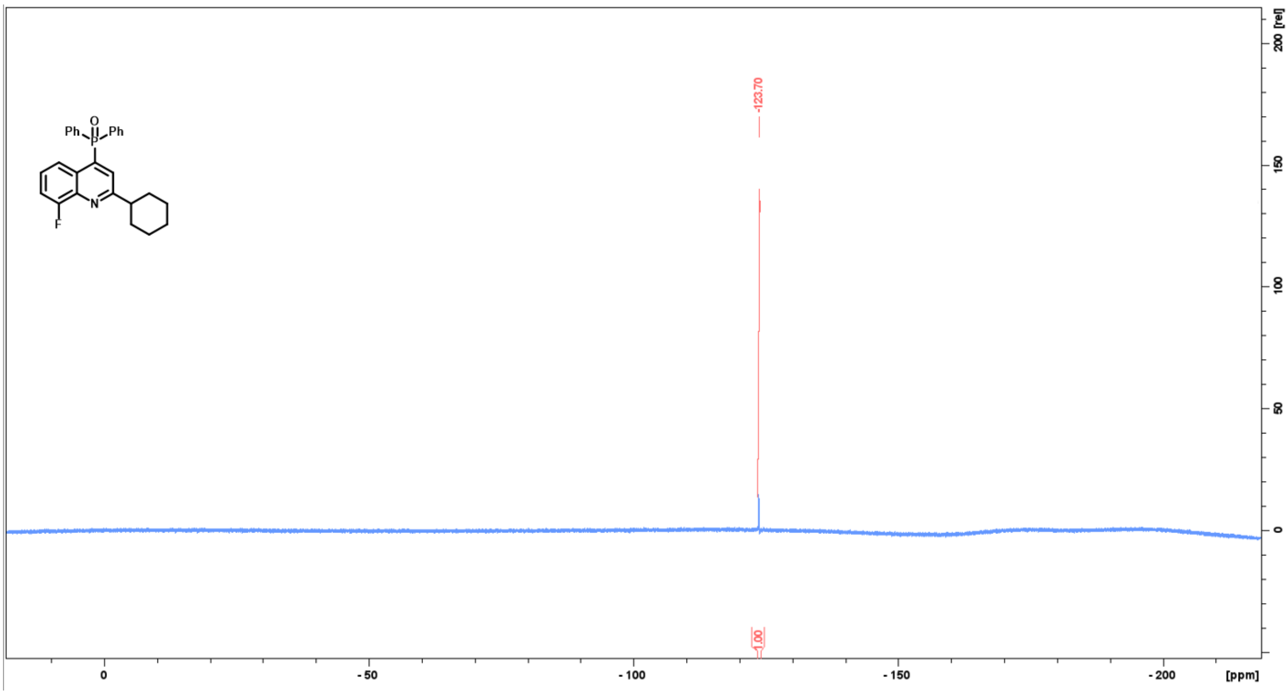

Figure S46. ^{19}F NMR (400 MHz) spectrum of Q-P-Cy in $CDCl_3$.

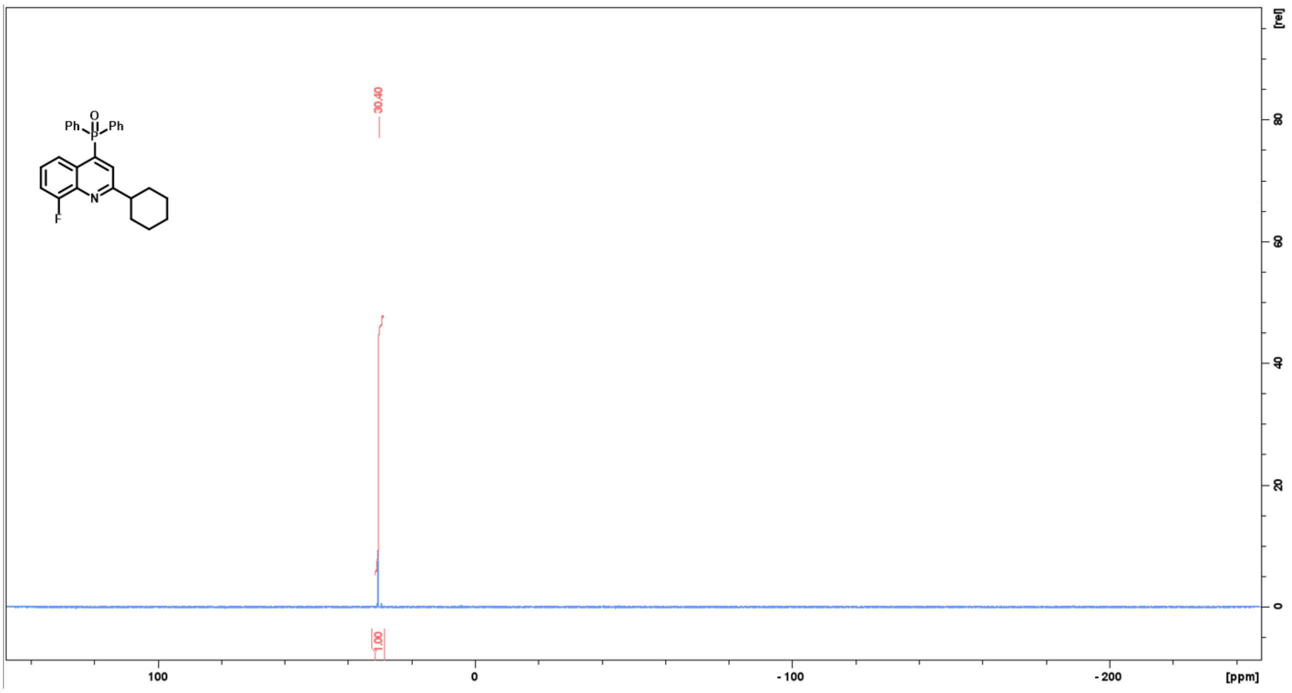

Figure S47. ^{31}P NMR (400 MHz) spectrum of Q-P-Cy in $CDCl_3$.

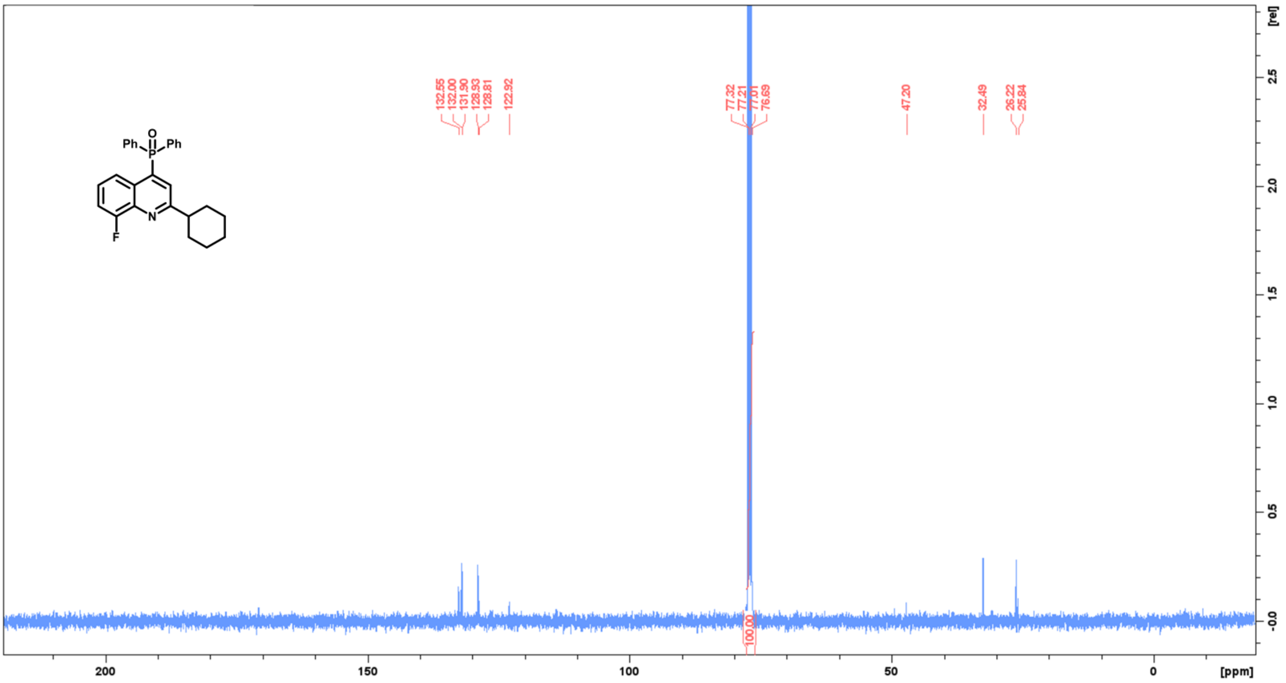

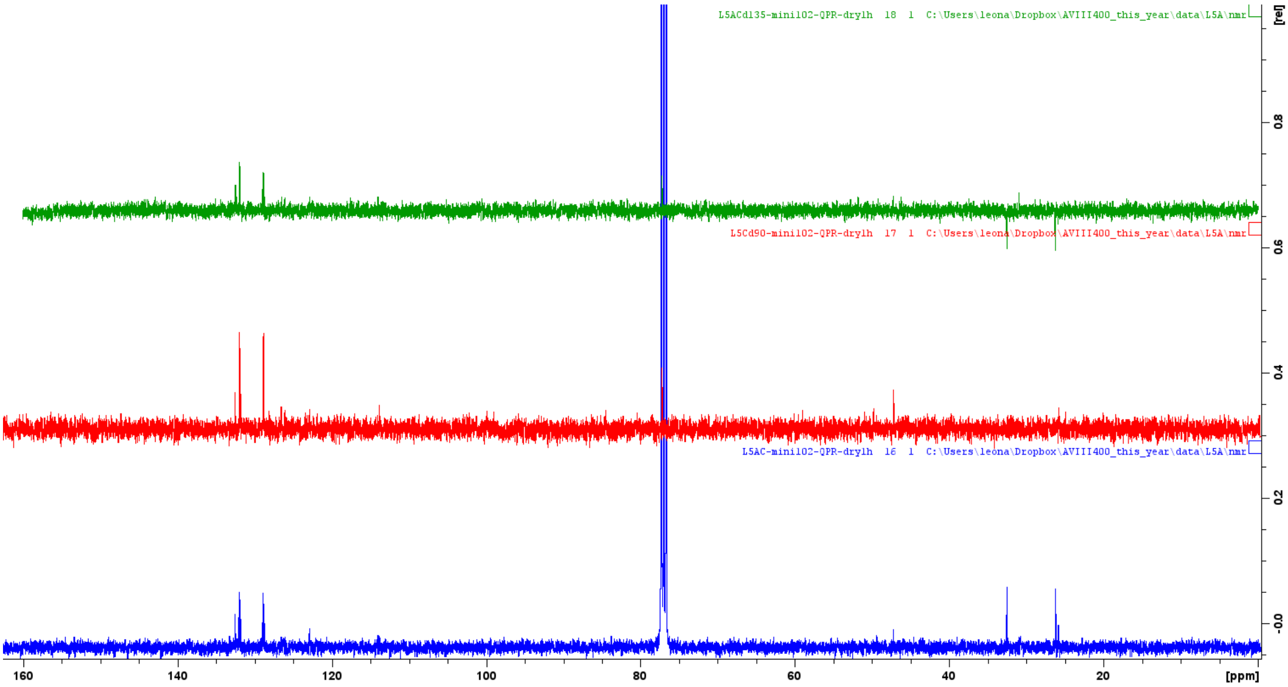

Figure S48. ${}^{13}C\{{}^{1}H\}$ NMR, DEPT90 and DEPT135 spectra of Q-P-Cy in $CDCl_3$.

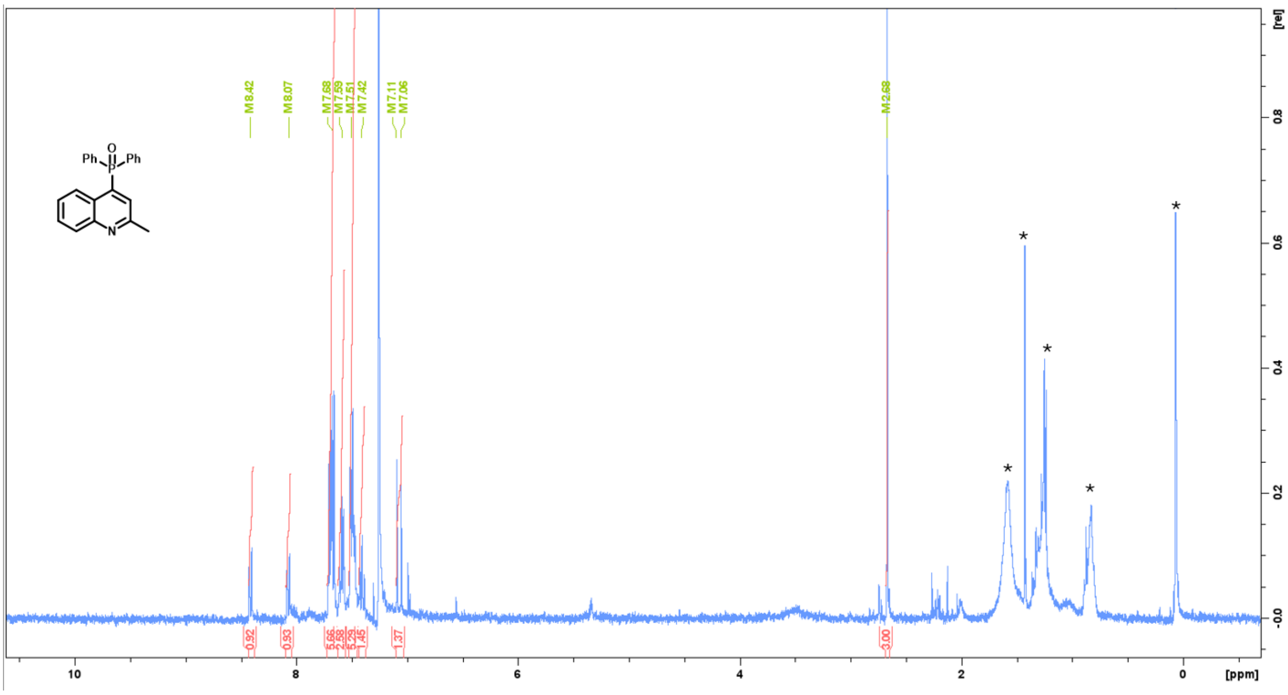

Figure S49. ${}^{1}H$ NMR (400 MHz) spectrum of Quinaldine-P in $CDCl_3$.

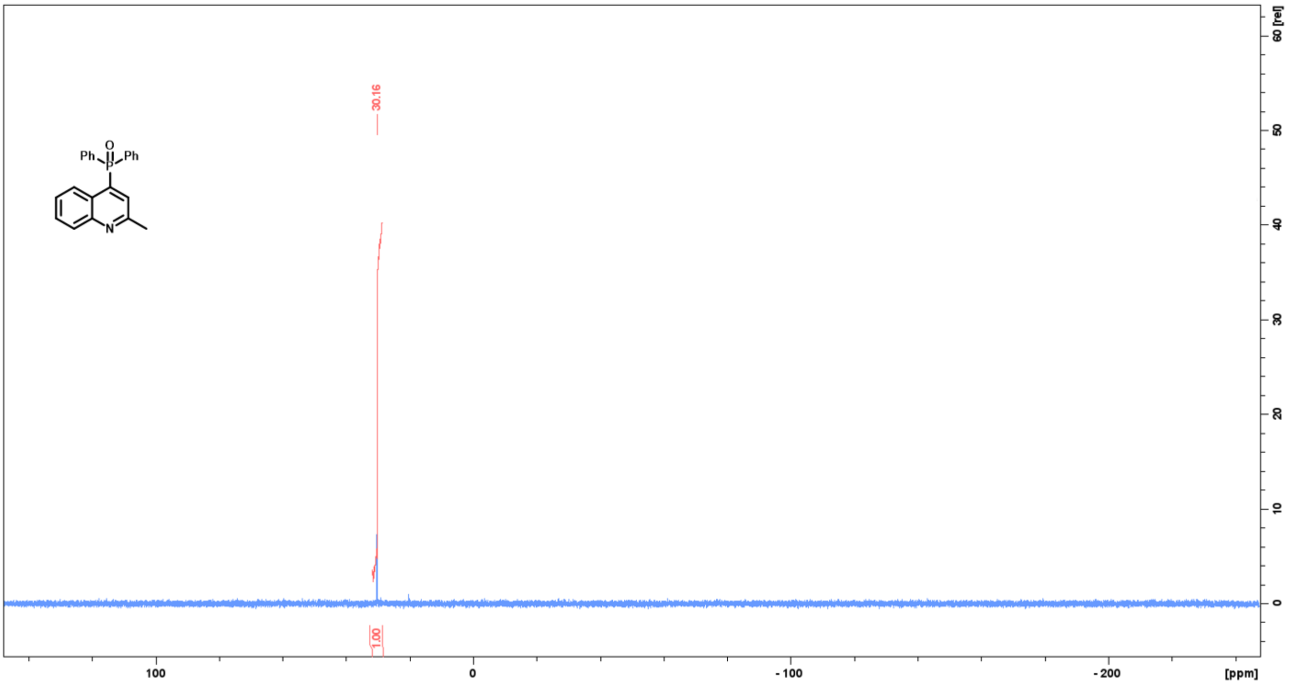

Figure S50. ^{31}P NMR (400 MHz) spectrum of Quinaldine-P in $CDCl_3$.

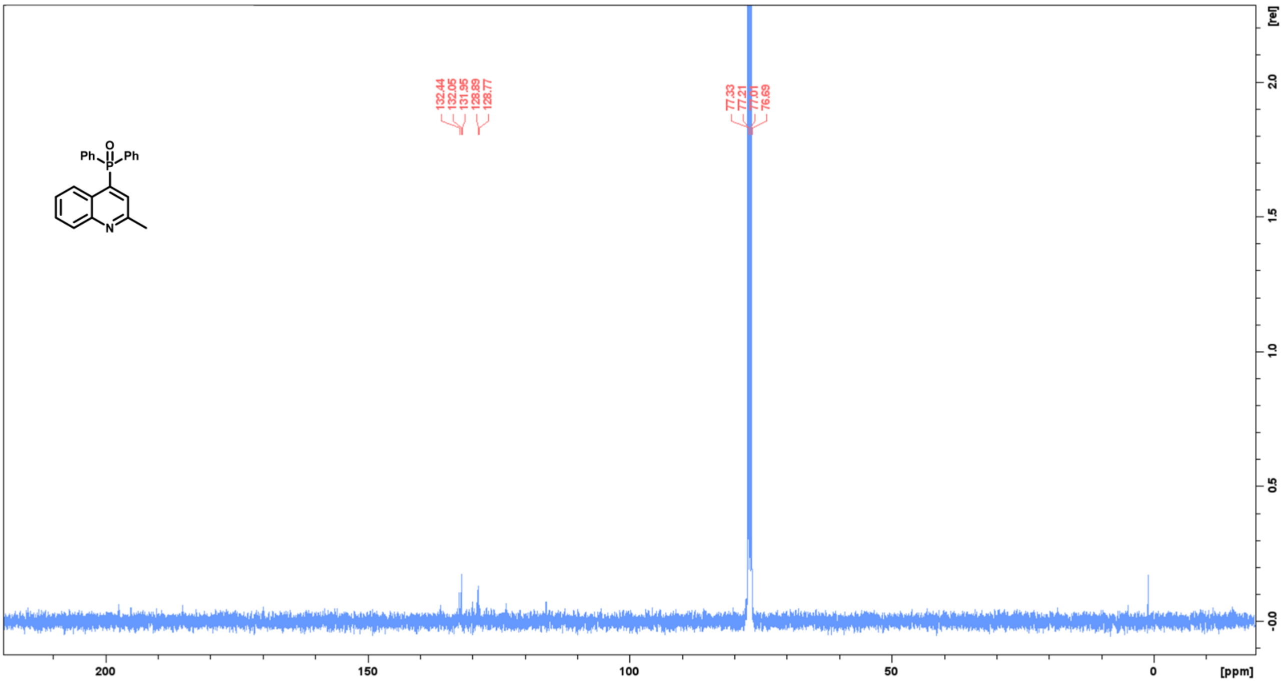

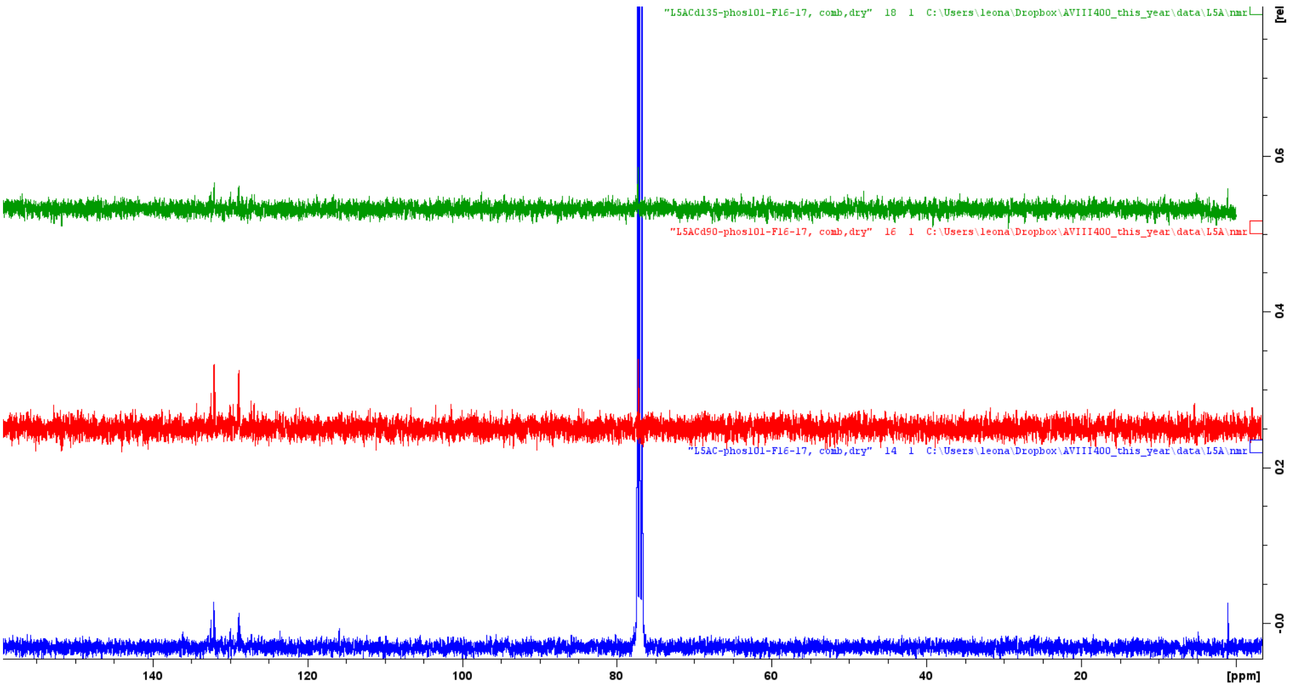

Figure S51. $^{13}C\{^1H\}$ NMR, DEPT90 and DEPT135 spectra of Quinaldine-P in $CDCl_3$.

4. Cyclic Voltammetry

The method for CV measurements was followed from literature Noémie Elgrishi *et al.*[11] The manufacturer and model of electrochemical workstation used was an IVIUMSTAT Electrochemical interface A06060 instrument. The electrodes used for the following measurements are, unless specified otherwise, glassy carbon working electrode, platinum wire counter electrode and CHI150 saturated calomel electrode (SCE, saturated KCl solution). The measurements were run in DCM (with a redox potential window of +/-2 V). The electrolyte solution was made using tetrabutylammonium hexafluorophosphate (0.1M in DCM). The analyte solution was prepared by dissolving 0.01 mmol of photophore in 10 mL of electrolyte solution. All solutions were degassed for 2 minutes with a high stream on nitrogen gas prior to the measurement. The CV plotting convention followed is IUPAC, starting the oxidation sweep to the right from 0 V (arrow on each plot) at 50 mV/s. The measurements were taken at room temperature (20 °C).

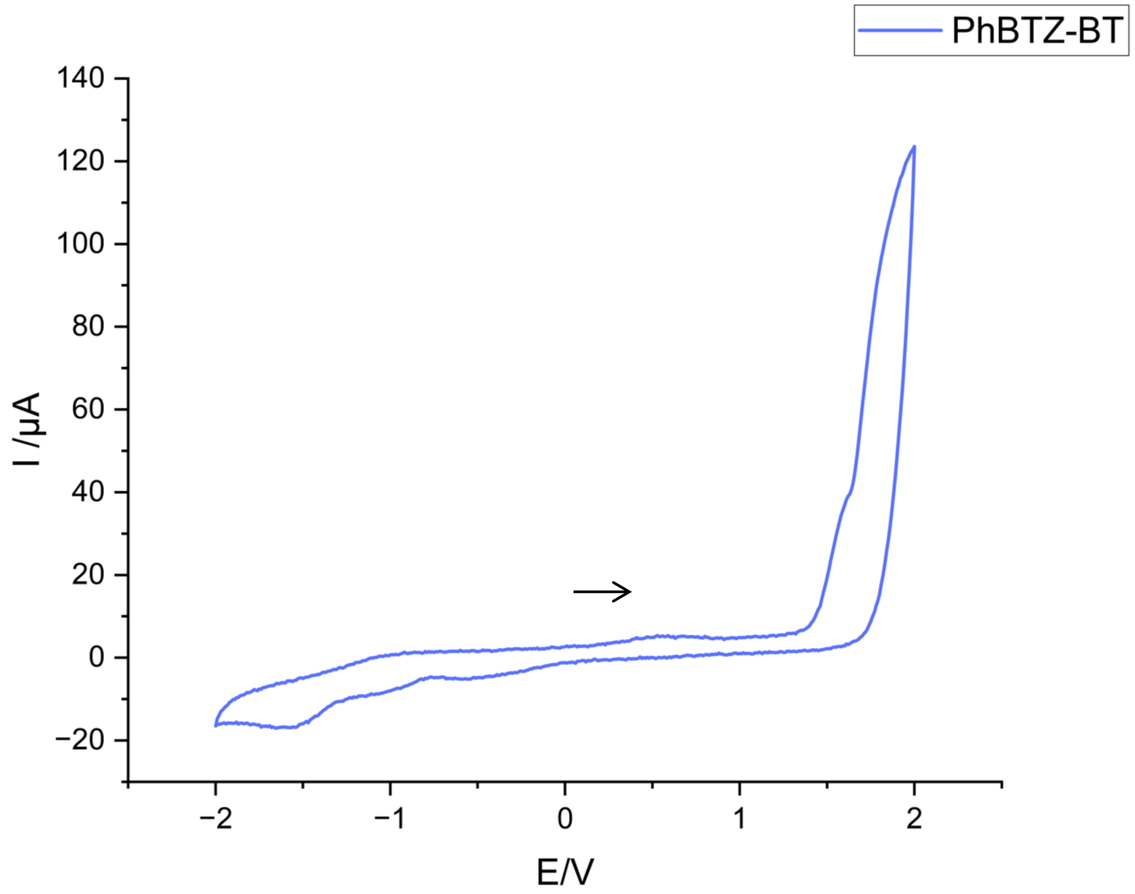

Figure S52. cyclic voltammogram of PhBTZ-BT in DCM (20 °C, IUPAC convention, starting 0 V).

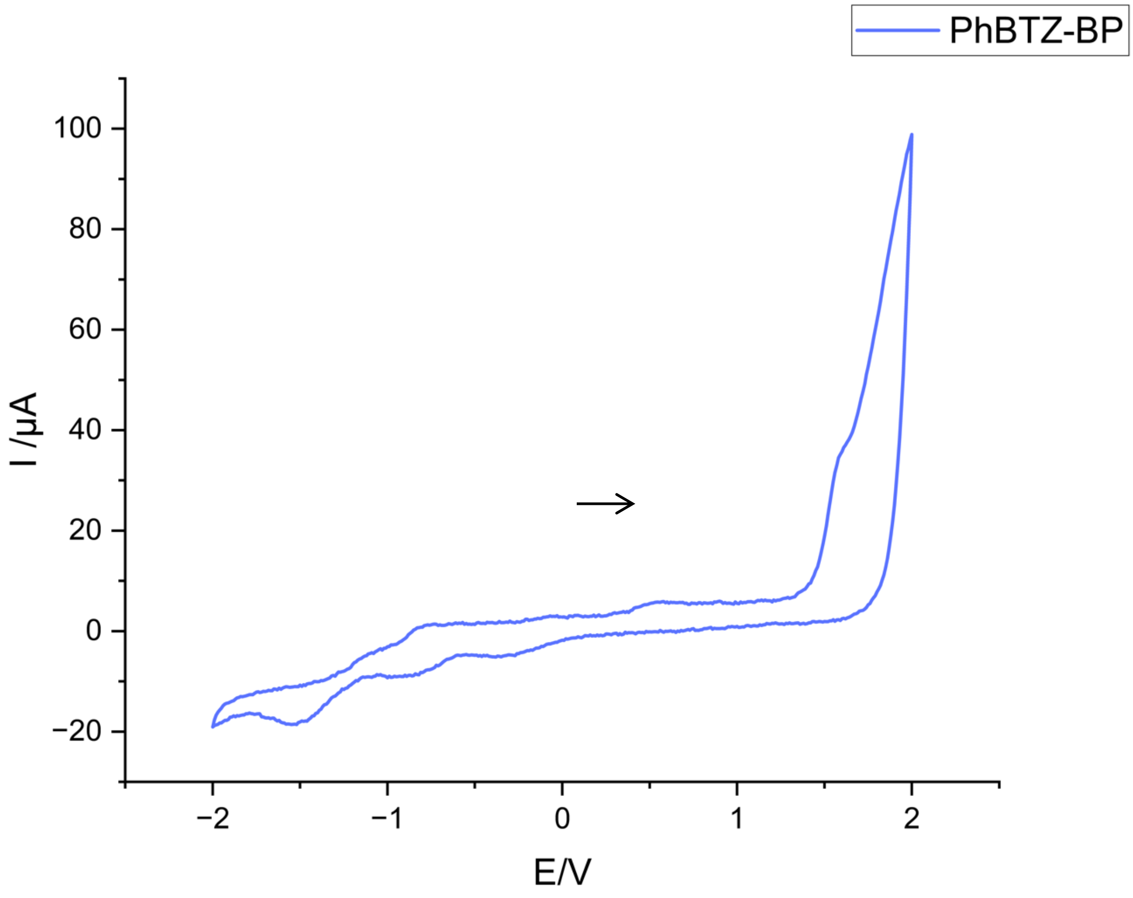

Figure S53. cyclic voltammogram of PhBTZ-BP in DCM (20 °C, IUPAC convention, starting 0 V).

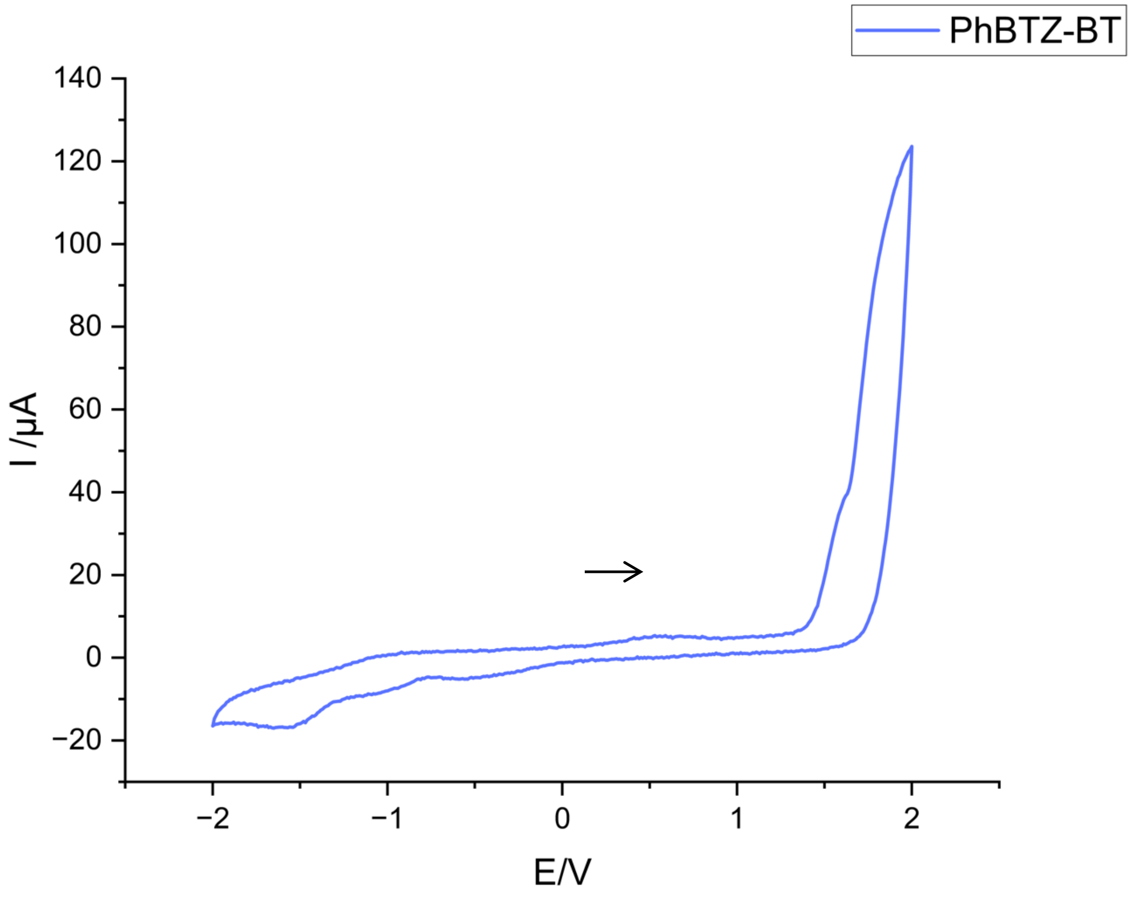

Figure S54. cyclic voltammogram of PhBTZ-BF in DCM (20 °C, IUPAC convention, starting 0 V).

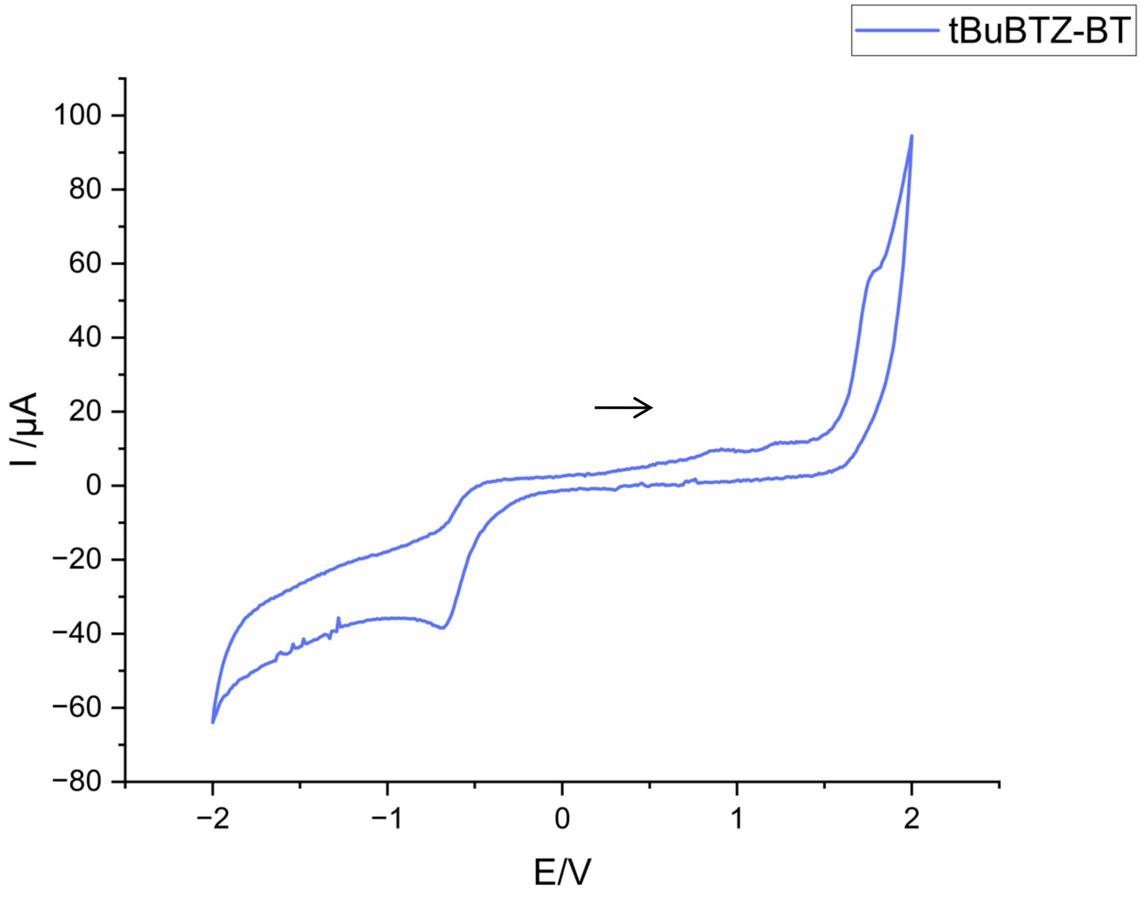

Figure S55. cyclic voltammogram of tBuBTZ-BT in DCM (20 °C, IUPAC convention, starting 0 V).

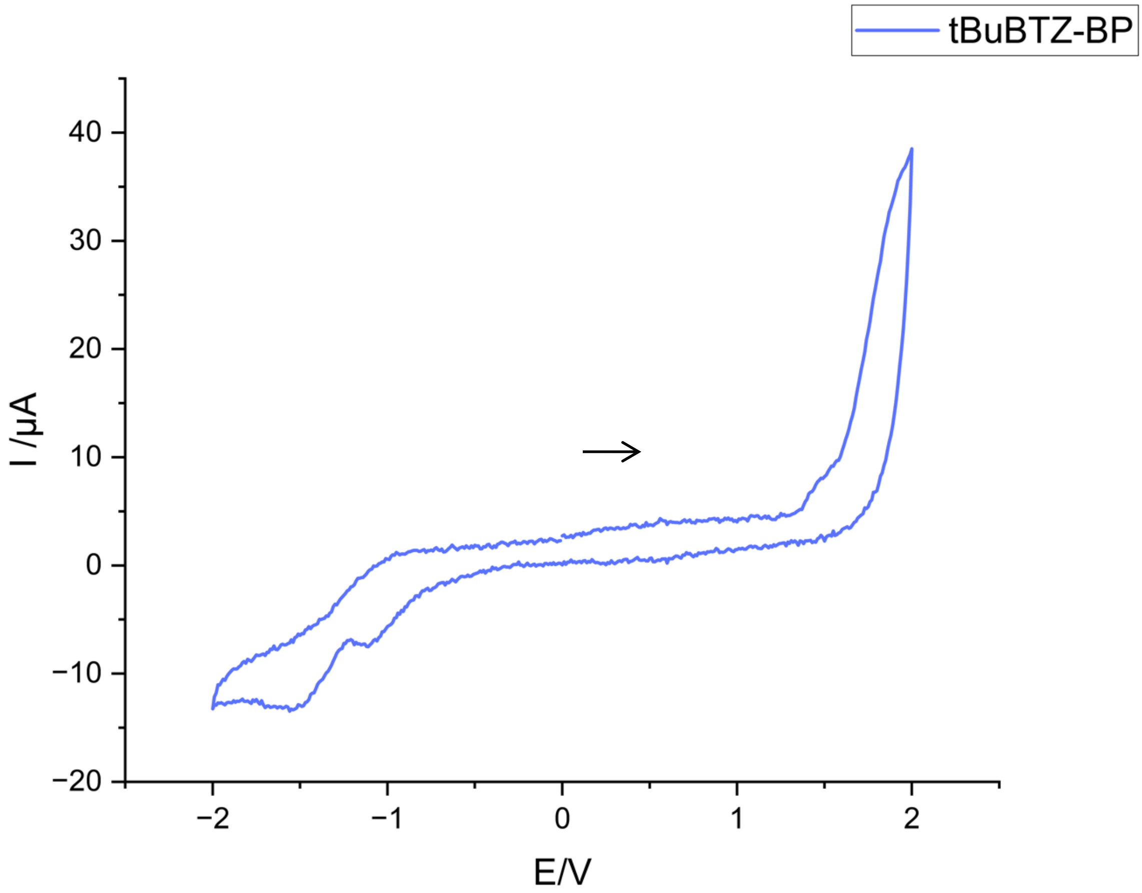

Figure S56. cyclic voltammogram of tBuBTZ-BP in DCM (20 °C, IUPAC convention, starting 0 V).

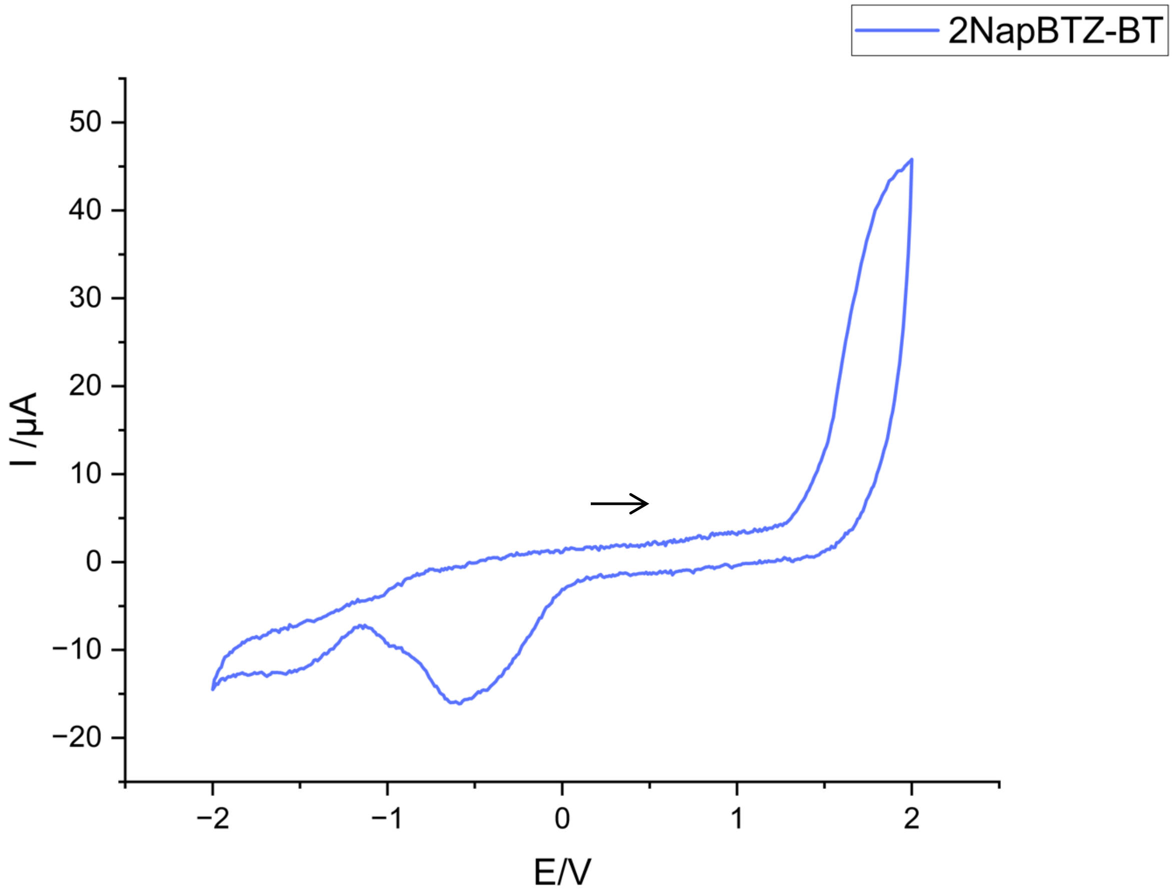

Figure S57. cyclic voltammogram of 2NapBTZ-BT in DCM (20 °C, IUPAC convention, starting 0 V).

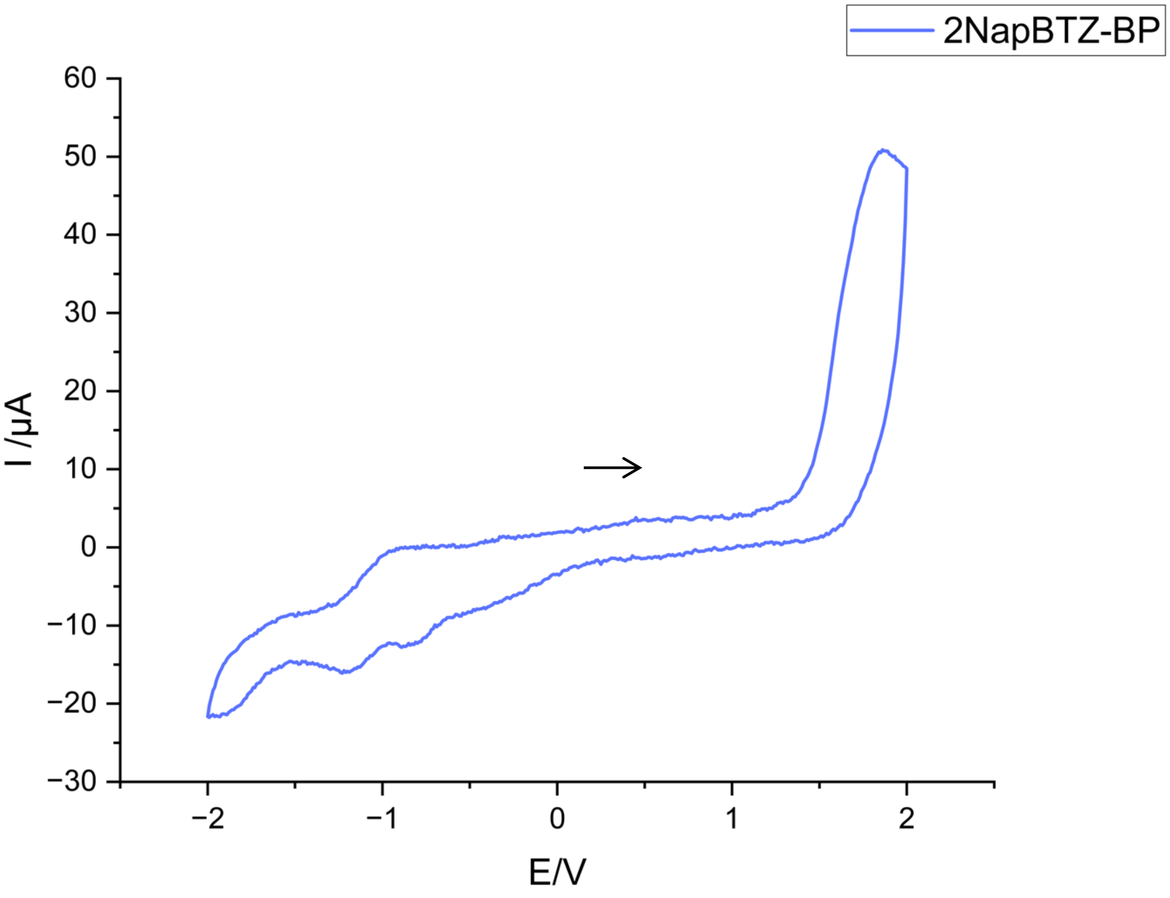

Figure S58. cyclic voltammogram of 2NapBTZ-BP in DCM (20 °C, IUPAC convention, starting 0 V).

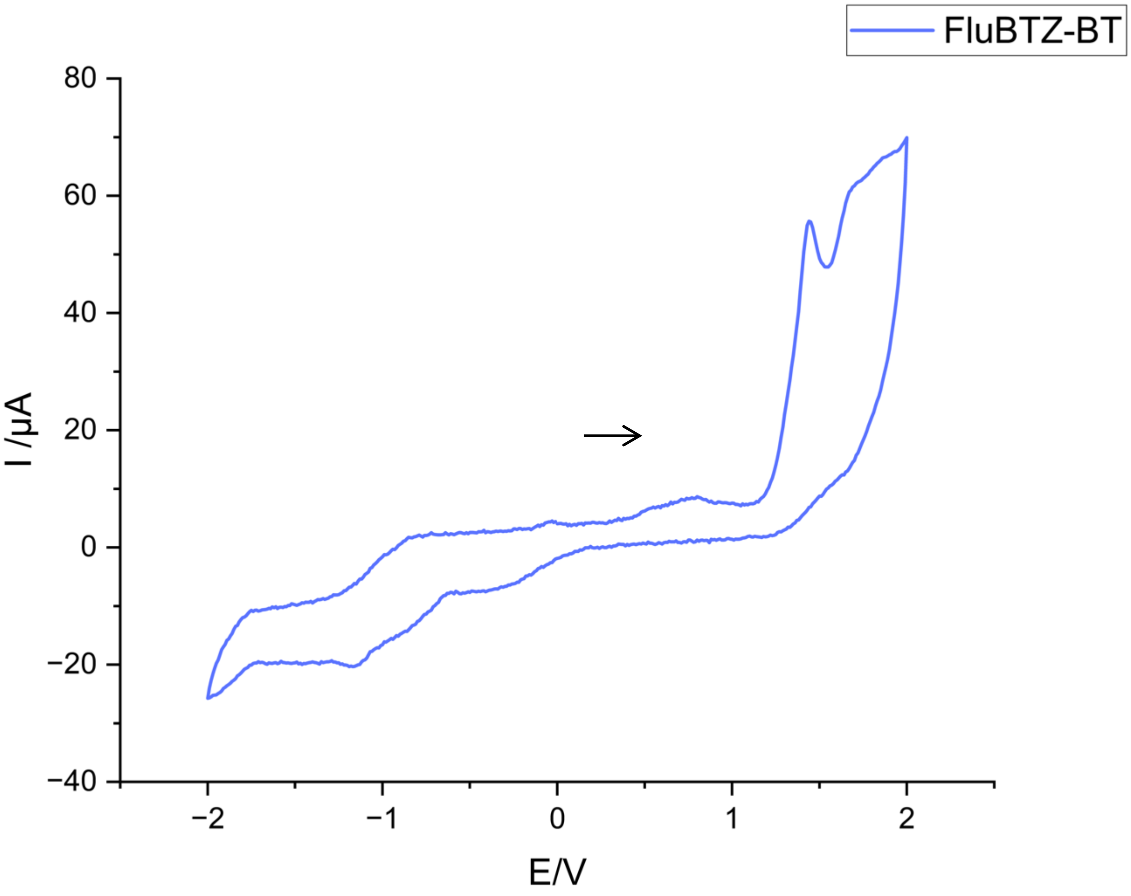

Figure S59. cyclic voltammogram of FluBTZ-BT in DCM (20 ^{o}C, IUPAC convention, starting 0 V).

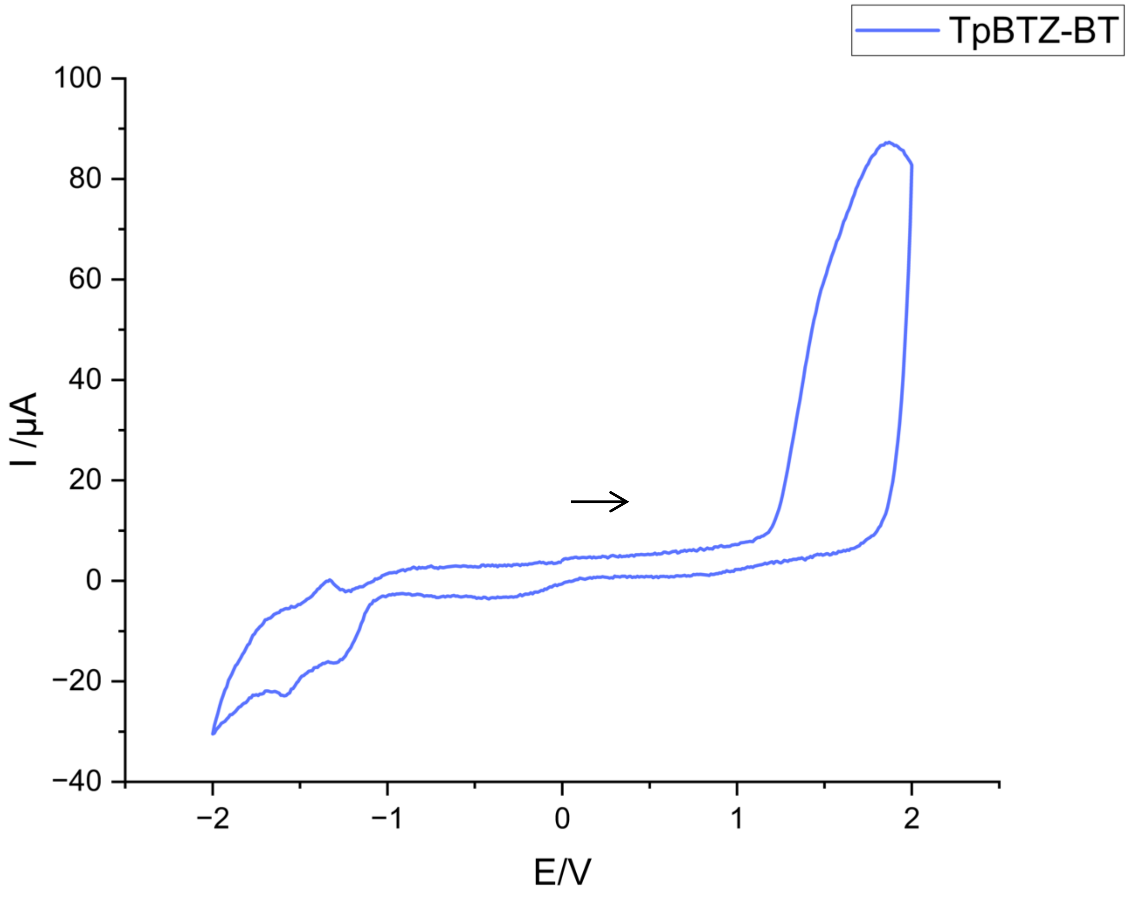

Figure S60. cyclic voltammogram of TpBTZ-BT in DCM (20 ^{o}C, IUPAC convention, starting 0 V).

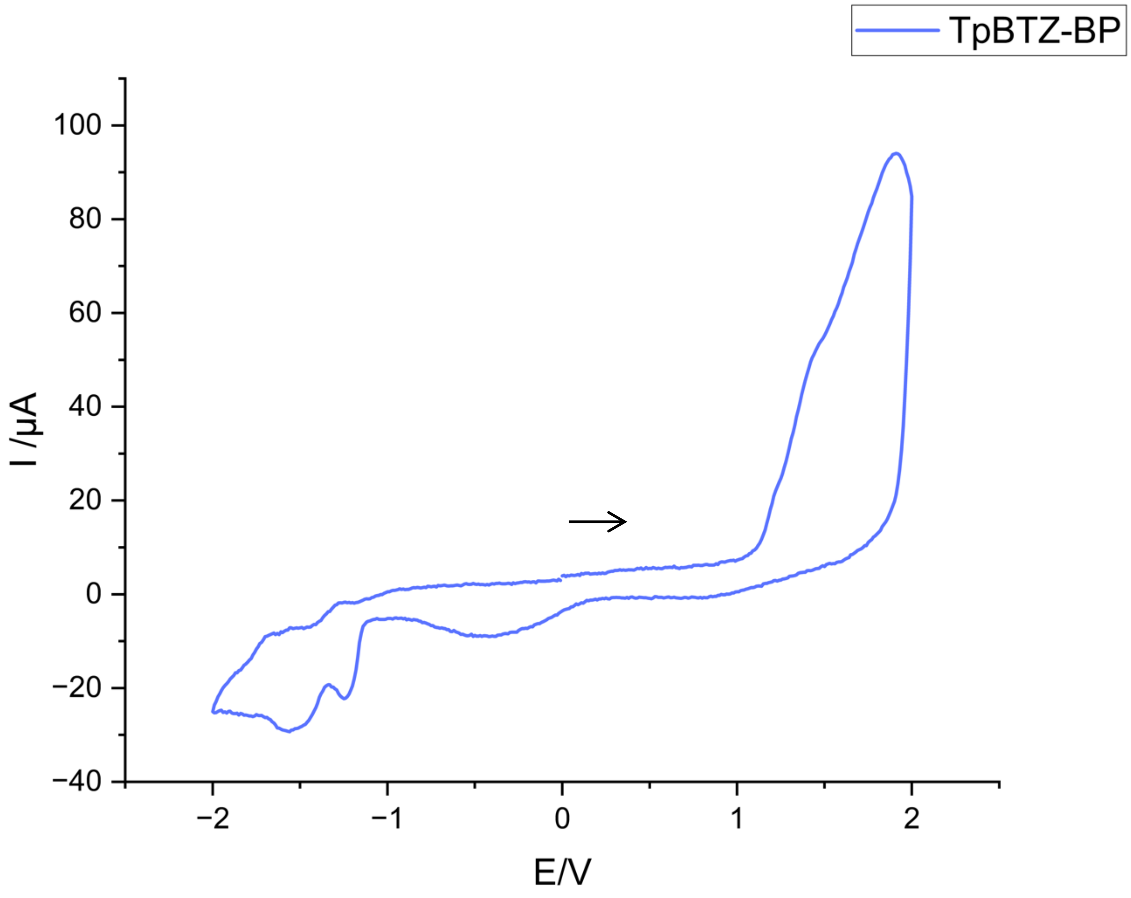

Figure S61. cyclic voltammogram of TpBTZ-BP in DCM (20 °C, IUPAC convention, starting 0 V).

5. X-ray Crystallographic data

The diffractormeter adopted to collect X-Ray crystallographic data was a D8 Venture instrument with Cu source. The Temperature of collection was 100(2) K unless specified otherwise in each cif. Submitted to the CCDC.

All crystals were grown using slow evaporation (from toluene or chloroform) or vapour diffusion (dichloromethane/ hexane).

Anisotropic displacement parameters (ADPs) of atoms are displayed at a 50% probability level

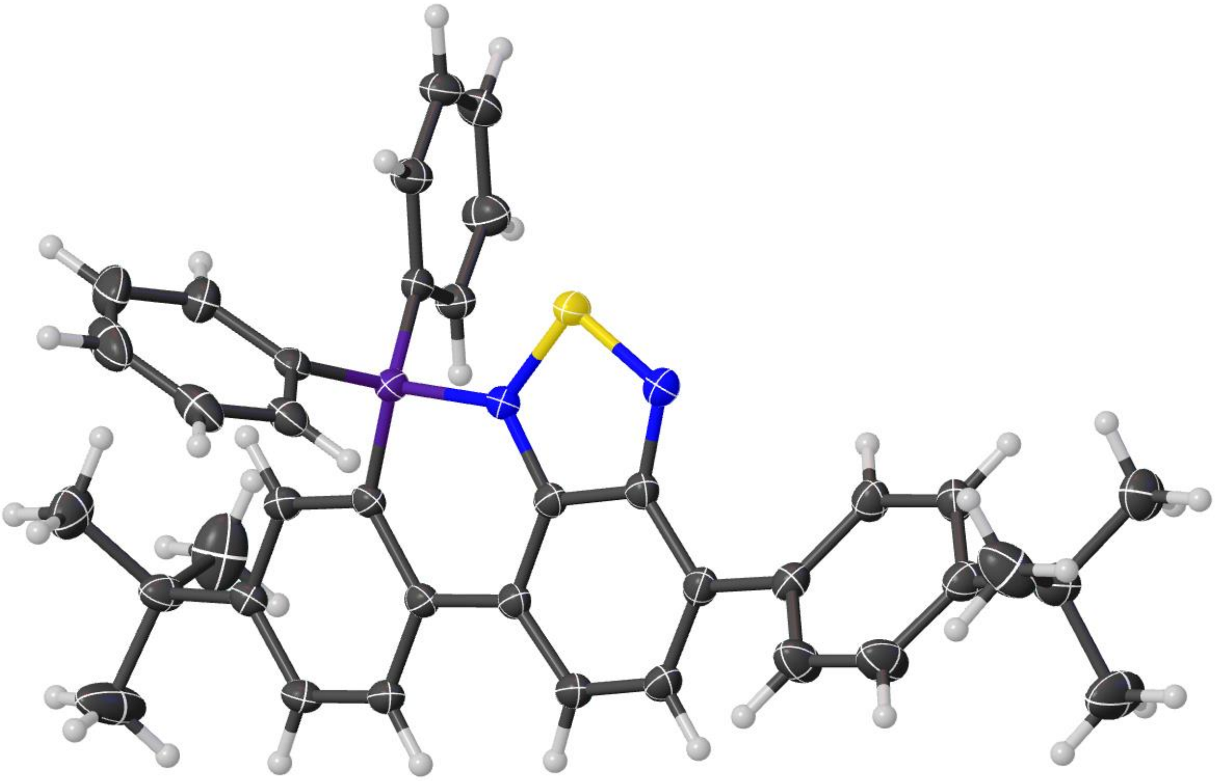

Figure S62. X-ray crystal structure of tBuBTZ-BP.

CCDC: 2447625

Table 1 Crystal data and structure refinement for tBuBTZ-BP_3.

Identification code	tBuBTZ-BP_3
Empirical formula	$C_{38}H_{37}BN_2S$
Formula weight	564.56
Temperature/K	100(2)
Crystal system	monoclinic
Space group	$P2_1/c$
a/Å	16.3154(3)
b/Å	12.1101(2)
c/Å	17.4940(3)
α/°	90
β/°	113.2930(10)
γ/°	90
Volume/Å3	3174.76(10)
Z	4
ρcalcg/cm3	1.181
μ/mm-1	1.108
F(000)	1200.0
Crystal size/mm3	0.44 × 0.08 × 0.06
Radiation	CuKα (λ = 1.54178)
2Θ range for data collection/°	9.144 to 144.248
Index ranges	-20 ≤ h ≤ 20, -13 ≤ k ≤ 14, -21 ≤ l ≤ 21
Reflections collected	63351
Independent reflections	6230 [R_{int} = 0.0713, R_{sigma} = 0.0339]
Data/restraints/parameters	6230/0/385
Goodness-of-fit on F^2	1.039
Final R indexes [I>=2σ (I)]	R_1 = 0.0464, wR_2 = 0.1218

Final R indexes [all data]	R_1 = 0.0554, wR_2 = 0.1293
Largest diff. peak/hole / e Å-3	0.45/-0.54

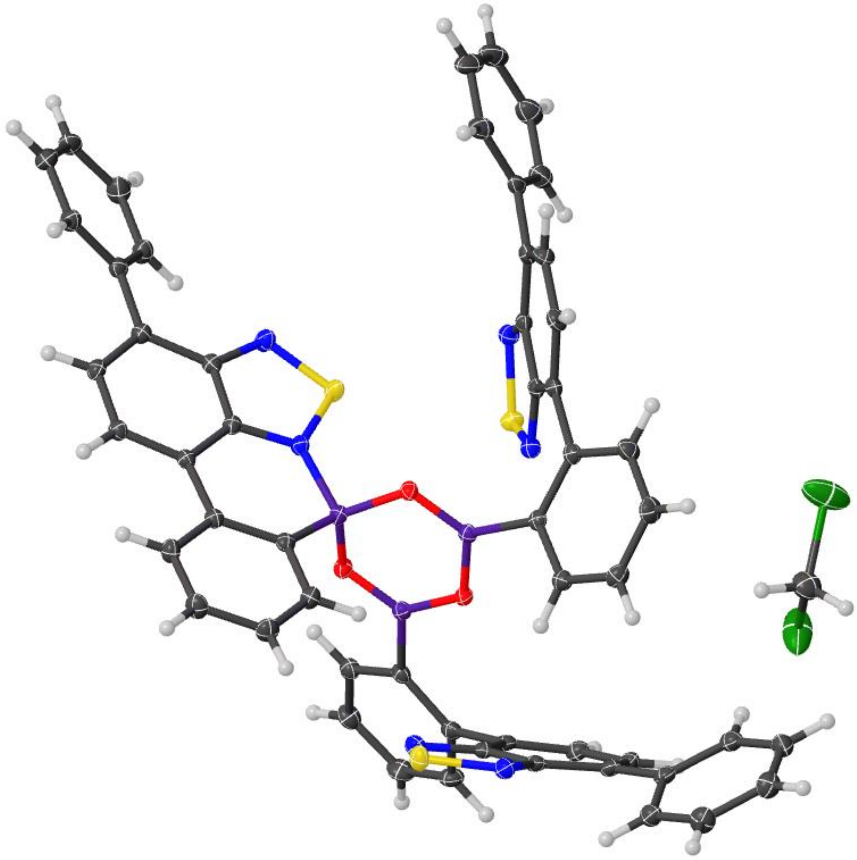

Figure S63. PhBTZ-BO3.

CCDC: 2447627

Table 1 Crystal data and structure refinement for PhBTZ-BO3_5.

Identification code	PhBTZ-BO3_5
Empirical formula	$C_{55}H_{35}B_3Cl_2N_6O_3S_3$
Formula weight	1027.40
Temperature/K	102(2)
Crystal system	triclinic
Space group	*P*-1
a/Å	10.0874(2)
b/Å	13.9928(3)
c/Å	17.8882(4)
α/°	101.1850(10)
β/°	97.3530(10)
γ/°	104.0960(10)
Volume/Å3	2361.30(9)
Z	2
ρcalcg/cm3	1.445
μ/mm-1	2.916
F(000)	1056.0
Crystal size/mm3	0.22 × 0.1 × 0.06
Radiation	CuKα (λ = 1.54178)
2Θ range for data collection/°	5.124 to 144.33
Index ranges	-12 ≤ h ≤ 12, -17 ≤ k ≤ 16, -22 ≤ l ≤ 22
Reflections collected	76081
Independent reflections	9281 [R_{int} = 0.0331, R_{sigma} = 0.0212]
Data/restraints/parameters	9281/0/649
Goodness-of-fit on F2	1.050
Final R indexes [I>=2σ (I)]	R_1 = 0.0354, wR_2 = 0.0898
Final R indexes [all data]	R_1 = 0.0369, wR_2 = 0.0909
Largest diff. peak/hole / e Å-3	0.94/-1.09

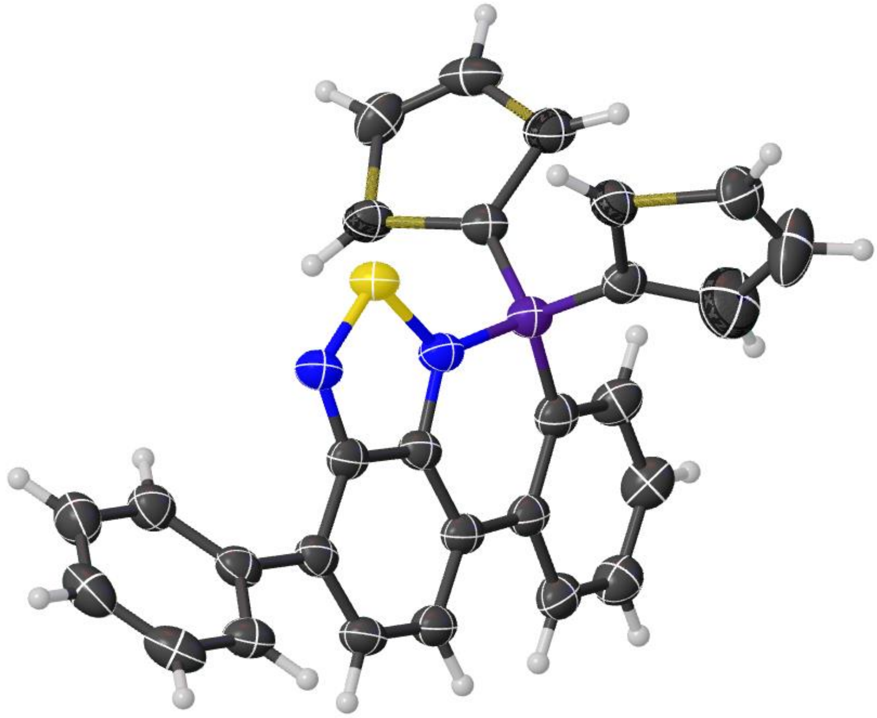

Figure S64. X-ray crystal structure of PhBTZ-BT.

CCDC: 2447623

Table 1 Crystal data and structure refinement for PhBTZ-BT_1.

Identification code	PhBTZ-BT_1
Empirical formula	$C_{52}H_{34}B_2N_4S_6$
Formula weight	928.81
Temperature/K	100.00
Crystal system	monoclinic
Space group	$P2_1/c$
a/Å	12.7594(5)
b/Å	21.6738(8)
c/Å	8.0977(3)
α/°	90
β/°	101.715(3)
γ/°	90
Volume/Å3	2192.72(14)
Z	2
ρcalcg/cm3	1.407
μ/mm-1	3.219
F(000)	960.0
Crystal size/mm3	0.34 × 0.12 × 0.02
Radiation	CuKα (λ = 1.54178)
2Θ range for data collection/°	8.158 to 140.506
Index ranges	-15 ≤ h ≤ 15, -26 ≤ k ≤ 26, -9 ≤ l ≤ 9
Reflections collected	21392
Independent reflections	4157 [R_{int} = 0.0922, R_{sigma} = 0.0758]
Data/restraints/parameters	4157/0/292
Goodness-of-fit on F2	1.057
Final R indexes [I>=2σ (I)]	R_1 = 0.0603, wR_2 = 0.1614
Final R indexes [all data]	R_1 = 0.0824, wR_2 = 0.1806
Largest diff. peak/hole / e Å-3	0.42/-0.69

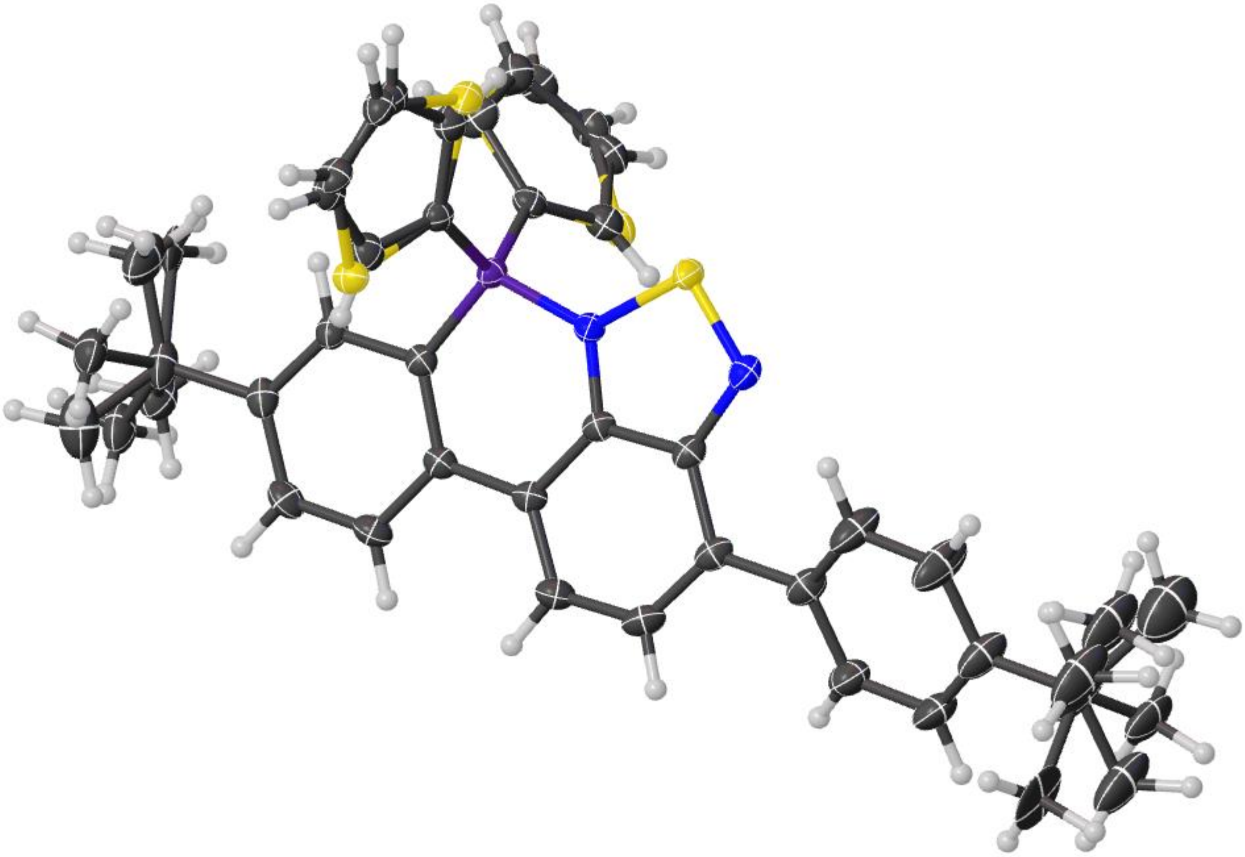

Figure S65. X-ray crystal structure of tBuBTZ-BT.

CCDC: 2447624

Table 1 Crystal data and structure refinement for tBuBTZ-BT_2.

Identification code	tBuBTZ-BT_2
Empirical formula	$C_{34}H_{33}BN_2S_3$
Formula weight	576.61
Temperature/K	100.00
Crystal system	monoclinic
Space group	$P2_1/c$
a/Å	16.2610(2)
b/Å	11.0606(2)
c/Å	17.5484(2)
α/°	90
β/°	106.4920(10)
γ/°	90
Volume/Å3	3026.34(8)
Z	4
ρcalcg/cm3	1.266
μ/mm-1	2.429
F(000)	1216.0
Crystal size/mm3	0.24 × 0.14 × 0.06
Radiation	CuKα (λ = 1.54178)
2Θ range for data collection/°	9.568 to 144.496
Index ranges	-20 ≤ h ≤ 20, -13 ≤ k ≤ 13, -21 ≤ l ≤ 21
Reflections collected	89966
Independent reflections	5973 [R_{int} = 0.0459, R_{sigma} = 0.0185]
Data/restraints/parameters	5973/240/489
Goodness-of-fit on F2	1.068
Final R indexes [I>=2σ (I)]	R_1 = 0.0401, wR_2 = 0.0978
Final R indexes [all data]	R_1 = 0.0436, wR_2 = 0.0999
Largest diff. peak/hole / e Å-3	0.63/-0.48

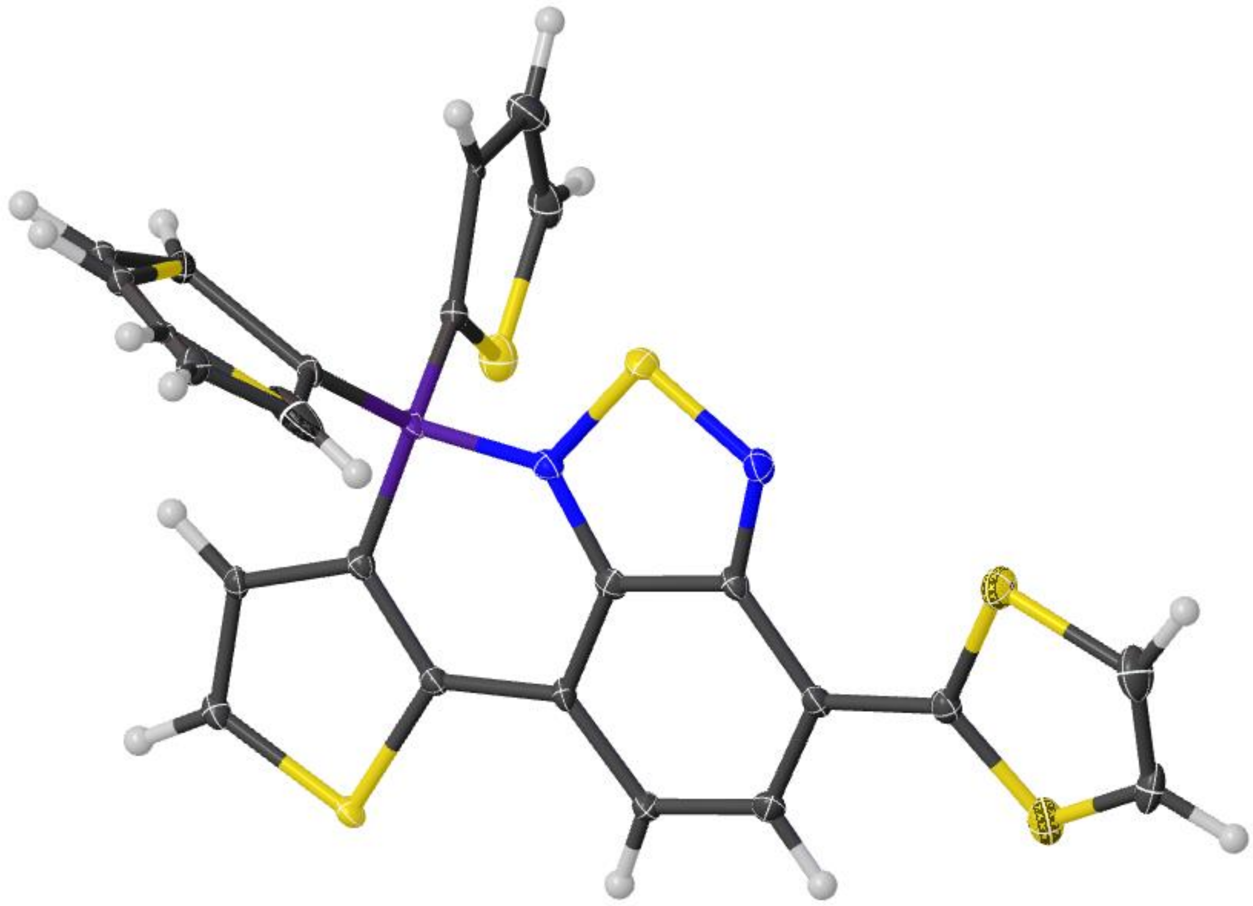

Figure S66. X-ray crystal structure of TpBTZ-BT.

CCDC: 2447626

Table 1 Crystal data and structure refinement for TpBTZ-BT_4.

Identification code	TpBTZ-BT_4
Empirical formula	$C_{26}H_{22}BN_3S_5$
Formula weight	547.57
Temperature/K	100.00
Crystal system	monoclinic
Space group	*Cc*
a/Å	11.2666(2)
b/Å	32.3702(5)
c/Å	6.56980(10)
α/°	90
β/°	92.0540(10)
γ/°	90
Volume/Å3	2394.48(7)
Z	4
ρcalcg/cm3	1.519
μ/mm-1	4.638
F(000)	1136.0
Crystal size/mm3	0.16 × 0.04 × 0.02
Radiation	CuKα (λ = 1.54178)
2Θ range for data collection/°	8.314 to 144.472
Index ranges	-13 ≤ h ≤ 13, -39 ≤ k ≤ 39, -7 ≤ l ≤ 7
Reflections collected	40652
Independent reflections	4579 [R_{int} = 0.0785, R_{sigma} = 0.0431]
Data/restraints/parameters	4579/35/291
Goodness-of-fit on F2	1.109
Final R indexes [I>=2σ (I)]	R_1 = 0.0562, wR_2 = 0.1617
Final R indexes [all data]	R_1 = 0.0660, wR_2 = 0.1754
Largest diff. peak/hole / e Å-3	0.64/-0.48
Flack parameter	0.07(2)

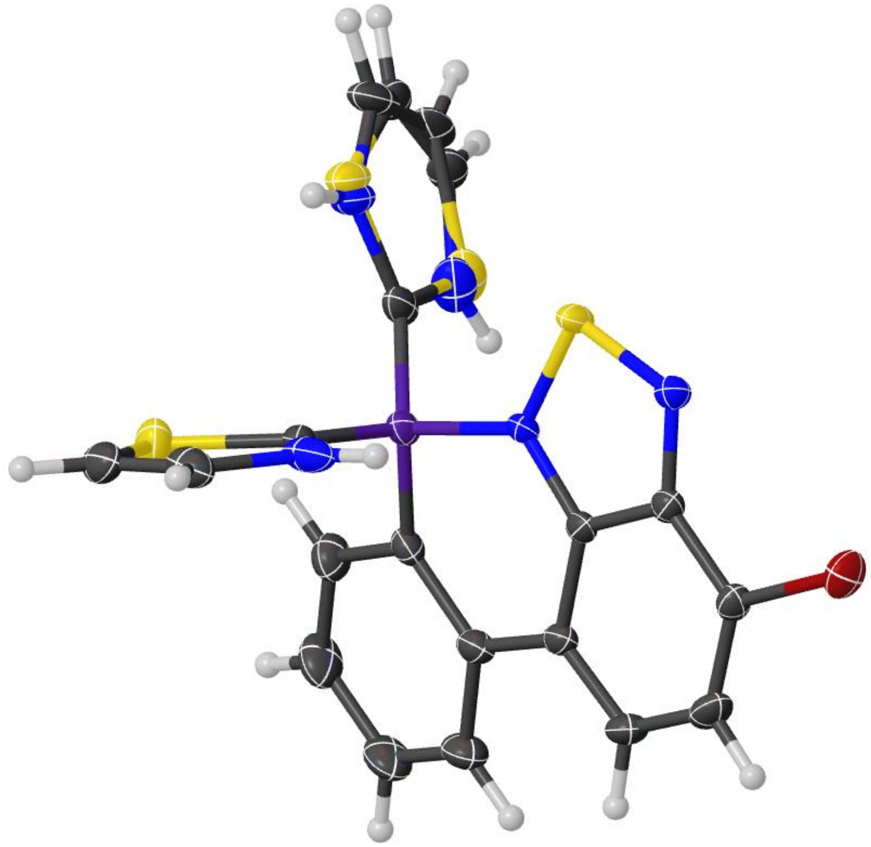

Figure S67. Crystal structure of transmetallated unsymmetrical Br-PhBTZ-BT.

CCDC: 2447628

Table 1 Crystal data and structure refinement for PhBTZ-BT-Br.

Identification code	PhBTZ-BT-Br
Empirical formula	$C_{18}H_{12}BBrN_4S_3$
Formula weight	471.22
Temperature/K	100.00
Crystal system	triclinic
Space group	*P*-1
a/Å	8.2769(2)
b/Å	9.2534(2)
c/Å	12.6605(3)
α/°	89.6680(10)
β/°	77.7400(10)
γ/°	88.0760(10)
Volume/Å3	947.01(4)
Z	2
ρcalcg/cm3	1.653
μ/mm-1	6.139
F(000)	472.0
Crystal size/mm3	0.26 × 0.2 × 0.04
Radiation	CuKα (λ = 1.54178)
2Θ range for data collection/°	7.144 to 149.154
Index ranges	-10 ≤ h ≤ 10, -11 ≤ k ≤ 11, -15 ≤ l ≤ 15
Reflections collected	23483
Independent reflections	3848 [R_{int} = 0.0423, R_{sigma} = 0.0273]
Data/restraints/parameters	3848/15/269
Goodness-of-fit on F2	1.087
Final R indexes [I>=2σ (I)]	R_1 = 0.0374, wR_2 = 0.0949
Final R indexes [all data]	R_1 = 0.0414, wR_2 = 0.0983
Largest diff. peak/hole / e Å-3	0.47/-0.62

6. Photocatalysed Phosphorylation in batch

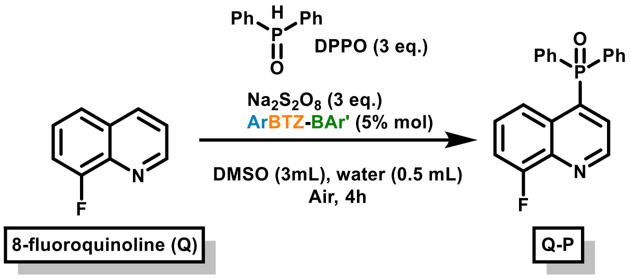

A small oven-dry glass vial containing a magnetic stirring bar was charged with 8-Fluoroquinoline (Q) (0.2 mmol, 29 mg, 1 eq.), $Na_2S_2O_8$ (0.6 mmol, 142 mg, 3 eq.), diphenylphosphine oxide (DPPO) (0.6 mmol, 121 mg, 3 eq.), and Photocatalyst (5 mol%, 0.01 mmol). Dimethylsulfoxide (DMSO) (3 mL) was then added as solvent. The vial was then placed on a stirring plate (secured using vacuum grease as glueing agent) 7 cm away from a 12 W 510 – 520 nm LED module on aluminium block, to prevent overheating during experimentation. The system was enclosed in a box covered in highly reflective mirrors, and both the light and stirring plate activated for 4 h. At the end of this time, conversion was measured by integration in a crude NMR (^{19}F NMR for Q, ^{1}H NMR for other quinoline derivatives) was performed using a 0.2 mL aliquot from the reaction mixture in 0.5 mL of $CDCl_3$. To obtain a yield instead, the reaction mixture was quenched with saturated aqueous $NaHCO_3$ (6.0 mL), washed three times with water (20 mL x3) and DCM (20 mL x3), and organic layer collected and dried over Na_2SO_4. DCM was then removed by vacuum and product was purified by flash chromatography (Ethyl acetate 100%) yielding an orange resinous solid product.

Q-P

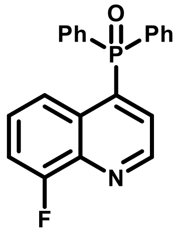

^{1}H NMR ($CDCl_3$, 400 MHz, 25.0 °C) δH 8.92 (t, 3.83 Hz, 1 H), 8.35 (d, 7.67 Hz, 1 H), 7.69-7.61 (m, 4 H), 7.61-7.54 (m, 2 H), 7.51-7.36 (m, 6 H), 7.16 (dd, 4.13-15.13 Hz, 1H). **^{13}C NMR** {1H} ($CDCl_3$, 75.5 MHz, 25.0 °C) δC 170.12 (C), 158.50 (C), 155.97 (C), 148.17 (CH), 148.04 (CH), 138.29 (C), 131.66 (CH), 131.63 (CH), 130.96 (CH), 130.87 (CH), 130.51 (C), 129.44 (C), 128.00 (CH), 127.86 (CH), 126.86 (CH), 126.77 (CH), 125.95 (CH), 125.85 (CH), 122.27 (CH), 113.23 (CH), 113.02 (CH). **^{19}F NMR** ($CDCl_3$, 400 MHz, 25.0 °C) δF -123.27 (s). **^{31}P NMR** ($CDCl_3$, 400 MHz, 25.0 °C) δP 30.41 (s). **HRMS** (ESI) m/z: [M + H]+ Calcd for C21H16FNOP 348.0948 ; Found 348.0952.

Quin-P

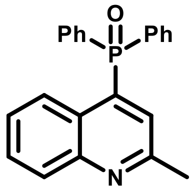

^{1}H NMR ($CDCl_3$, 400 MHz, 25.0 °C) δH 8.42 (d, 8.21 Hz, 1 H), 8.08 (d, 8.21 Hz, 1 H), 7.73-7.65 (m, 5 H), 7.62-7.56 (m, 1 H), 7.54-7.47 (m, 5 H), 7.44-7.38 (m, 1 H), 7.08 (d, 15.68 Hz, 1 H), 2.68 (s, 3 H). **^{31}P NMR** ($CDCl_3$, 400 MHz, 25.0 °C) δP 30.2 (s). **HRMS** (ESI) m/z: [M + H]+ Calcd for C22H19NOP 344.1199; Found 344.1198.

2-Q-P

^{19}**F NMR** 400MHz, s, 121 ppm;

^{31}**P NMR** 400MHz, s, 31.1 ppm;

^{1}**H NMR** 400MHz, 8.49 ppm (d, 8.41 Hz, 1H), 8.04 ppm (dd, 3.41-14.18 Hz, 1H), 7.99-7.92 ppm (m, 4 H), no observed **Q-P** dd at 7.17 ppm

Table S2. optimisation of reaction conditions for the phosphorylation of Quinoline derivatives. Optimal reaction conditions: 9-fluoroquinoline (Q) (0.2 mmol), Diphenylphosphine oxide (0.6 mmol) Oxidant (0.6 mmol), PC (5% mol), DMSO (3mL), deionised water (0.5 mL); reaction time: 4h; T: room temperature (Average 24°C); 12 W 510 – 520 nm LED. **(a)** Conversion determined by ^{19}F NMR for Q and ^{1}H NMR for other quinoline derivatives. **(b)** 420nm 12W LED light used instead of 520nm ones, due to higher absorption energy maximum of non-borylated PC.

Entry	PC	Conversion to product QP (%)[a]	Starting material left (%)[a]	Oxidant	Time (h)	Variation from standard conditions
1	-	9	91	$Na_2S_2O_8$	4	No PC
2	PhBTZ-BT	23	62	$Na_2S_2O_8$	4	none
3	PhBTZ-BP	33	67	$Na_2S_2O_8$	4	none
4	PhBTZ-BF	22	78	$Na_2S_2O_8$	4	none
5	tBuBTZ-BT	19	81	$Na_2S_2O_8$	4	none
6	tBuBTZ-BP	14	74	$Na_2S_2O_8$	4	none
7	2NapBTZ-BT	31	69	$Na_2S_2O_8$	4	none
8	2NapBTZ-BP	40	60	$Na_2S_2O_8$	4	none
9	FluBTZ-BT	22	78	$Na_2S_2O_8$	4	none
10	TpBTZ-BT	35	65	$Na_2S_2O_8$	4	none
11	TpBTZ-BP	27	73	$Na_2S_2O_8$	4	none
12	-	49	12	$Na_2S_2O_8$	24	24h, No PC
13	PhBTZ-BP	70	0	$Na_2S_2O_8$	24	24h, PhBTZ-BP
14	2NapBTZ-BP	82	0	$Na_2S_2O_8$	24	24h, 2NapBTZ-BP
15[b]	2NapBTZ[b]	15	85	$Na_2S_2O_8$	4	Control 2NapBTZ
16[b]	TpBTZ[b]	17	83	$Na_2S_2O_8$	4	Control TpBTZ
17	2NapBTZ-BP	19	81	$(NH_4)_2S_2O_8$	4	$(NH_4)_2S_2O_8$ oxidant
18	2NapBTZ-BP	14	86	$K_2S_2O_8$	4	$K_2S_2O_8$ oxidant
19	-	5	95	$Na_2S_2O_8$	4	No PC, Quinaldine instead of Q
20	PhBTZ-BP	13	87	$Na_2S_2O_8$	4	Quinaldine instead of Q
21	-	18	82	$Na_2S_2O_8$	24	No PC, Quinaldine instead of Q, 24h
22	PhBTZ-BP	50	50	$Na_2S_2O_8$	24	Quinaldine instead of Q, 24h
23	PhBTZ-BP	0	100	$Na_2S_2O_8$	24	Lepidine
24	PhBTZ-BP	0	100	$Na_2S_2O_8$	24	$CHCl_3$ instead of DMSO
25	PhBTZ-BP	8	92	$Na_2S_2O_8$	4	In darkness
26	2NapBTZ-BP	0	100	$Na_2S_2O_8$	4	With TEMPO (2 eq.)
27	PhBTZ-BP	35	65	$Na_2S_2O_8$	4	Internal standard test (fluorobenzene)
28	PhBTZ-BP	30	70	$Na_2S_2O_8$	4	Recovered photocatalyst
29	PhBTZ-BP	81	trace	$Na_2S_2O_8$	24	1% loading, 18 W LED, N_2 atmosphere

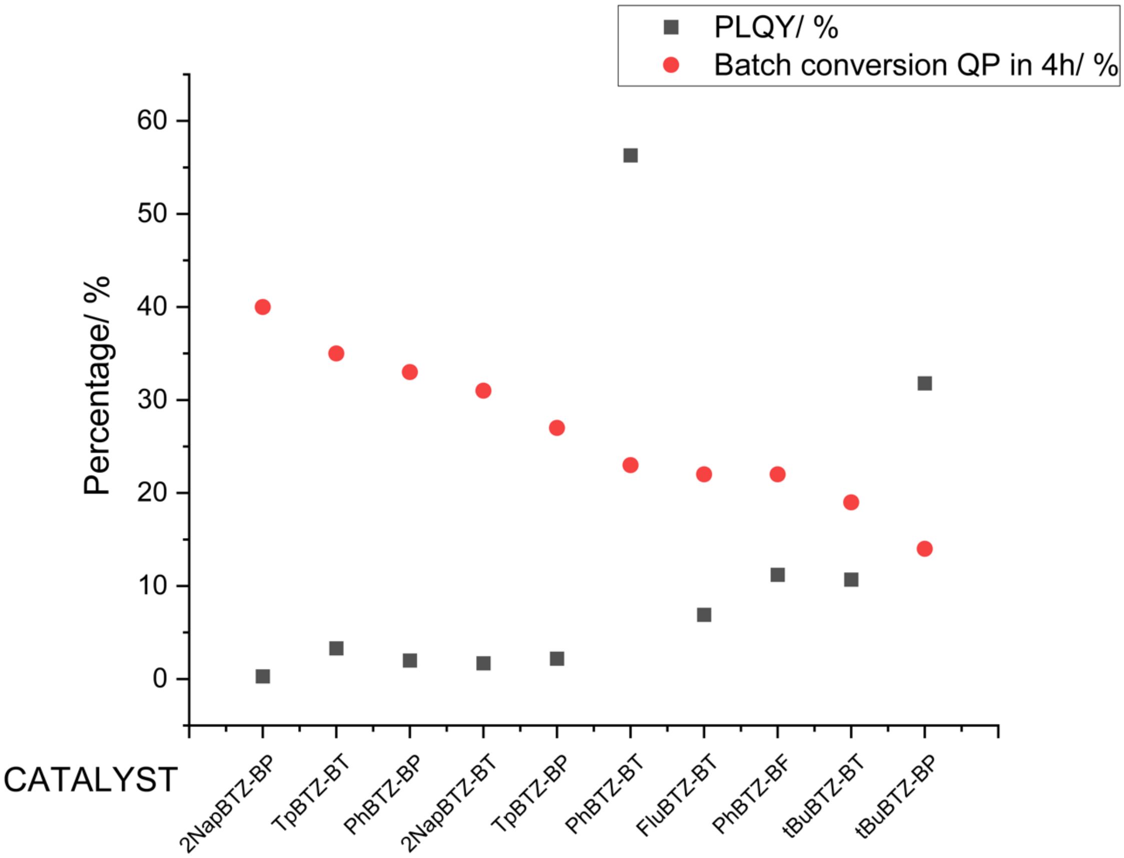

Figure S68. Batch conversion to QP in 4h and relative PLQY (%) for each tested PC in the library.

7. Computational calculations

Table S3. Computed absorption and emission wavelengths in comparison to experimental values, and ground state redox potentials computed using B3LYP/cc-pVDZ in DMSO [V]. (a) All ground state redox potentials values are computed with respect to the standard hydrogen electron and were computed *via* a Born-Haber type cycle, (b) resulting potential against standard calomel electrode (SCE), saturated KCl solution.

PC	Calculated S_1 Absorption [nm]	Experimental Absorption [nm]	Calculated S_1 Emission [nm]	Experimental Emission [nm]	Adiabatic S_1 excitation [nm]	E_{ox} Calculated[a] (NHE) [V]	E_{ox} Calculated[b] (SCE) [V]	E_{ox} Experimental[b] (SCE) [V]	E_{red} Calculated[a] (NHE) [V]	E_{red} Calculated[b] (SCE) [V]	E_{red} Experimental[b] (SCE) [V]
**Ph-BTZ-BT**	485	502	648	606	554	1.24	0.99	1.52	-0.76	-1.007	-1.46
**Ph-BTZ-BP**	488	498	645	600	554	1.21	0.96	1.51	-0.83	-1.077	-1.51
**Ph-BTZ-BF**	479	497	636	611	546	1.18	0.93	1.68	-0.81	-1.057	-1.45
**tBu-BTZ-BT**	508	524	683	630	584	1.12	0.87	1.68	-0.79	-1.037	-1.51
**tBu-BTZ-BP**	507	521	678	628	580	1.01	0.76	1.45	-0.87	-1.117	-1.58
**2Nap-BTZ-BT**	502	540	665	645	573	0.96	0.52	-	-0.66	-0.907	-0.68
**2Nap-BTZ-BP**	497	543	657	643	566	0.99	0.74	-	-0.79	-1.037	-0.95
**Flu-BTZ-BT**	513	585	683	675	597	1.00	0.75	1.33	-0.80	-1.047	-1.35
**Tp-BTZ-BT**	582	578	795	666	674	0.89	0.64	1.35	-0.70	-0.947	-0.55
**Tp-BTZ-BP**	588	586	798	700	677	0.78	0.53	1.42	-0.74	-0.987	-0.73

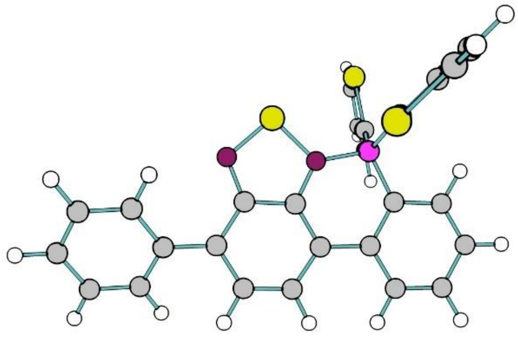

Figure S69. S1 (left) and T1 (right) structures of PhBTZ-BT.

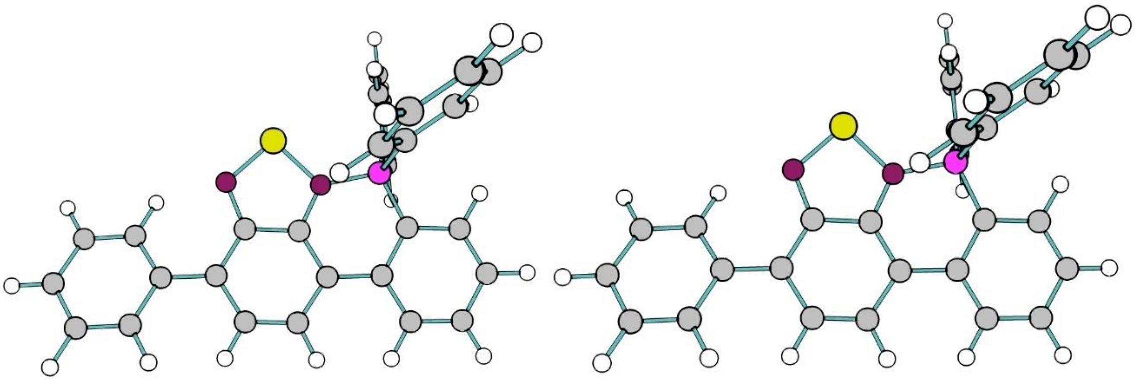

Figure S70. S1 (left) and T1 (right) structures of PhBTZ-BP.

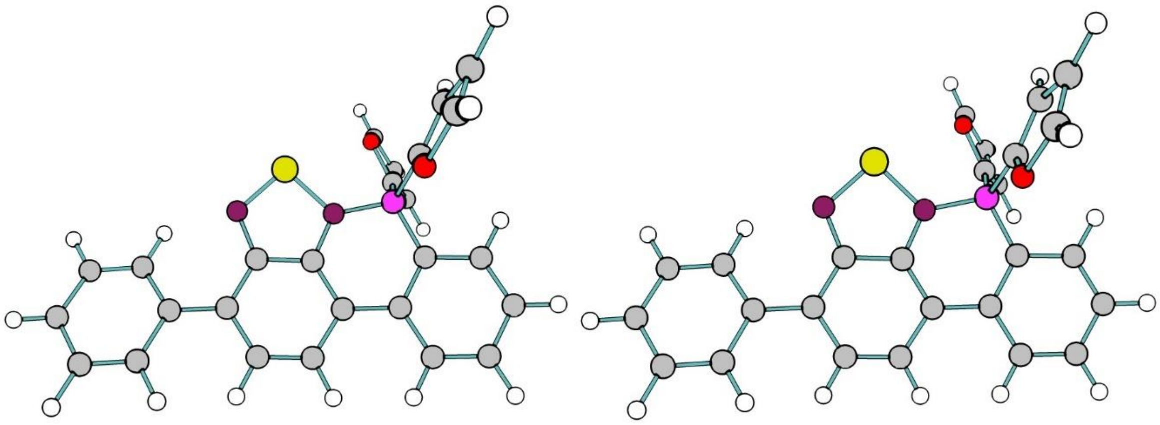

Figure S71. S1 (left) and T1 (right) structures of PhBTZ-BF.

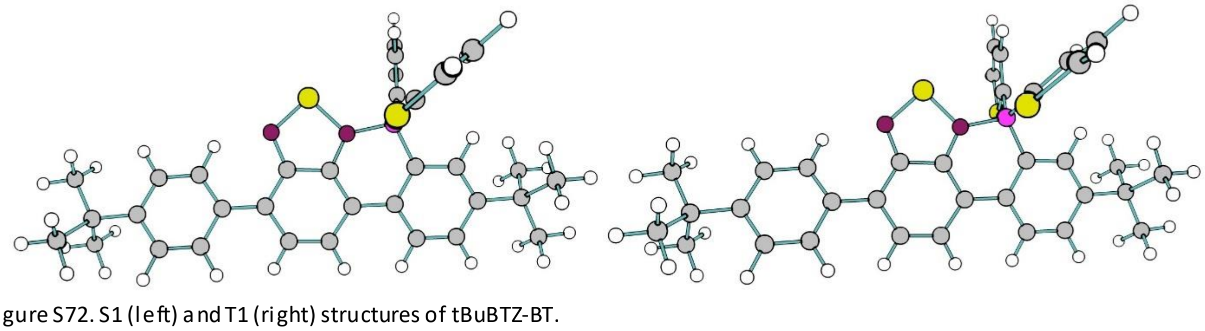

Figure S72. S1 (left) and T1 (right) structures of tBuBTZ-BT.

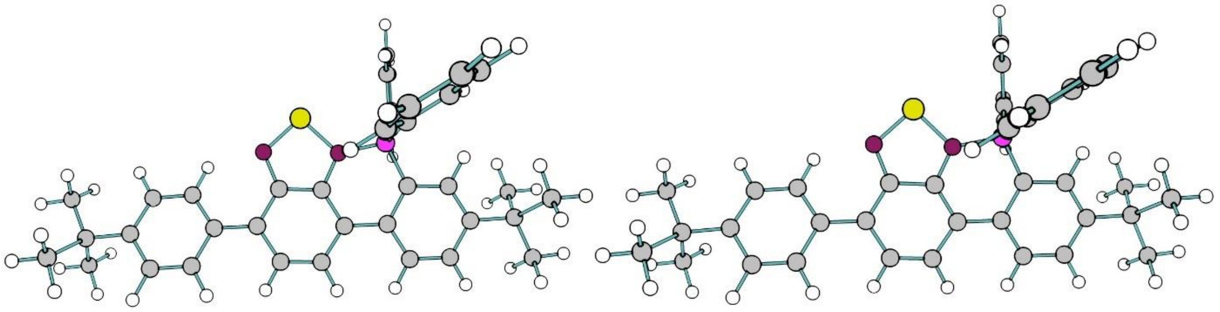

Figure S73. S1 (left) and T1 (right) structures of tBuBTZ-BP.

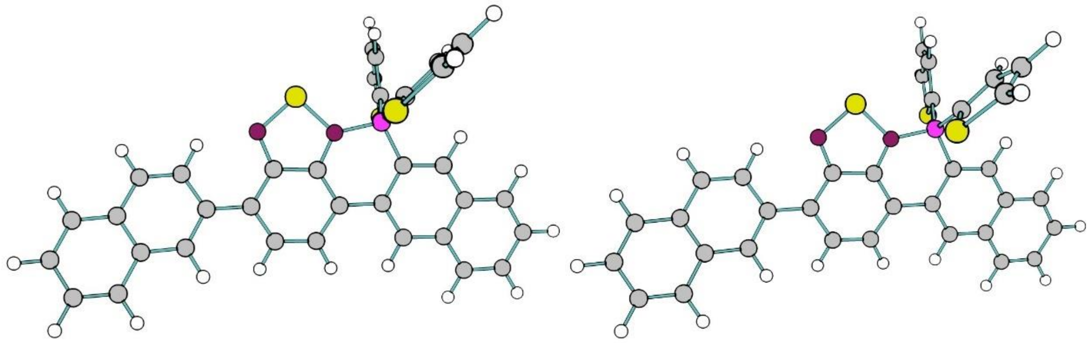

Figure S74. S1 (left) and T1 (right) structures of 2NapBTZ-BT.

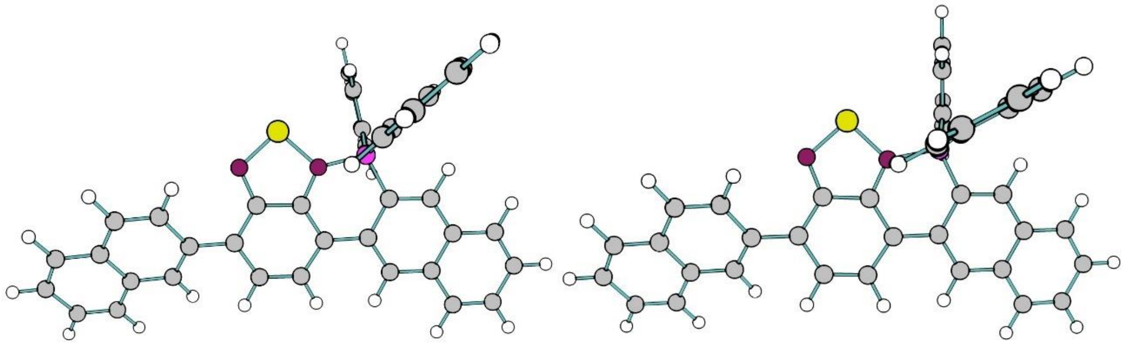

Figure S75. S1 (left) and T1 (right) structures of 2NapBTZ-BP.

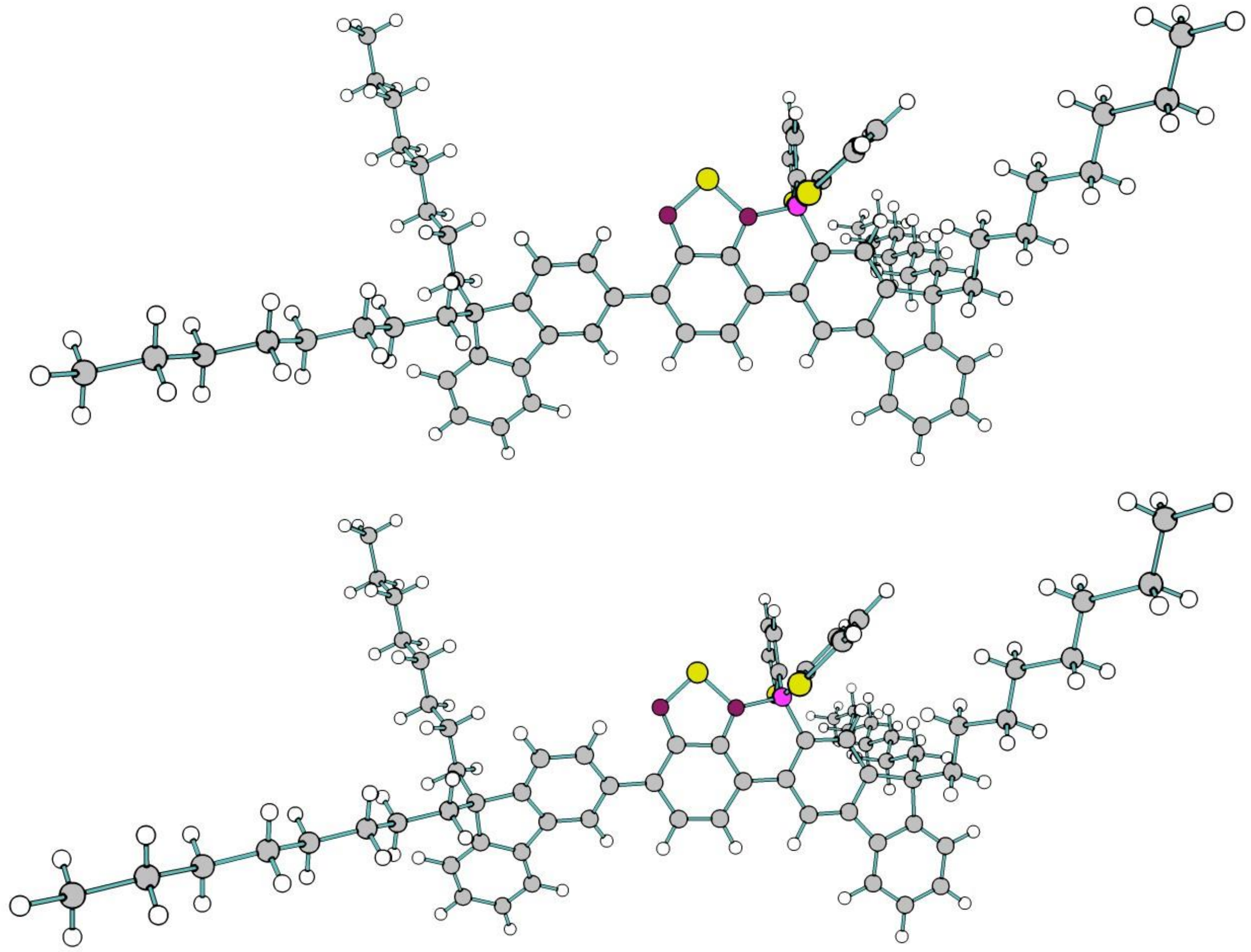

Figure S76. S1 (top) and T1 (bottom) structures of FluBTZ-BT.

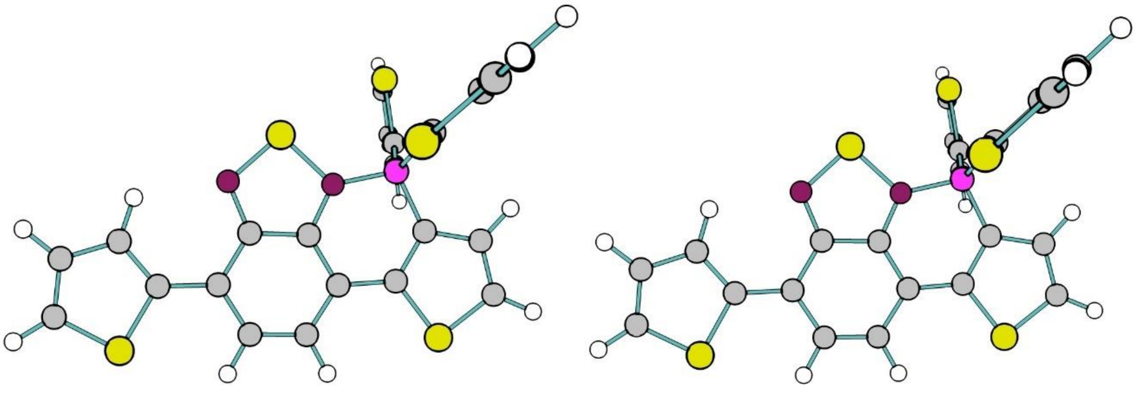

Figure S77. S1 (left) and T1 (right) structures of TpBTZ-BT.

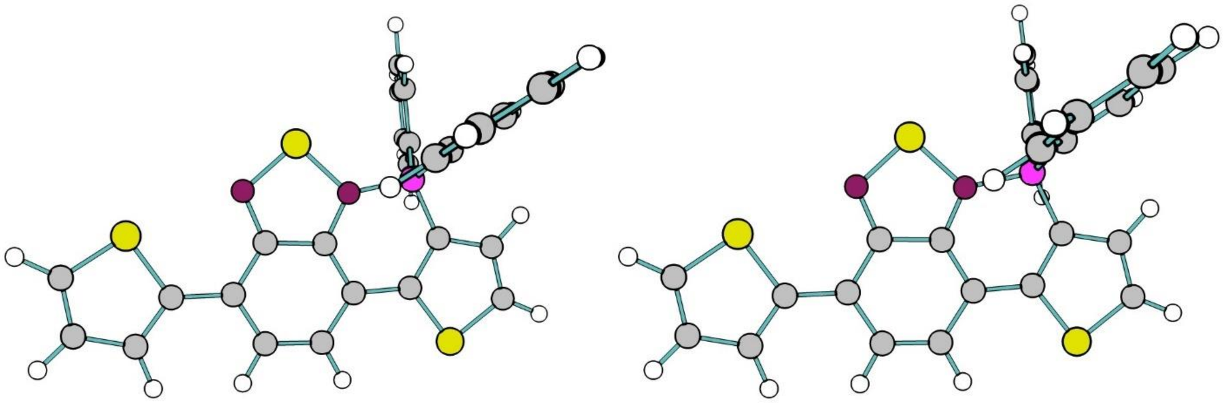

Figure S78. S1 (left) and T1 (right) structures of TpBTZ-BP.

8. Photocatalysed Phosphorylation in flow

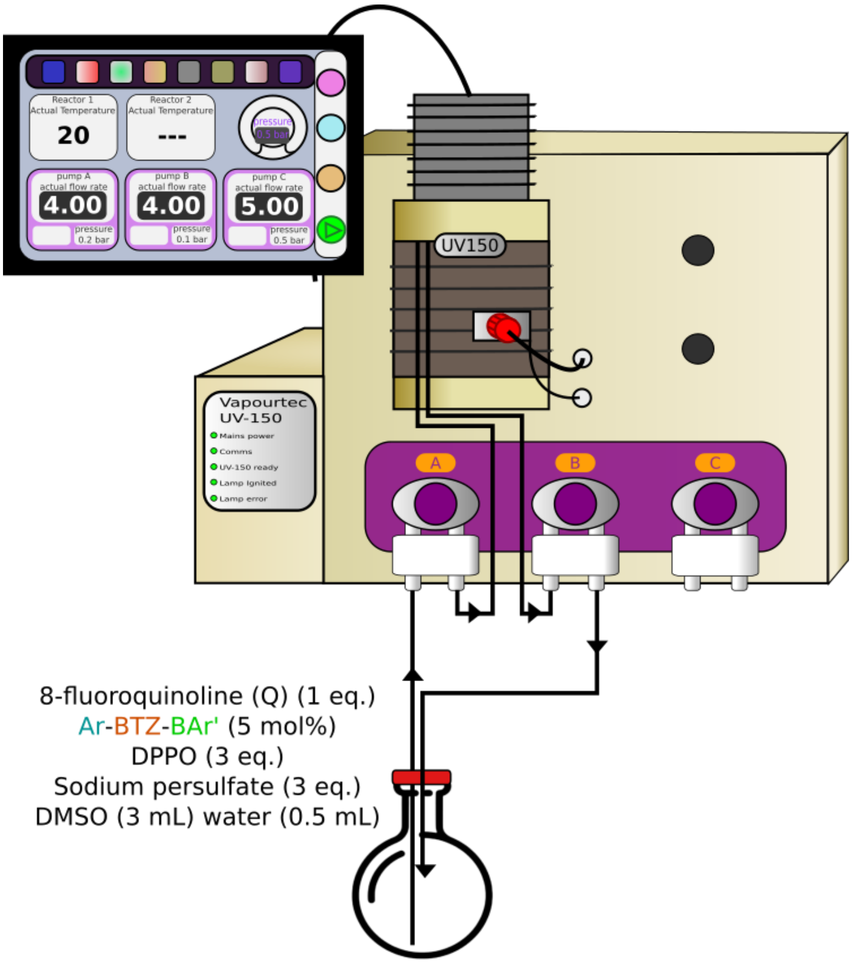

Figure S79. Flow machine set-up for the C-4 phosphorylation of Q. All the starting materials were inserted into a 1-necked round bottomed flask and N_2 was removed using a Schlenk line before the addition of the solvent. The flask was sealed and connected to pump A (4 mL/min) which pushed the reaction mixture through the flow photo-reactor. Inside the photo-reactor a 10mL transparent PTFE tube coil is placed, which guarantees homogeneous and efficient irradiation through the whole length. Finally, Pump B (4 mL/min) connected the reactor with the initial flask closing the cycle. Pump B was set to the same rate of A to prevent clogging without interfering with the pressure of the system.

Phosphorylation reaction with flow set-up

A small oven-dry glass vial containing a magnetic stirring bar was charged with 8-Fluoroquinoline (Q) (0.2 mmol, 29 mg, 1 eq.), $Na_2S_2O_8$ (0.6 mmol, 142 mg, 3 eq.), diphenylphosphine oxide (DPPO) (0.6 mmol, 121 mg, 3 eq.), and Photocatalyst (5 mol%, 0.01 mmol). The vial was closed with a rubber septum and the reaction mixture was evacuated and backfilled three times with nitrogen gas. Dimethylsulfoxide (DMSO) (3 mL) was then added as solvent and attached to the nitrogen-purged flow system with two needles for inlet and outlet. The inlet needle was connected to pump A (4 mL/min), and further in sequence to the photoreactor (10mL total volume of coil) and the second pump B (4 mL/min) to prevent clogging. Pump B then pushed the reaction mixture back in the initial vial, closing the cycle. The flow reactor was equipped with a 530 nm LED set, which shined homogeneously the reactor PTFE coil, all enclosed in a highly reflective reactor chamber with temperature control. The experiment time was 4 hours.

At the end of this time, conversion was measured by integration in a crude NMR (^{19}F NMR for Q, ^{1}H NMR for other quinoline derivatives) was performed using a 0.2 mL aliquot from the reaction mixture in 0.5 mL of

$CDCl_3$. To obtain a yield instead, the reaction mixture was quenched with saturated aqueous $NaHCO_3$ (6.0 mL), washed three times with water (20 mL x3) and DCM (20 mL x3), and organic layer collected and dried over Na_2SO_4. DCM was then removed by vacuum and product was purified by flash chromatography (Ethyl acetate/ hexane 3:1).

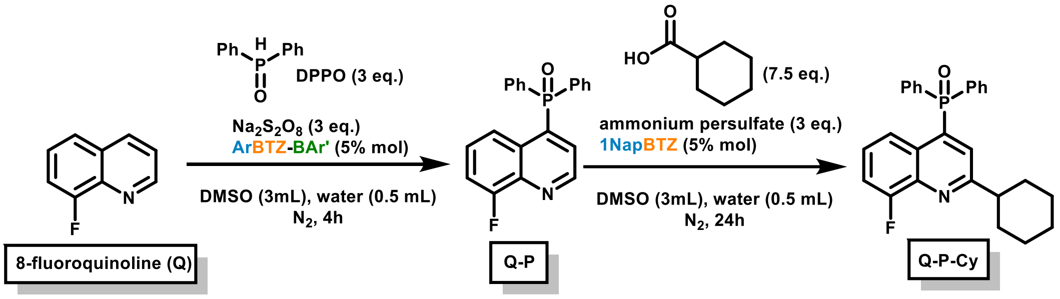

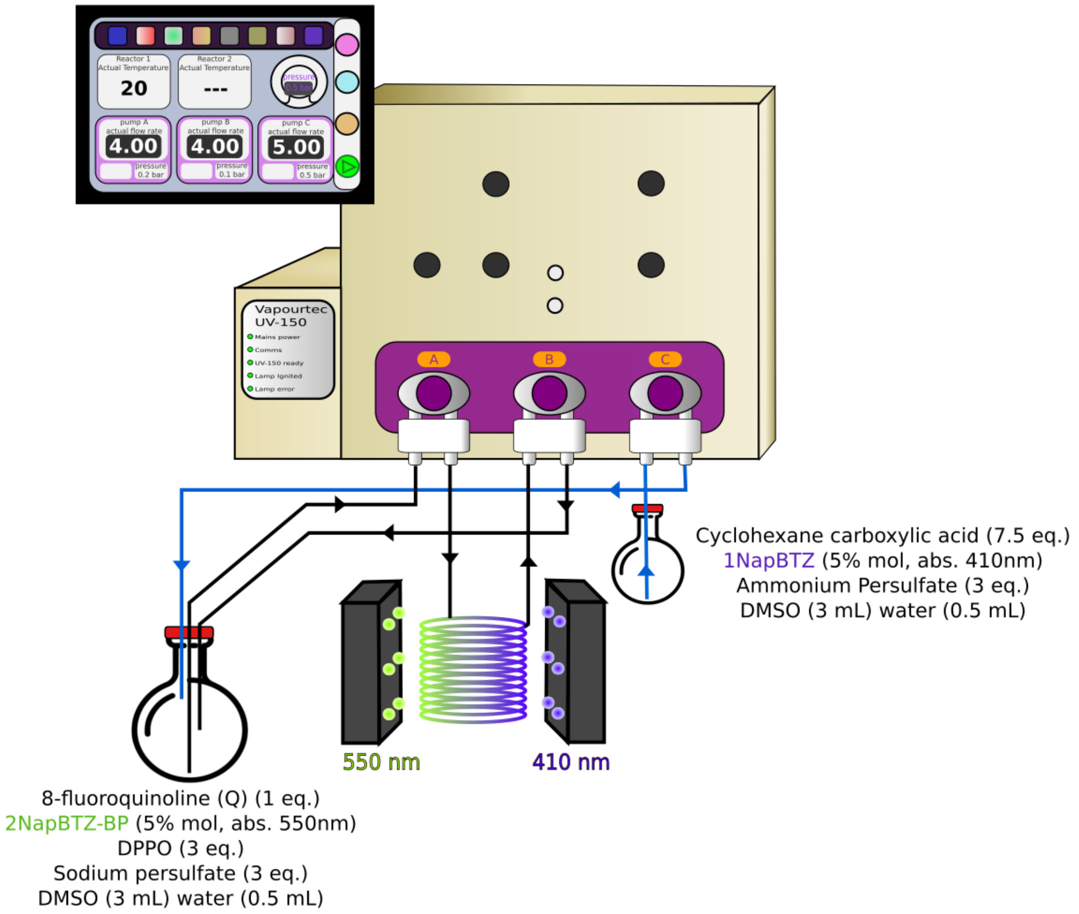

Figure S80 Tandem automated C-4 Phosphorylation and Minisci reaction scheme (top), with flow schematic diagram om). Note Pump C (blue line) and the second LED block (410 nm) were set up to automatically initiate only after 4h, to re all Q starting material was consumed prior to the Minisci step.

Sequential Phosphorylation and Minisci couplings in flow.

Phosphorylation procedure followed as described above. Following 4-hour reaction time, pump C was programmed to automatically inject Cyclohexane carboxylic acid (1.5 mmol, 192 mg, 7.5 eq.), Ammonium persulfate (0.6 mmol, 137 mg, 3 eq.) and a second photocatalyst (**1NapBTZ** 5 mol%, 0.01 mmol, 3.8 mg, abs.

410 nm)[5] in DMSO (3 mL)/ water (0.5 mL). Simultaneously, a second LED block (410 nm) was programmed to turn on at the same time, to accommodate the different functioning absorption wavelength of the second photocatalyst used. Reaction was then carried out for 24 hours at 20°C. Following this time, the reaction mixture was quenched with saturated aqueous $NaHCO_3$ (10.0 mL), washed three times with water (20 mL x3) and DCM (20 mL x3), and organic layer collected and dried over Na_2SO_4. DCM was then removed by vacuum and product was purified by flash chromatography (hexane/ Ethyl acetate 90:10), yielding a brown resinous solid product.

Q-P-Cy

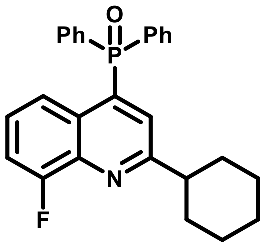

^{1}H NMR ($CDCl_3$, 400 MHz, 25.0 °C) δH 8.18 (d, 8.07 Hz, 1 H), 7.26-7.64 (m, 4 H), 7.64-7.57 (m, 2 H), 7.55-7.47 (m, 4 H), 7.40-7.30 (m, 2 H), 7.16 (d, 16.15 Hz, 1H), 1.92 (m, 2 H), 1.82 (m, 2 H), 1.73 (m, 1 H), 1.41 (m, 4 H), 1.26 (m, 2 H). **^{19}F NMR** ($CDCl_3$, 400 MHz, 25.0 °C) δF -123.77 (s). **^{31}P NMR** ($CDCl_3$, 400 MHz, 25.0 °C) δP 30.45 (s). **HRMS** (ESI) m/z: [M + H]+ Calcd for C27H26FNOP 430.1731; Found 430.1729.

Table S4. optimisation of reaction conditions for the phosphorylation of Quinoline derivatives. Optimal reaction conditions: 9-fluoroquinoline (Q) (0.2 mmol), Diphenyl phosphine oxide (0.6 mmol) Oxidant (0.6 mmol), PC (5 mol%), DMSO (3mL), deionised water (0.5 mL); reaction time: 4h; T= 26 °C ; 12 W 525 nm LED. **(a)** Conversion determined by ^{19}F NMR for Q and ^{1}H NMR for other quinoline derivatives.

Entry	PC	Conversion to product QP (%)[a]	Starting material left (%)[a]	Oxidant	Time (h)	Variation from standard conditions
1	-	13	88	$Na_2S_2O_8$	4	No PC
2	PhBTZ-BT	50	33	$Na_2S_2O_8$	4	none
3	PhBTZ-BP	65	8	$Na_2S_2O_8$	4	none
4	PhBTZ-BF	43	57	$Na_2S_2O_8$	4	none
5	tBuBTZ-BT	45	45	$Na_2S_2O_8$	4	none
6	tBuBTZ-BP	39	6	$Na_2S_2O_8$	4	none
7	2NapBTZ-BT	54	46	$Na_2S_2O_8$	4	none
8	2NapBTZ-BP	73	9	$Na_2S_2O_8$	4	none
9	FluBTZ-BT	38	62	$Na_2S_2O_8$	4	none
10	TpBTZ-BT	41	49	$Na_2S_2O_8$	4	none
11	TpBTZ-BP	63	37	$Na_2S_2O_8$	4	none
12	2NapBTZ-BP	0	100	$Na_2S_2O_8$	24	2 eq. TEMPO
13	2NapBTZ-BP	60	39	$Na_2S_2O_8$	4	10 times larger scale, same reactor
14	PhBTZ-BP	68	24	$Na_2S_2O_8$	4	40 times larger scale, 8 mmol **Q**

9. Productivity and Space time yield calculations

Values of Residence time, Productivity and Space-Time Yield (STY) were calculated as follows for the ten-fold large-scale reaction (entry 13) in flow, using the values below:[12]

Reaction Volume (35 mL); Reactor coil volume (10 mL); Flow rate (4 mL min^{-1}); Reaction time (4 hours).

Firstly, to calculate residence time, it was necessary to obtain the residence time of one length of reactor coil at the flow rate used, as well as the time required for a full cycle of the system:

$$\text{Residence time in reaction volume} = \frac{\text{Reactor coil volume (10 mL)}}{\text{Flow rate (4 mL min}^{-1})} = 2.5 \text{ min}$$

Eq. S1

Where the time to complete the full cycle of the flow system used is:

$$\text{Single cycle time} = \frac{\text{Reaction Volume (35 mL)}}{\text{Flow rate (4 mL min}^{-1})} = 8.75 \text{ min}$$

Eq. S2

Then, during each cycle, the fraction of time spent inside the reactor (residence) can be obtained as follows:

$$\text{Reactor path during cycle} = \frac{\text{Reactor coil volume (10 mL)}}{\text{Reaction Volume (35 mL)}} = 0.29$$

Eq. S3

And the number of cycles during reaction time is given as:

$$\text{Number of cycles} = \frac{\text{Reaction time (4 hours)}}{\text{Single cycle time (8.75 min)}} = 27.4 \text{ cycles}$$

Eq. S4

Thus, the residence time of the system was calculated as follows:

$$\text{Residence time} = \text{Single cycle time (min) x number of cycles x Reactor path during cycle}$$

$$\text{Residence time} = 8.75 \text{ min x } 27.4 \text{ x } 0.29 = 69.6 \text{ min} = 1.16 \text{ hours}$$

Eq. S5

Moreover, Productivity of the ten-fold system (obtained over reaction time) was calculated as follows:

$$\text{Productivity (mmol h}^{-1}) = \frac{\text{Conversion (mmol)}}{\text{Reaction Time (h)}} = \frac{1.2 \text{ mmol}}{4 \text{ h}} = 0.3 \text{ mmol h}^{-1}$$

Eq. S6

$$\text{Productivity (mg h}^{-1}) = 104.1\ \text{mg h}^{-1}$$

Hence, the Space-Time yield of the same system can be obtained as follows:

$$\text{Space} - \text{Time Yield} = \frac{\text{Yield (mmol)}}{\text{Reaction Time (h) x Reactor Volume (mL)}}$$

$$\text{Space} - \text{Time Yield} = \frac{0.97\ \text{mmol}}{4\ \text{h x}\ 10\ \text{mL}} = 0.024\ \text{mmol h}^{-1}\text{mL}^{-1}$$

Eq. S7

However, if in the STY calculation we strictly utilise the residence time rather than the total reaction time, we obtain the following value for the system:

$$\text{Space} - \text{Time Yield (res)} = \frac{\text{Yield (mmol)}}{\text{Residence time (h) x Reactor Volume (mL)}}$$

$$\text{Space} - \text{Time Yield (res)} = \frac{0.97\ \text{mmol}}{1.16\ \text{h x}\ 10\ \text{mL}} = 0.084\ \text{mmol h}^{-1}\text{mL}^{-1} = 29\ \text{mg h}^{-1}\text{mL}^{-1}$$

Eq. S8

The same calculations can be carried for the 40-fold reaction, which had 68% conversion (58% yield) in a quarter of the residence time (0.29 hours).

$$\text{Productivity (mmol h}^{-1}) = \frac{5.44\ \text{mmol}}{4\ \text{h}} = 1.36\ \text{mmol h}^{-1}$$

Eq. S9

$$\text{Space} - \text{Time Yield (res)} = \frac{4.64\ \text{mmol}}{0.29\ \text{h x}\ 0.25\ \text{x}\ 10\ \text{mL}} = 1.6\ \text{mmol h}^{-1}\text{mL}^{-1}$$

Eq. S10

10. Raw Materials Cost Analysis

Cost analysis followed from literature Analysis, Synthesis, and Deign of Chemical Process, 2008.[13]

Firstly, cost of raw materials (RM), obtained from materials and solvents for synthetic process:

4,7-Dibromobenzo[*c*][1,2,5]thiadiazole (Br_2BTZ)

Table S5. Cost analysis for **Br_2BTZ**. (a) Cost of solvents for synthesis and purification and $MgSO_4$ were combined.

Chemical	Chemical Supplier	Amount Purchased	Cost/ £	Cost/ $	Amount Used	Cost of Amount Used/$
Benzo[c][1,2,5]thiadiazole	Fluorochem	100 g	45.00	60.81	2.50 g	1.53
N-Bromosuccinimide	Sigma-Aldrich	1 kg	38.50	52.03	6.88 g	0.35
98% Sulfuric Acid	Fischer Scientific	1 L	43.70	59.05	25 mL	1.47
Solvents[a]	-	-	-	-	-	0.80

Total cost per synthesis of **Br_2BTZ** = $4.15

Mass of **Br_2BTZ** obtained = 4.52 g

Cost per gram of **Br_2BTZ** = $0.91 g^{-1} (£0.67 g^{-1})

4,7-Di(2-napthyl)benzo[*c*][1,2,5]thiadiazole (2NapBTZ)

Table S6. Cost analysis for **2NapBTZ**. (a) Cost of solventsf or synthesis and purification and $MgSO_4$ were combined (b) Cost of **Br_2BTZ** based on the calculation shown above.

Chemical	Chemical Supplier	Amount Purchased	Cost/ £	Amount Used/ g	Cost of Amount Used/ £
**Br_2BTZ**[b]	-	1 g	0.67	0.141	0.09
Benzene boronic acid	Fluorochem	25 g	16.00	0.215	0.27
$Pd(PPh_3)_4$[b]	Fluorochem	1 g	17.00	0.023	0.53
Potassium carbonate	Fluorochem	500 g	14.00	0.14	0.01
Solvents[a]	-	-	-	-	0.76

Total cost per synthesis of **2NapBTZ** = $2.25 (£1.66)

Mass of **2NapBTZ** obtained = 0.163 g

Cost per gram of **2NapBTZ** = $13.78 g^{-1} (£10.18 g^{-1})

4,7-Di(2-napthyl)benzo[*c*][1,2,5]thiadiazole (2NapBTZ-BP) borylation and transmetallation

Table S7. Cost analysis for **2NapBTZ-BP** (a) Cost of solvents for synthesis and purification were combined (b) Cost of **2NapBTZ** based on the calculation shown above.

Chemical	Chemical Supplier	Amount Purchased	Cost/ £	Amount Used	Cost of Amount Used/£
**2NapBTZ**[b]	-	1 g	10.18	0.082 g	0.83
BCl_3	Sigma	100mL	85.50	1.2 mL	1.03
Tributyl phenylstannane	Sigma	10 g	109.00	0.16 mL	1.64
$AlCl_3$	Sigma	100 g	33.5	3 mg	0.01
Solvents[a]	-	-	-	-	0.45

Total cost per synthesis of **2NapBTZ-BP** = $5.36 (£3.96)

Mass of **2NapBTZ-BP** obtained = 0.086 g (78% yield)

Cost per gram of **2NapBTZ-BP** = $61.69 g^{-1} £46.05 g^{-1}

Cost per use of **2NapBTZ-BP** = \$0.31 /0.01mmol use (£0.23/0.01 mmol use)

Following this, Manufacturing costs include Waste treatment of the plant (WT), utilities (UT), and operating labour (OL), before applying Fixed capital investment (FCI).

Reference purchase pricing for commercially available PC's (relative supplier)

Cost per gram of **4CzIPN** (Sigma Aldrich) = \$5,010 g^{-1} (£3,740 g^{-1})

Cost per gram of **Ir(p-F-ppy)$_3$** (Sigma Aldrich) = \$2,530 g^{-1} (£1,870 g^{-1})

Cost per gram of **Ir(ppy)$_3$** (Sigma Aldrich) = \$1,908 g^{-1} (£1,424 g^{-1})

Cost per gram of **[Ir(df(CF$_3$)ppy)$_2$(4,4'-(OMe)$_2$bpy]BF$_4$** (Sigma Aldrich) = \$2,896 g^{-1} (£2,140 g^{-1})

Cost per gram of **1,3,5,7-Tetramethyl-8-phenyl-4,4-difluoroboradiazaindacene** (Sigma Aldrich) = \$1,938 g^{-1} (£1,432 g^{-1})

Cost per gram of **Ir(ppy)$_3$** (Ossila) = \$2,679 g^{-1} (£2,000 g^{-1})

Cost per gram of **Ir(ppy)$_3$** (TCI) =\$2,492 g^{-1} (£1,860 g^{-1})

11. References

1 C. M. S. Jones, A. Gakamsky and J. Marques-Hueso, *Sci. Technol. Adv. Mater.*, 2021, **22**, 810–848.

2 N. A. Romero and D. A. Nicewicz, *Chem. Rev.*, 2016, 116, 10075–10166.

3 E. M. Espinoza, J. A. Clark, J. Soliman, J. B. Derr, M. Morales and V. I. Vullev, , DOI:10.1149/2.0241905jes.

4 H. G. Roth, H. G. Roth, N. A. Romero and D. A. Nicewicz, 2016, 714–723.

5 D. Taylor, T. Malcomson, A. Zhakeyev, S.-X. Cheng, G. M. Rosair, J. Marques-Hueso, Z. Xu, M. J. Paterson, S. J. Dalgarno and F. Vilela, *Org. Chem. Front.*, 2022, **9**, 5473–5484.

6 D. S. Pedersen and C. Rosenbohm, *Synthesis (Stuttg).*, 2001, 2431–2434.

7 H. Ishitani, H. Suzuki, Y. Saito, Y. Yamashita and S. Kobayashi, *European J. Org. Chem.*, 2015, **2015**, 5485–5499.

8 D. L. Crossley, R. Goh, J. Cid, I. Vitorica-Yrezabal, M. L. Turner and M. J. Ingleson, *Organometallics*, 2017, **36**, 2597–2604.

9 D. L. Crossley, I. Vitorica-Yrezabal, M. J. Humphries, M. L. Turner and M. J. Ingleson, *Chem. - A Eur. J.*, 2016, **22**, 12439–12448.

10 G. R. Fulmer, A. J. M. Miller, N. H. Sherden, H. E. Gottlieb, A. Nudelman, B. M. Stoltz, J. E. Bercaw and K. I. Goldberg, *Organometallics*, 2010, **29**, 2176–2179.

11 N. Elgrishi, K. J. Rountree, B. D. McCarthy, E. S. Rountree, T. T. Eisenhart and J. L. Dempsey, *J. Chem. Educ.*, 2018, **95**, 197–206.

12 A practical guide to Catalyst Testing, Catalytica, 1987.

13 Turton, R., Bailie, R. C., Whiting, W. B, Shaeiwitz and Joseph A., *Analysis, Synthesis, and Deign of Chemical Process*, 2008.
